# Draft Genome Sequence of Multidrug-Resistant *Acinetobacter baumannii* smu isolated from a Bloodstream Infection in Sikkim, India

**DOI:** 10.7150/jgen.130521

**Published:** 2026-05-01

**Authors:** Rekha Sharma, Rohit Das, Yezey Rikzing Bhutia, Neha Pradhan, Anil Bhattarai

**Affiliations:** 1 Department of Microbiology, Sikkim Manipal Institute of Medical Sciences, Sikkim Manipal University, 5 th Mile, Tadong, Gangtok, Sikkim 737102, India.; 2 Department of Microbiology, Sikkim University, 6 th Mile Samdur, Tadong -737102, Gangtok, Sikkim, India.; 3 Department of Medical Biotechnology, Sikkim Manipal Institute of Medical Sciences, Sikkim Manipal University, 5 th Mile, Tadong, Gangtok, Sikkim 737102, India.

**Keywords:** septicemia, *Acinetobacter baumannii* smu, multidrug-resistance, whole-genome sequencing, pangenome analysis

## Abstract

Septicemia remains a major cause of death in intensive care units and its treatment is increasingly challenged by multidrug-resistant pathogens. This study investigated bloodstream infections in 89 ICU patients at a tertiary hospital in Sikkim where the Neonatal ICU showed the highest burden. *Acinetobacter baumannii* was the predominant pathogen, including 13 MDR isolates. One highly resistant isolate, *A. baumannii* smu was selected for whole-genome sequencing. The smu strain had a genome size of 4.0 Mbp, assembled into three contigs with 182× coverage, an N50 of 3.98 Mb, and a GC content of 39.16%. Nineteen resistance genes were identified including β-lactamases (*ADC-15B, OXA-98*), aminoglycoside-modifying enzymes, and multiple efflux pumps. Virulence factors included acinetobactin-mediated iron uptake, biofilm-associated genes, OmpA, and phospholipases. The genome also harbored mobile genetic elements including insertion sequences and prophages. Pangenome analysis revealed 13 distinct genes associated with stress, tolerance, stability of plasmids, and possible resistance. This study provides a comprehensive genomic characterization of an MDR *A. baumannii* isolate from Sikkim, revealing its extensive resistance and virulence features, and providing baseline genomic data to support improved antibiotics use and infection control in the region.

## 1. Introduction

*Acinetobacter baumannii* is a clinically important Gram-negative bacterium that causes severe infection of blood stream, respiratory tract, and other parts of the body. These infections predominantly occur in hospitalized, immunocompromised, and critically ill patients particularly those admitted to intensive care units (ICUs), and are associated with high morbidity and mortality especially among neonates 1. Bloodstream infections caused by *A. baumannii* remain a major clinical challenge in ICUs worldwide and continue to complicate infection prevention efforts. The organism's ability to persist in hospital environments, colonize medical devices and surfaces, and rapidly acquire resistance to multiple classes of antibiotics has contributed to its successful spread in healthcare sectors. These features together with limited treatment options frequently result in poor clinical outcomes and high fatality rates among critically ill and neonatal patients 2. Evidence from tertiary care hospitals in Northern India indicates that *Acinetobacter* species contribute substantially to culture-proven neonatal sepsis often in the range of 10-15%, sometimes higher with *A. baumannii* being the predominant species 3. A large proportion of these isolates are multidrug-resistant (MDR) showing resistance to cephalosporins, aminoglycosides, fluoroquinolones, and carbapenems. For example, a study from Northern India reported an Acinetobacter-associated sepsis rate of 13.7% with 98% of isolates identified as *A. baumannii* and nearly 96% exhibiting MDR 4.

Whole-genome sequencing has become an important approach for understanding the genetic basis of antimicrobial resistance, pathogenicity, and adaptability in *A. baumannii* 5,6 Genome level information enables the identification of resistance-associated genes and provides insights into genome organization and evolutionary features, while also facilitating comparisons among isolates from different regions. Although a growing number of *A. baumannii* genomes are available worldwide, data from several geographically distinct and underrepresented regions remain limited 7. The Himalayan region of India including the state of Sikkim represents one such underexplored area with limited publicly available genomic information on clinically important bacterial pathogens. Considering the region's distinct geography and healthcare practices, genomic characterization of MDR *A. baumannii* isolates is necessary to generate baseline reference data that can support future comparative and molecular epidemiological studies.

In this context, the present study reports the first draft genome sequence of a MDR *A. baumannii* smu isolated from a bloodstream infection in Sikkim, India. Using Illumina-based whole-genome shotgun sequencing, we generated a high-quality draft assembly and performed genome annotation. The genome sequence has been deposited in public databases to serve as a valuable genomic resource for future studies on antimicrobial resistance, pathogen evolution, and clinical microbiology in the Himalayan region of Sikkim.

## 2. Materials and Methods

A cross-sectional study was conducted for the period of 6 months (September 2024 to March 2025) to identify the bacteria and analyse antibiotic susceptibility pattern from blood culture received from the different ICUs of tertiary care hospital. Basic demographic details (age and gender) were obtained from test prescriptions, while additional clinical information was retrieved from the patient's medical records at CRH. Blood samples were collected after obtaining written informed consent in a language understood by the patients. The samples and collected data were used exclusively for research purposes, maintained under strict confidentiality, and were accessible only to authorized researchers. Identifiable patient information was not disclosed, and numerical codes were used instead of names to ensure privacy. The study was approved by the Institutional Research Committee (IRC) (IRC certificate number IRC/2024-137) of Sikkim Manipal Institute of Medical Sciences (SMIMS), and all procedures followed institutional ethical guidelines and regulations.

### 2.1 Sample collection and bacterial isolation

Blood samples received from various ICUs for the routine culture and sensitivity testing to the department of Microbiology during the study period were considered. The blood samples of 2-10 ml volume were collected following strict aseptic procedure in a Bact/Alert aerobic blood culture bottles. They were processed in a Bact/Alert (BioMerieux, France; http://www.biomerieux.com) as per the standard operation procedure of the laboratory. Once a culture bottle flagged positive, the sample was streaked onto blood agar and MacConkey agar plates and incubated aerobically at 37^0^C for 24-48 hours. The resulting bacterial isolates were then collected for further analysis.

### 2.2 Identification and antibiotic susceptibility profiling of bacterial pathogens

Once visible growth was observed on the culture plates, the isolates were subjected to Gram's staining and biochemical tests for preliminary identification of the pathogens. The final identification and antibitoic susceptibility testing was then performed using the automated VITEK-2 compact system (BioMerieux, France; http://www.biomerieux.com), following CLSI guidelines. Bacterial suspension equivalent to 0.5 McFarland turbidity was prepared, out of which 155 µL of suspension was used with the VITEK 2 GN card for bacterial identification, and 145 µL was subjected to VITEK 2 AST-N405 card for antimicrobial susceptibility testing. Results were obtained after 24 hours. VITEK-2 system determines drug sensitivity using the broth dilution method. Antimicrobial susceptibility was tested against 16 antimicrobial agents namely Tazobactam, Ceftazidime, Cefoperazone, Cefepime, Aztreonam, Imipenem, Meropenem, Amikacin, Gentamicin, Ciprofloxacin, Levofloxacin, Minocycline, Tigecycine, Colistin, Fosfomycin, and Trimethoprim. Bacteria resistant to three or more (≥3) drugs belonging to different classes were classified as MDR. Stock cultures of each isolate were maintained on nutrient agar slants and stored at 4^0^C. Among all the bacterial isolates, the strain exhibiting the highest level of drug resistance was selected for whole-genome sequencing and analysis.

### 2.3 Whole-genome sequencing, assembly and annotation

The MDR *A. baumannii* isolate was cultured in LB broth at 37^0^C and bacterial cells were harvested by centrifugation at 5000 x g for 10 minutes. Genomic DNA was extracted using the QIAamp DNA Mini Kit (Qiagen, Hilden, Germany). DNA quality and concentration were quantified through Qubit fluorometer and NanoDrop 2000 spectrophotometer (Thermo Fisher Scientific, Massachusetts, USA). Sequencing libraries were prepared using the NEBNext Ultra DNA Library Prep Kit (New England Biolabs, Ipswich, MA, USA), and sequenced on the NovaSeq 6000 platform (Illumina, San Diego, CA, USA) with a paired-end strategy. After sequencing, raw reads were quality-checked through FastQC v0.11.9 8 and trimmed with Trim Galore v0.6.11 9. Genome assembly was carried out through SPAdes v4.1.0 10 followed by scaffolding with Ragout 11, and gap filling using the TGS-GapCloser 12. Genome assembly completeness was evaluated using BUSCO v6 13 in *prok_genome_prod mode*. The analysis was performed using the lineage-specific dataset acinetobacter_odb12 which was found to be more appropriate for *A. baumannii*. Gene prediction during BUSCO analysis was performed using Prodigal v2.6.3. 14. Additionally, Prodigal v2.6.3 was employed for structural annotation to predict protein-coding genes in the bacterial genome. Functional annotation was performed using DIAMOND v2.1.14 against the NCBI nr database 15. tRNA and rRNA genes were predicted using tRNAscan-SE v2.0 16 and Barrnap https://github.com/tseemann/barrnap respectively.

### 2.4 Prediction of antibiotic resistance genes, virulence factors, and mobile genetic elements (MGEs)

The draft genome of *A. baumannii* smu was analyzed to identify key genetic features associated with antibiotic resistance, pathogenicity, and defense mechanisms. Antimicrobial resistance (AMR) genes were identified using the CARD Database 17, while virulence factors were detected through BLAST searches against the Virulence Factor Database 18. Further to assess the contribution of mobile genetic elements to genome plasticity and resistance, insertion sequences (IS elements) were identified using ISEScan v1.7.2 19, and prophage regions were predicted with PHASTER 20.

### 2.5 Pangenome analysis

The coding sequence prediction and gene annotation of the *A. baumannii* smu strain were performed using Proksee 21. The pangenome of the smu strain was analyzed through Roary v3.13.0 22 in comparison with 22 other *A. baumannii* strains downloaded from the EzTaxon server 23. OrthoFinder v2.5.4 24 was used to identify orthologous genes across the genomes. The overall genome quality of all strains was assessed with CheckM v1.2.2 25. Phylogenetic relationships were analyzed using FastTree v2.1 26 with 500 bootstrap replicates, and the resulting tree was visualized and edited in iTOL 27.

## 3. Results

### 3.1 Genome sequencing, assembly and annotation statistics

**Figure 1:**
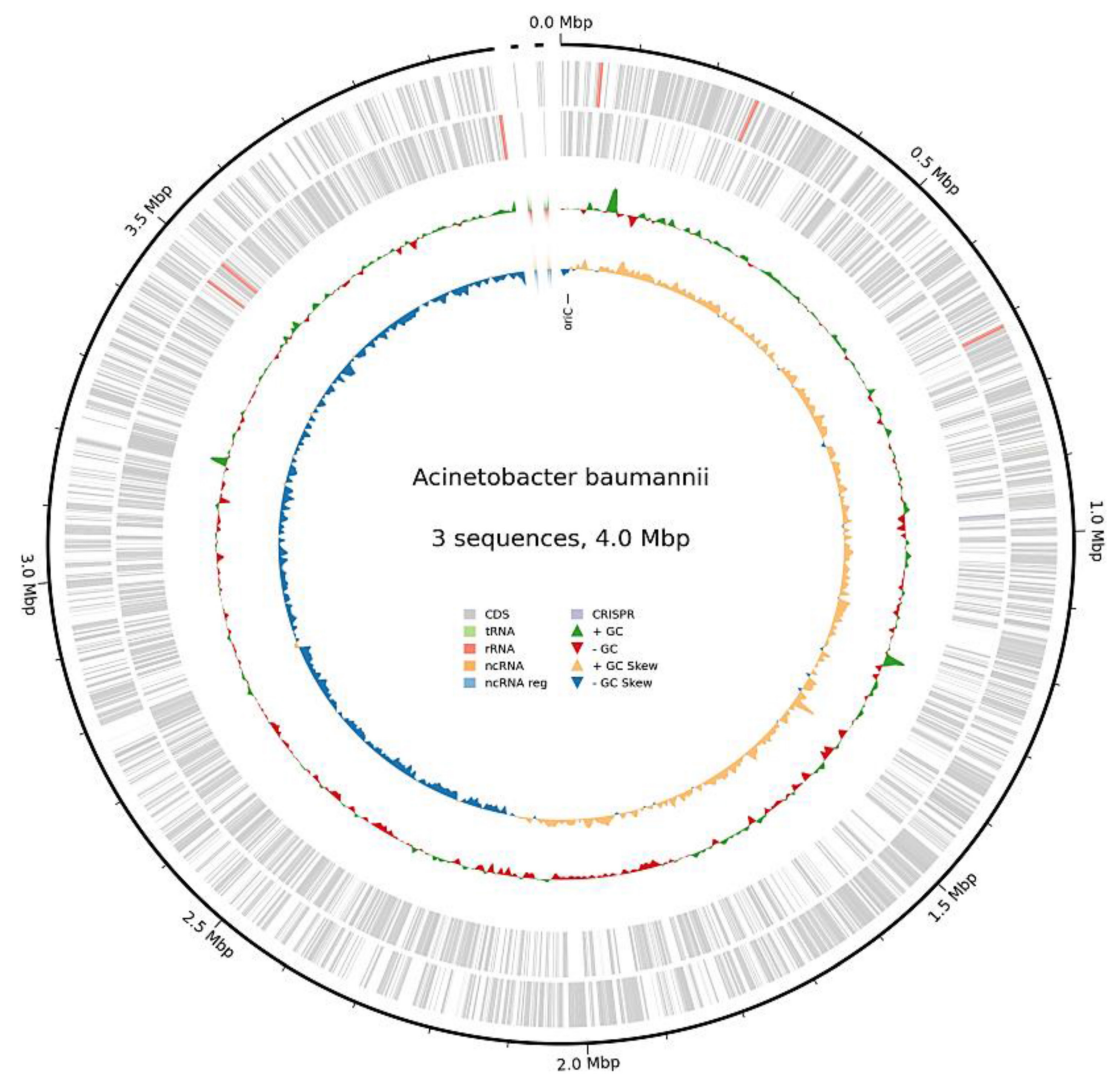
A circular genome map of MDR *A. baumannii* smu.

The whole-genome sequencing of MDR *A. baumannii* smu generated 4,853,348 high-quality reads. The assembled genome had a total size of 3,999,148 bps (4.0 Mbp), as shown in Figure 1, with an average sequencing depth of 182.1× coverage. The draft genome was resolved into three contigs with an N50 value of 3,981,953 bp and a GC content of 39.16%. The L50 and L90 values were both 1 indicating that a single contig accounted for the majority of the assembly. The largest contig measured 3981953 bp, while the smallest contig was 7,655 bp in length. BUSCO analysis of acinetobacter_odb12 showed that 98.8% (1468/1486) of the BUSCO genes were complete of which 98.7% (1466) were complete and single-copy and 0.1% 2 were complete and duplicated. In addition to that, 9% (0.6%) of the BUSCO genes were identified as fragmented, while 0.6% 9 were missing. These findings suggest a high level of completeness of the assembled genome. The details statistics of BUSCO analysis are provided in the **Supplementary Table 1A.** Further, genome annotation predicted a total of 3,726 protein-coding genes along with 18 rRNA and 76 tRNA genes (Table 1).

### 3.2 Prediction of antibiotic resistance genes in MDR *A. baumannii* smu

**Figure 2:**
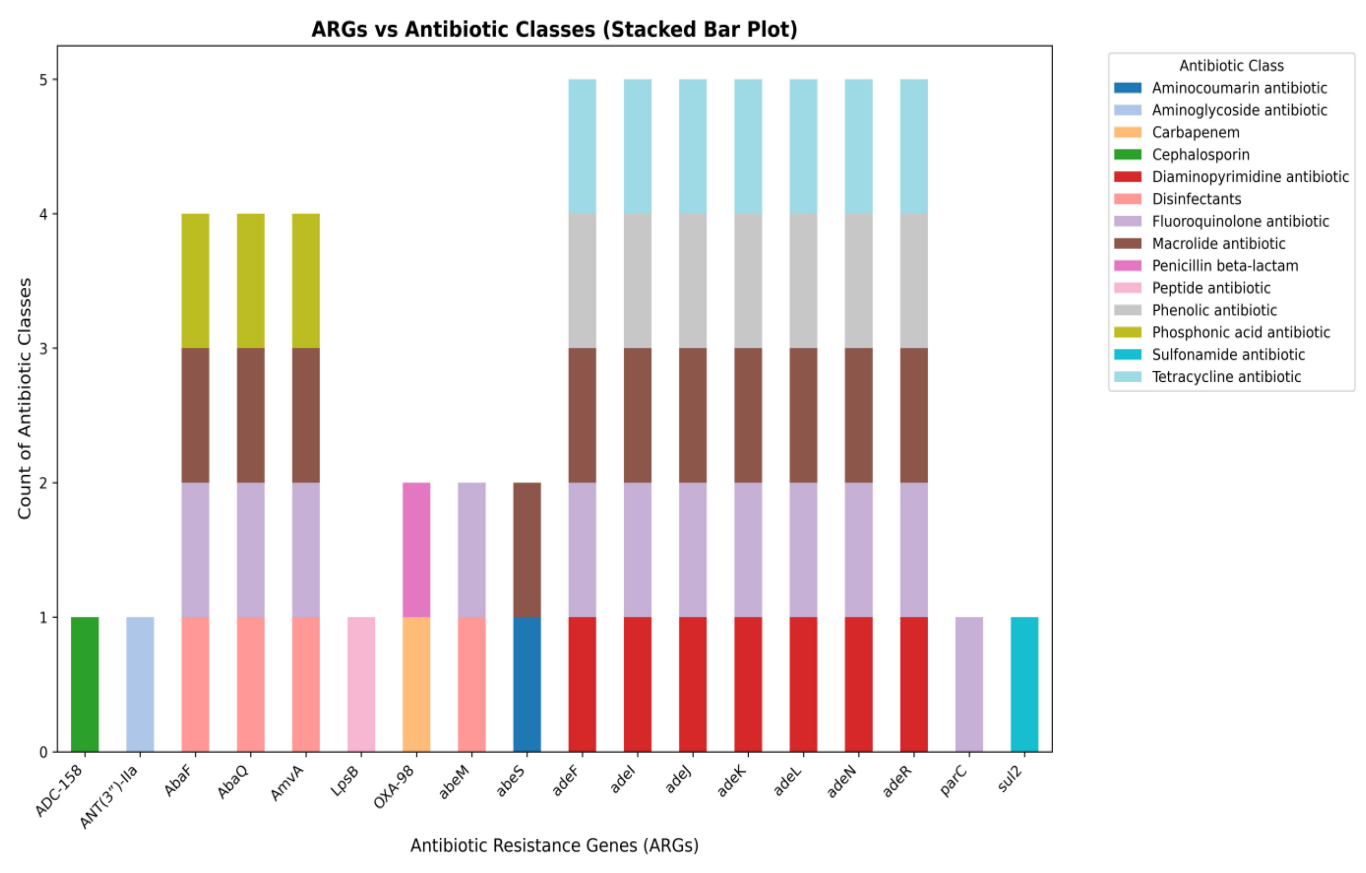
** A stacked bar plot showing the distribution of AMR genes across different antibiotic classes in MDR *A. baumannii* smu.** Efflux pump genes (*AbaF, AbaQ, AmvA, AdeF, AdeI, AdeJ, AdeK, AdeL, AdeN, AdeR*) were dominant, while genes associated with inactivation (*ADC-158, ANT(3”)-IIa*, target alteration (*parC*), and target replacement (*sul2*) were less represented.

Whole-genome assembly of the MDR *A. baumannii* smu revealed the presence of diverse ARGs conferring resistance to multiple antibiotic classes. A total of 18 distinct ARGs were identified in the draft genome of the smu strain, distributed across multiple antibiotic classes. The stacked bar plot is used to demonstrate the distribution of ARGs in the various categories of antibiotics (Figure 2). We observed that genes like *ADC-15B* and *ANT(3”)-IIa* genes were linked to cephalosporin and aminoglycoside resistance respectively while *OXA-98* and *LpsB* were associated with β-lactam and peptide antibiotics resistance. Several efflux pump genes such as *abeM, abeS, AbaF, AbaQ, AmvA, adeF, adeI, adeJ, adeK, adeL, adeN, adeR* showed a strong association with MDR conferring resistance to peptide antibiotics, aminocoumarins, macrolides, fluoroquinolones, phenolics, disinfectants, sulfonamides, phosphonic acid antibiotics, and tetracyclines. Among them, the RND efflux pump operon genes (*adeF, adeI, adeJ, adeK, adeL, adeN, adeR*) showed the widest range of resistance, each conferring resistance to five different antibiotics including trimethoprim (Diaminopyrimidine antibiotic), fluoroquinolones, macrolides, phenolic antibiotics, and tetracyclines, highlighting their role in MDR. The efflux transporters *AbaF, AbaQ,* and *AmvA* were linked to resistance against peptide antibiotics, fluoroquinolones, macrolides, and phosphonic acid antibiotics. While genes such as *abeM* and *abeS* were actively involved in targeting peptide antibiotics, aminocoumarins, macrolides, fluoroquinolones, and sulfonamides. Other resistance genes such as *parC* and *sul2* which confer resistance to fluoroquinolones and sulfonamides were detected but appeared to be less prominent.

**Figure 3:**
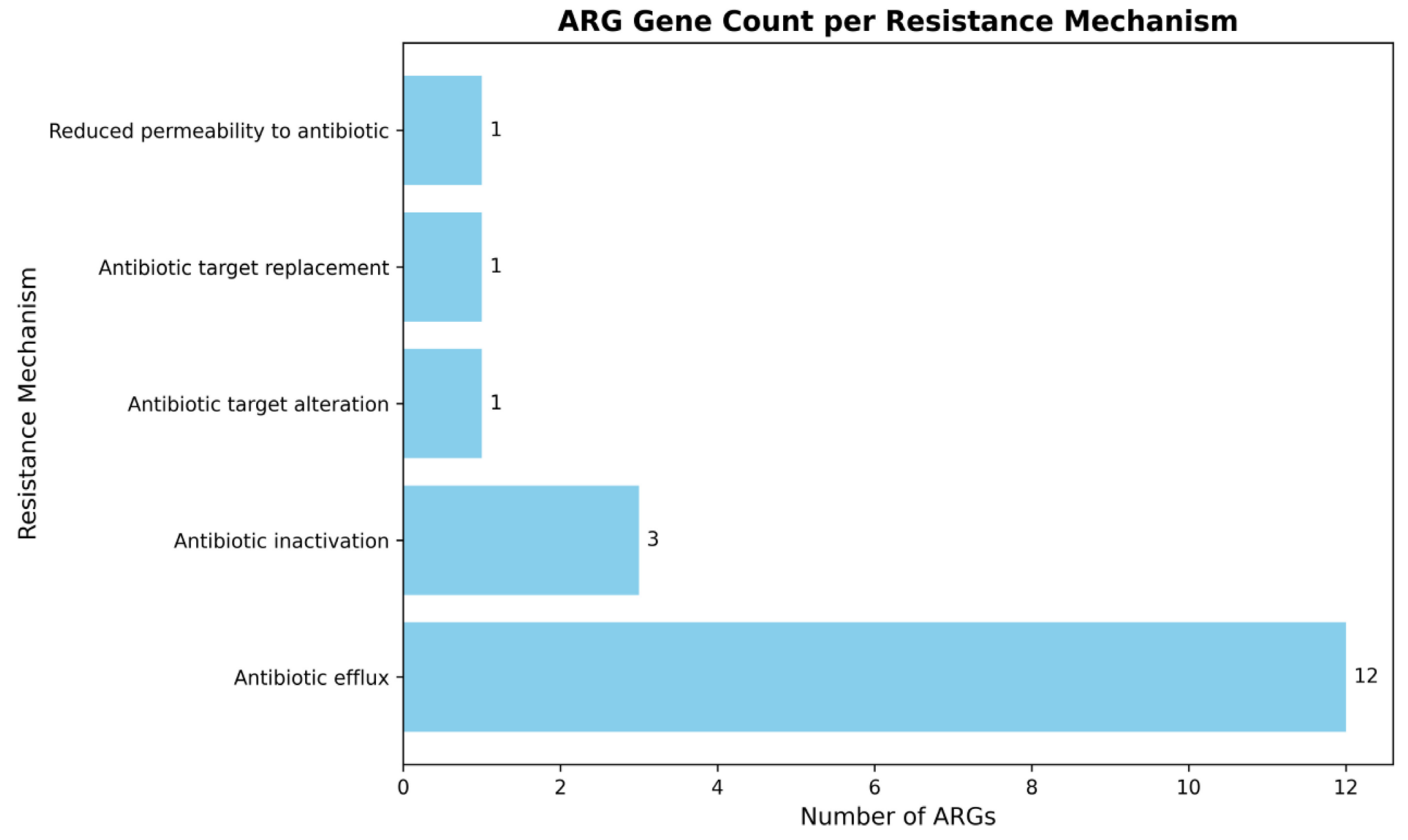
**Classification of ARG genes by resistance mechanisms in *A. baumannii* smu.** Efflux pumps were the predominant resistance mechanism, while inactivation, target alteration, replacement, and reduced permeability were less common.

To further characterize the underlying resistance mechanisms, the identified ARGS were classified based on their resistance strategies (Figure 3). The majority of genes encoded efflux systems comprising of twelve genes, highlighting efflux-mediated MDR as the dominant mechanism in this strain. In contrast, antibiotic inactivation mechanisms represented by β-lactamases and aminoglycoside modifying enzymes were encoded by three genes. Single genes were identified for antibiotic target alteration, target replacement, and reduced permeability, indicating these as less prevalent mechanisms in *A. baumannii* smu. A summary of representative AMR genes identified in the MDR *A. baumannii* smu is provided in Suppl Table 1B.

### 3.3 Identification of virulence factors in *A. baumannii* smu

**Figure 4:**
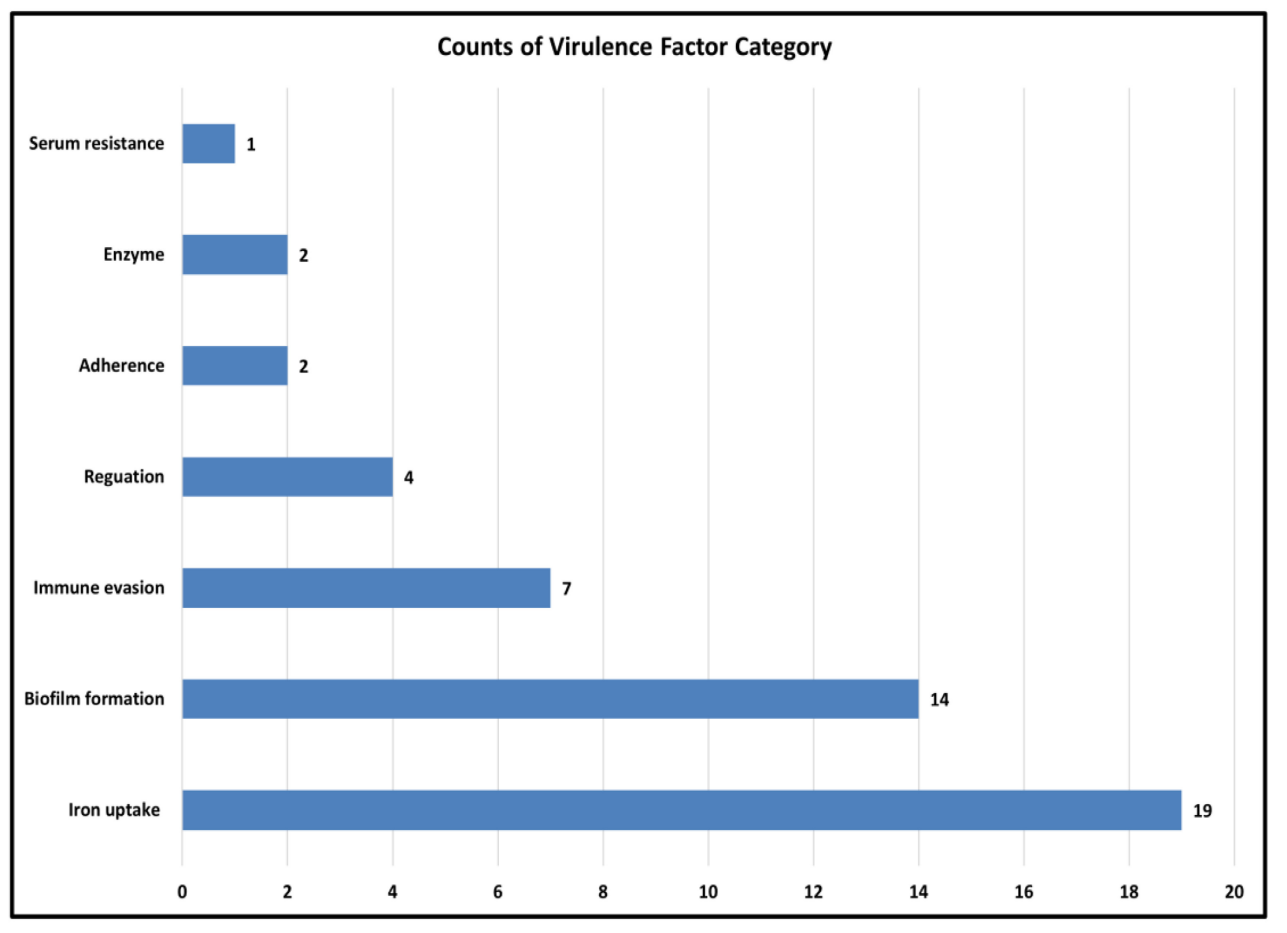
**Distribution of predicted virulence factor categories in the genome of *A. baumannii* smu.** A total of 58 genes were classified into 7 categories using VFDB with iron uptake, biofilm formation, and immune evasion being the most significant.

Virulence factors are molecules that help bacteria survive, spread, and cause disease 28. As *A. baumannii* is a major cause of hospital-acquired infections where virulence traits often act together with antibiotic resistance to increase severity. Therefore, predicting these factors is crucial for understanding its pathogenic potential. In the smu strain, virulence factors were grouped into major functional categories (Figure 4). The largest cluster was found for the iron uptake system (19 genes) then for the biofilm formation (14 genes), which indicates their role in the survival and persistence in the host. Other categories included immune evasion (7 genes) and regulation (4 genes), while smaller contributions came from adherence (2 genes), enzymatic activity (2 genes), and serum resistance (1 gene), as depicted in the Figure.

**Figure 5:**
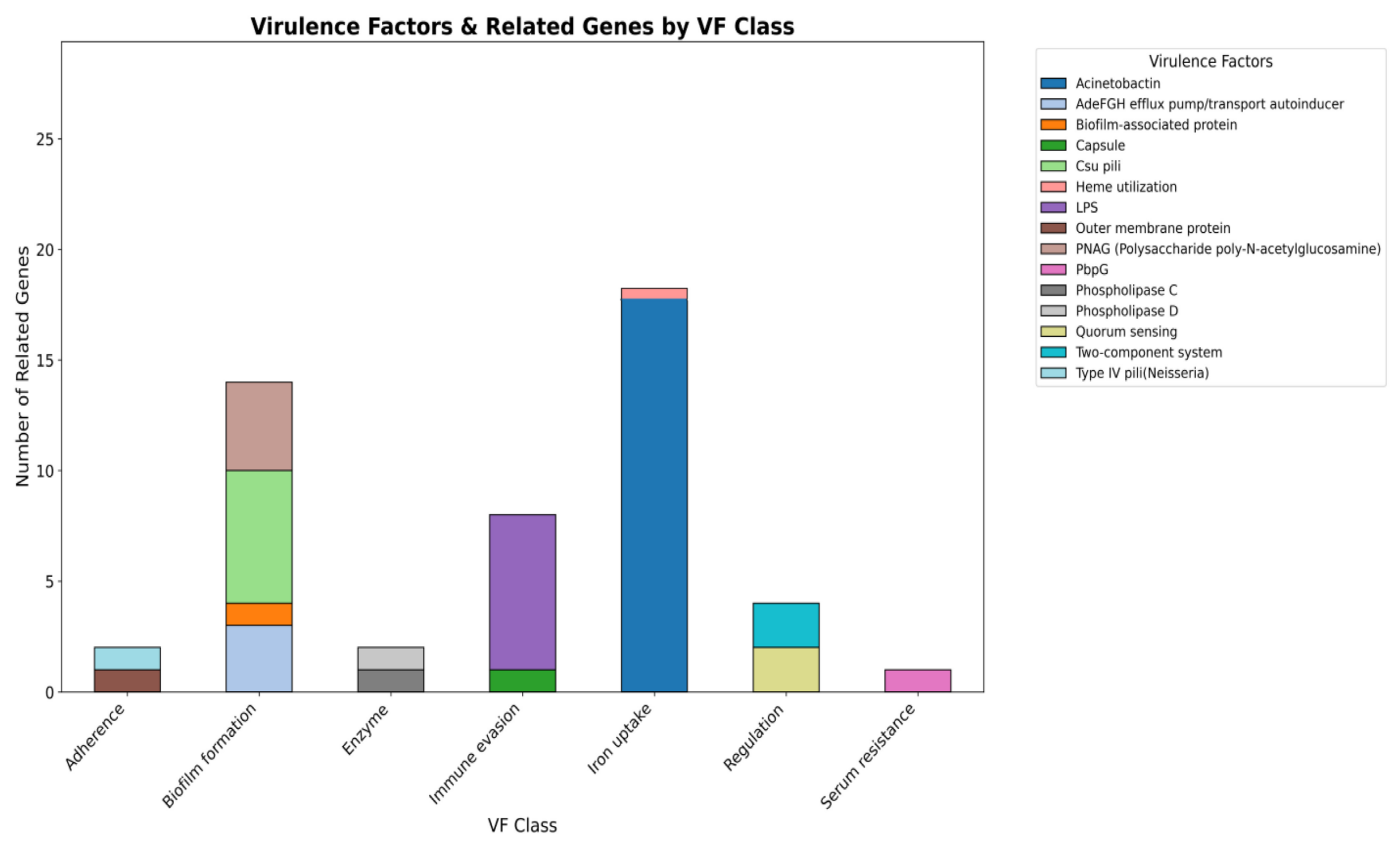
**Class-level distribution of predicted virulence factors in *A. baumannii* smu*.*** Iron uptake systems were most abundant followed by biofilm formation, immune evasion. OmpA, Type IV pili, and phospholipases contributed to adhesion and tissue invasion. Quorum sensing, two component systems, and PbpG were linked to regulation and serum resistance.

In addition to broad functional categories, several representative virulence factors were predicted in the *A. baumannii* smu strain (Figure 5). The most prominent were iron uptake systems represented by acinetobactin and heme utilization which are critical for obtaining iron under host-imposed nutrient limitation. Multiple factors supporting biofilm formation were detected including the AdefGH efflux pump/autoinducer transport system, biofilm-associated protein (Bap), csu pili, and PNAG-mediated exopolysaccharide production. The presence of a capsule and lipopolysaccharide (LPS) served as protective features that enhance persistence and immune evasion. Outer membrane protein A (OmpA) and Type IV pili contribute to host cell adhesion and damage, while phospholipase D and phospholipase C facilitate tissue invasion and host cell disruption. Quorum sensing and two-component regulatory systems were identified and are linked to virulence regulation. Serum resistance is represented by PbpG, a penicillin-binding protein that can protect against complement-mediated killing in human serum. Overall, the identified virulence factors strongly suggest the potential of *A. baumannii* smu strain in bloodstream infections. Iron acquisition systems support survival under host-imposed iron limitation while biofilm formation, capsule, and LPS enhance persistence and immune evasion. Adhesins, phospholipases, and regulatory systems further facilitate host interaction, tissue invasion, and adaptation during bloodstream infection. A more detail on the virulence factor classes, associated mechanisms, and related genes identified in *A. baumannii* smu has been provided in the Suppl Table 1C.

### 3.4 Prediction of mobile genetic elements

#### 3.4.1 Analysis of insertion sequence elements

Insertion sequence (IS) elements are the simplest transposable elements in bacteria. They increase genome flexibility and help spread antimicrobial resistance genes and virulence factors, supporting bacterial survival in harsh conditions such as antibiotic stress 29. The presence of IS elements in the genome of *A. baumannii* smu was detected using the ISEScan tool 19. The strain revealed a total of nine insertion sequence elements with a combined coverage of 18,328 bp representimg 0.46% of the genome (Table 2). The IS21 family was found to be the most abundant comprising of three elements with a total length of 10,213bp (0.26%). The IS701 family was also detected in three copies together covering 3,305bp (0.08%). Single representatives of the IS110 and IS91 families were identified contributing 2,262bp (0.06%) and 2,299bp (0.06%) respectively. Furthermore, one element of ISL3 was detected that spans 249 bp (0.01%). Overall, the IS content of the smu strain is rather low but carried in multiple families. The disproportion of IS21 and IS701 is an indication that those factors may be of greater significance in determining genome variability and adaptation in this strain.

#### 3.4.2 Identification of prophage regions

Prophages are viral genetic elements integrated into bacterial chromosome. They can switch to the lytic cycle under stress to produce new phages and often carry genes that boost bacterial survival, including antibiotic resistance and virulence 30. In this study, the prophage content of the *A. baumannii* smu strain was explored using PHASTER which identified six prophage regions varying in size, score, and gene composition (Table 3). The first region covering 6.3 kb identified between 549,591 and 555,970 bp was classified as incomplete with a score of 30. It showed similarity to PHAGE_Ralsto_PE226_NC_015297 with a GC content of 37.38%, and contained genes for transposase, hypothetical proteins, and phage-like proteins. The second region extending from 1,136,368 to 1,181,611 bp (45.2 kb) was predicted as questionable with a score of 87. It was closely related to PHAGE_Acinet_Bphi_B1251_NC_019541, showing a GC content of 39.04%. This region contained several functional genes including those coding for hypothetical proteins, phage-like proteins, coat protein, fiber protein, and tail shaft proteins. The third prophage lying between 1,336,408 and 1,377,688 bp (41.2 kb) was classified as intact with a high score of 130. It also matched PHAGE_Acinet_Bphi_B1251_NC_019541and displayed a GC content of 40.40%. Key genes included phage-like proteins, hypothetical proteins, porin-like proteins, coat proteins, tail shaft proteins, and attachment-related genes.

The fourth region located at 1,464,922-1,498,782 bp (33.8 kb) was predicted as questionable with a score of 40.This region exhibited similarity with PHAGE_Bordet_BPP_1_NC_005357 showing a GC content of 41.00%. It contained genes for coat proteins, attachment proteins, terminase, phage-like proteins, and hypothetical proteins. Similarly, the fifth region was mapped from 1,491,920 to 1,522,306 bp (30.3 kb) and was categorized as incomplete with a low score of 20. It was associated with PHAGE_Acinet_vB_AbaP_PD_6A3_NC_028684 possessing a GC content of 37.33%, and included hypothetical proteins and phage-like proteins. The sixth and largest region was found intact in the position between 2,758,541-2,813,092 bp spanning 54.5 with a score of 100. This region resembled to PHAGE_Acinet_vB_Abas_TRS1_NC_031098 with a GC content of 39.69%. This prophage carried diverse functional genes including those for hypothetical proteins, attachment proteins, phage-like proteins, terminase, coat proteins, tail shaft proteins, and porin-like proteins, as highlighted in the Table. Thus, PHASTER analysis revealed the presence of two intact prophages, two questionable regions, and two incomplete regions, indicating considerable diversity within the prophage content of the *A. baumannii* smu genome.

### 3.5 Pangenome analysis

**Figure 6:**
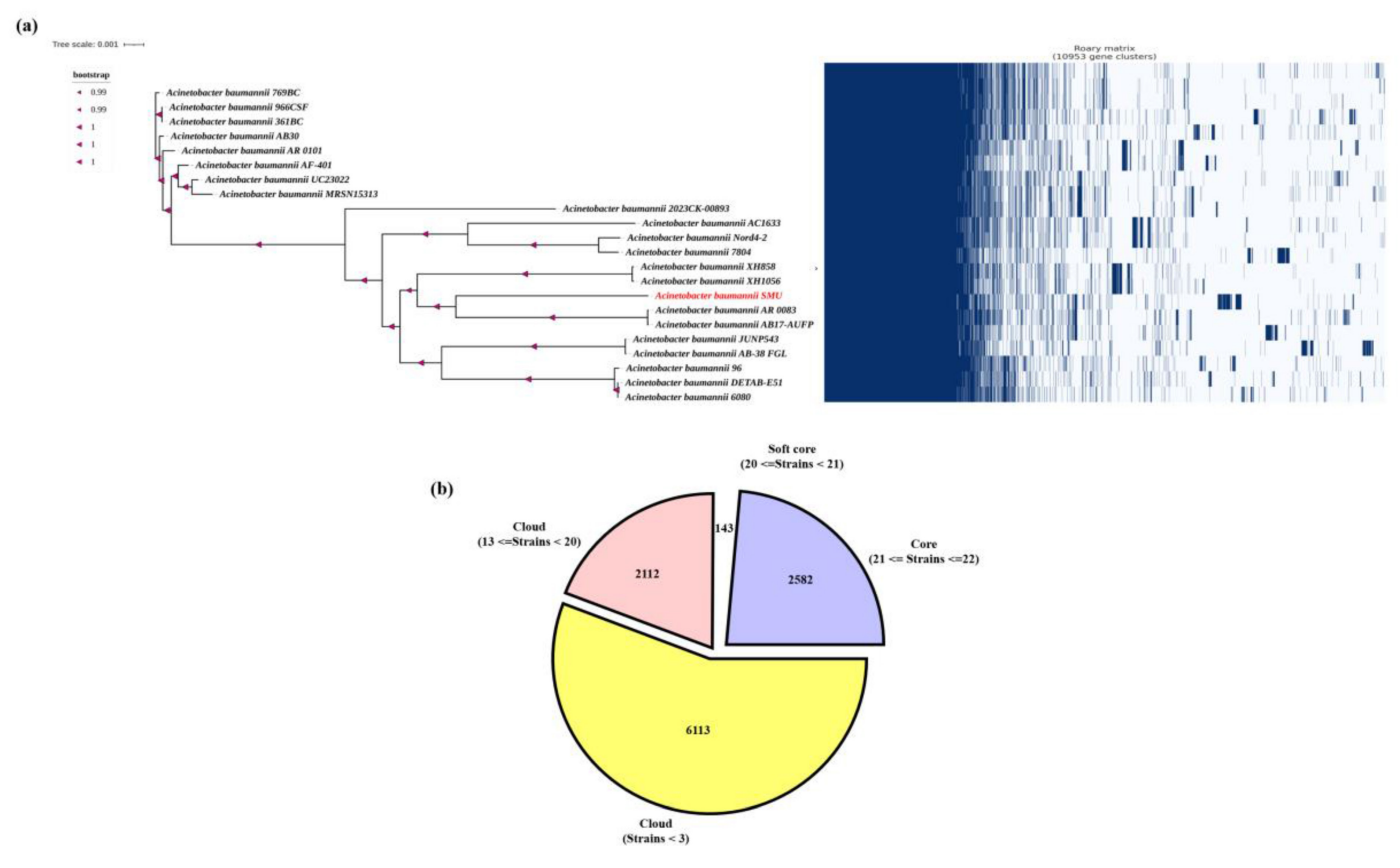
** (a)** Phylogenetic tree and presence-absence matrix of gene clusters across *A. baumannii* isolates. The maximum-likelihood phylogeny was constructed based on the core genome alignment, with bootstrap support values indicated at the nodes. The right panel shows the binary presence (blue) and absence (white) pattern of 10,993 gene clusters among all isolates. The SMU isolate (highlighted in red) forms a distinct clade and harbors a unique set of accessory genes absent in other strains. **(b)** Distribution of core, soft-core, and cloud genes within the *A. baumannii* pangenome. The pangenome comprises 10,993 total gene clusters, classified into **core genes** (present in all strains, *n* = 2,582), **soft-core genes** (present in 20-21 strains, *n* = 143), and **cloud genes** (present in <20 strains, *n* = 8,225). The predominance of cloud genes indicates a high degree of genomic diversity and accessory gene content among the analyzed isolates.

In this study, pangenome analysis was carried out to explore the genomic diversity, evolutionary relationships, and gene content variation among strains of *A. baumannii.* The pangenome comprises the total gene set of all strains within a species and is typically classified into core genes, accessory genes, and strain-specific genes 31. Initially, a maximum-likelihood phylogenetic tree was constructed based on 16S rRNA gene sequences to determine the taxonomic placement of the isolate *A. baumannii* smu. The tree revealed that the isolate clustered tightly within the *A. baumannii* clade showing a close evolutionary relationships with other *A. baumannii* strains. The bootstrap values for major nodes were found to be high (>0.99-1.0) with a branch length scale of 0.001. The pangenome of whole-genome annotation of *A. baumannii* smu revealed a total of 10,953 genes categorized into coding sequences (CDS), transfer RNAS (tRNAS), and ribosomal RNAs (rRNAs). Among these, 6113 (56.6%) were protein-coding genes, 2582 (23.9%) represented functional RNA elements including tRNAs and 2112 (19.5%) were identified as rRNA-related or hypothetical genes (Figure 6).

The comparative genomic analysis of all isolates revealed a total of 13 genes unique to the smu isolate, which were present exclusively in smu strain and completely absent from all other strains analyzed. These smu-specific genes included mmgc, *repA, tufB, fis, tri1, katB, higA-2, higB-2, cspE, higB2, esiB, ohrB,* and *ohrR*. Several of these genes are linked to stress response and regulatory functions. For example, *katB* encodes a catalase enzyme involved in oxidative stress defense, while *higA-2* and *higB-2* form a toxin-antitoxin system that may contribute to plasmid maintenance and stress tolerance. In addition, the presence of genes such as *repA* (replication initiator protein) and *fis* (factor for inversion stimulation) suggest the potential for genomic rearrangements that may be unique to the smu strain Suppl Table 2.

## 4. Discussion

Septicemia is one of the causes of morbidity and mortality within the ICU among critically ill patients. In India, a multicentric study in 35 ICUs identified a sepsis prevalence rate of 56.4% with 45 percent of the infections being due to MDR organisms 32. The *A. baumannii* has become a critical pathogen in Indian ICU with high resistance to carbapenems and other essential antibiotics 33. This trend is concerning especially in regions like Sikkim where data on sepsis and antimicrobial resistance are limited. A 2013-2018 retrospective study of AST of blood culture isolates showed a positive rate of 10.2% and highest prevalence of MDR in *Acinetobacter* species especially in infants below 1 year (20.8% of total BSIs) 34.

In line with these observations, the current study found *A. baumannii* as the most frequent MDR isolate in blood cultures in ICUs at CRH Hospital, Sikkim. The majority of the cases were a result of the NICU, which represents a potential hotspot for MDR infections among vulnerable neonates 35. In another study done by Dechen C Tsering et. al. in the year 2007-2008 in Sikkim, the most common cause of neonatal sepsis was *Staphylococcus aureus* (97%), *A. baumannii* was isolated from only one blood sample and was sensitive to many drugs 36. This clearly reflects the changing trend in the cause of septecaemia and the drug sensitivity pattern of the pathogenic bacteria.

The MDR *A. baumannii* smu genome assembly and structural annotation helps in understanding its genetic makeup. The complete 4.0 MB genome consisting of three contigs with a high sequencing depth is indicative of good quality of data, while the abundance of protein-coding genes and non-coding RNAs is indicative of the adaptability of the strain. One of the major findings of this study was the identification and categorization of ARGs. The predominance of efflux pump systems, especially, the RND family (*adeF, aeI, adeJ, adeL, adeN, adeR*) shows the key role of the multidrug resistance of this isolate which is mediated by efflux. The effux systems not only promote the development of antibiotic resistance but also increase the biofilm development and virulence, thereby making treatment complicated 37. Interestingly, the presence of multiple efflux systems and the presence of 2-lactamases including OXA-98 and aminoglycoside-modifying enzymes may be considered a peculiar combination in comparison to most of the already sequenced Indian isolates, concluding that the smu strain possesses a more sophisticated resistome architecture enabling to increase drug resistance and persistence. Similar genomic profiles with multiple resistance genes, virulence factors, and mobile genetic elements have been reported in MDR *A. baumannii* isolates, highlighting their clinical significance 38.

Prediction of virulence factors had shown that acinetobactin and heme utilization pathways of iron uptake were mostly prevalent. This kind of systems plays a pivotal role in bacterial survival in conditions of iron restriction by hosts and has been closely linked to an aimproved pathogenicity 39. Many biofilm-related genes *(Bap, Csu, pili, PNAG* exopolysaccharides) indicate that the strain is able to develop strong biofilms, which facilitates its survival and is one of the causes of bloodstream infections 40. Moreover, the expression of outer membrane protein A (OmpA) and phospholipases indicate mechanisms of host cell damage and immune evasion that could be the cause of the severity of the infections in ICU patients 41. The combination of various biofilm and iron acquisition systems predetermines the uniqueness of this isolate since most of the previous reports indicate the incomplete expression of these virulence determinants in isolated strains 42.

Accordingly, the discovery of MGEs including insertion sequences (IS21, IS701), and a variety of prophages reflects the genomic plasticity of the strain. Similar observations of abundant IS sequences and open pangenomes have been reported globally, highlighting the adaptive potential of *A. baumannii* 43. The genomic rearrangements, acquisition of resistance genes, and virulence evolution of *A. baumannii* are known to be caused by prophages and IS elements 44. In addition, IS elements such as ISAba1 have been reported to drive colistin resistance through eptA overexpression in *A. baumannii* 45. This isolate is especially dynamic due to the presence of several intact and incomplete prophages and IS elements, which might increase its adaptability rate in comparison to other regional ones. Furthermore, the pangenome analysis of the *A. baumannii* smu strain was conducted to gain more information regarding its genetic peculiarities and clinical significances. The phylogenetic positioning of the smu strain using 16S rRNA sequences revealed that the smu strain belongs to the *A. baumannii* clade which is closely related to the rest of the clinically significant members of the A. calcoaceticus-baumannii complex under good bootstrap support 46-48. The pangenome analysis of the smu isolate compared against 22 reference *A. baumannii* genomes showed that in total, the pangenome has 10,953 genes of which 56.6% are protein-coding. This genome matches those that have been previously reported in other MDR *A. baumannii* strains that generally have 13,000-15,000 genes with 50-65% of coding genes 48,49.

Interestingly, the smu strain had 13 genes that were not present in other *A. baumannii* isolates studied. These are genes like *katB* that help in oxidative stress defence, *higA-2* and *highB-2* that are part of a toxin-antitoxin in assisting the bacteria to survive in stress environment and *repA, fis* that are involved in plasmid replication and genome regulation. These genes indicate that the smu strain has come to acquire certain genetic adaptations that have increased its survival capacity to adverse clinical conditions and survive under the pressure of antibiotics, hence it has become more resilient and thus could be virulent. These results are consistent with the global reports of genetic differences within the MDR *A. baumannii* isolates 47,49,50.

## 5. Conclusions

In this study, A.* baumannii* smu has been identified as the predominant cause of septicemia in ICUs of a tertiary care hospital in Sikkim with most isolates being MDR. This represents the first in-depth genomic characterization of an MDR *A. baumannii* isolate in the region where genomic data on clinically significant pathogens are limited. The strain harbored a complex resistome including efflux pumps, β-lactamases, aminoglycoside-modifying enzymes, and key virulence factors such as biofilm formation and iron uptake systems. Pangenome analysis revealed 13 genes associated with plasmid stability, stress tolerance, and antibiotic resistance indicating local adaptation to hospital settings. These genetic features likely contribute to its persistence and the rising cases of septicemia in Sikkim. Overall, the study demonstrates the clinical challenges of treating MDR septicemia in ICUs and emphasize the need for timely antibiotic therapy and effective control. Importantly, it also provide baseline regional genomic data to support future molecular surveillance and public health strategies in Sikkim.

## Supplementary Material

Supplementary information and tables.

## Figures and Tables

**Table 1:** Summary of genome assembly and annotation statistics of the MDR *A. baumannii* smu.

Genome Feature	Value
Genome size	3,999,148 bp (4.0 Mbp)
GC %	39.16%
N50	3,981,953 bp
L50	1
Total gene count Protein-coding genes (CDS) rRNA genes tRNA genes	3,820 3,726 18 76
GenBank accession number	JBPXER000000000

**Table 2:** Summary of insertion sequence elements identified in the draft genome of *A. baumannii* smu using ISEScan.

IS Family	Number of IS Element (nIS)	% of Genome	IS bp Coverage (bp)	DNA Length
IS110	1	0.06	2262	3981953
IS21	3	0.26	10213	3981953
IS701	3	0.08	3305	3981953
IS91	1	0.06	2299	3981953
ISL3	1	0.01	249	3981953
Total	9	0.46	18328	3999148

**Table 3:** Summary of predicted prophage regions in the genome of *A. baumannii* smu identified by PHASTER.

Region	Length (kb)	Position (bp)	Type	Score	Common Phage	GC (%)	Key Genes
1	6.3	549591-555970	Incomplete	30	PHAGE_Ralsto_PE226_NC_015297(3)	37.38	*Tra, Hyp, PLP,*
2	45.2	1136368-1181611	Questionable	87	PHAGE_Acinet_Bphi_B1251_NC_019541(36)	39.04	*Hyp, PLP, Coa, Fib, Sha,*
3	41.2	1336408-1377688	Intact	130	PHAGE_Acinet_Bphi_B1251_NC_019541(13)	40.40	*PLP, Hyp, Por, Coa, Sha, Att,*
4	33.8	1464922-1498782	Questionable	70	PHAGE_Bordet_BPP_1_NC_005357 (8)	41.00	*Hyp, Coa, Att, PLP, Ter,*
5	30.3	1491920-1522306	Incomplete	20	PHAGE_Acinet_vB_AbaP_PD_6A3_NC_028684 (2)	37.33	*Hyp, PLP*
6	54.5	2758541-2813092	Intact	100	PHAGE_Acinet_vB_AbaS_TRS1_NC_031098 (26)	39.69	*Hyp, Att, PLP, Ter, Coa, Sha, Por,*

**Abbreviations:** *Tra*-Transposase, *Hy*p-Hypotheical, *PLP*-Phage-like protein, *Coa*-Coat protein, *Fib*-Fiber protein, *Sha*-Tail, shaft, *Por*-Porin-related protein, *Att*-Attachment, *Ter*-Terminase.

## Data Availability

The whole-genome shotgun sequencing data of *A. baumannii* smu have been deposited in the NCBI GenBank under accession number JBPXER000000000. The associated BioProject and BioSample accession numbers are PRJNA1263711 and SAMN48482167 respectively. Raw sequencing reads have been submitted to the NCBI Sequence Read Archive under the same BioProject. These data provide a valuable resource for future studies on antimicrobial resistance and genomic diversity of this clinically significant pathogen.
