# Supplementary material for: Draft Genome Sequence of Multidrug-Resistant Acinetobacter baumannii smu isolated from a Bloodstream Infection in Sikkim, India
Source: J Genomics. 2026 May 1;14:18–29. doi: 10.7150/jgen.130521 (PMC13143519; doi:10.7150/jgen.130521)
Supplement: Supplementary file 1 — Supplementary information and tables. [file jgenv14p0018s1.pdf]

SUPPLEMENTARY TABLES

SUPPL TABLE 1A: BUSCO statistics of *A. baumannii* smu.

**BUSCO Completeness**		***A. baumannii***
BUSCO Evaluation	Complete BUSCOs (C)	1468 (98.8%)
	Complete and single-copy BUSCOs (S)	1466 (98.7%)
	Complete and duplicated BUSCOs (D)	2 (0.1%)
	Fragmented BUSCOs (F)	9 (0.6%)
	Missing BUSCOs (M)	9 (0.6%)
	Total BUSCO groups searched (n)	1486

SUPPL TABLE 1B: Summary of antimicrobial resistance genes, families and associated resistance mechanisms in *A. baumannii* smu.

**Representative Genes**	**AMR Gene Family**	**Count**	**Drug Class**	**Resistance Mechanism**	**Functional Role**
ADC-158	ADC beta-lactamases pending classification for carbapenemase activity	1	Cephalosporin	Antibiotic inactivation	Enzyme inactivation
ANT(3")-IIa	ANT(3',',)	1	Aminoglycoside antibiotic	Antibiotic inactivation	Enzyme inactivation
LpsB	Intrinsic peptide, Antibiotic resistant, LPS	1	Peptide antibiotic	Reduced permeability to antibiotic	Reduced permeability, decreased uptake
OXA-98	OXA beta-lactamase, OXA-51-like beta-lactamase	1	Carbapenem, penicillin beta-lactam	Antibiotic inactivation	Enzymatic inactivation
Acinetobacter baumannii parC conferring resistance to fluoroquinolones	Fluoroquinolone resistant parC	1	Fluoroquinolone antibiotic	Antibiotic target alteration	Target alteration
Acinetobacter baumannii AbaF, Acinetobacter baumannii AbaQ, Acinetobacter baumannii *AmvA*	Major facilitator superfamily (MFS) antibiotic efflux pump	3	Fluoroquinolone antibiotic, macrolide antibiotic, disinfecting agents and antiseptics, phosphonic acid antibiotic	Antibiotic efflux	Efflux pump
abeM	Multidrug and toxic compound extrusion (MATE) transporter	1	Fluoroquinolone antibiotic, disinfecting agents and antiseptics	Antibiotic efflux	Efflux pump

adeF, adel, adeJ, adeK, adeL, adeN, adeR	Resistance-nodulation - cell division (RND) antibiotic efflux pump	7	Fluoroquinolone antibiotic, tetracycline antibiotic, macrolide antibiotic, diaminopyrimidine antibiotic, phenolic antibiotic	Antibiotic efflux	Efflux pump
abeS	Small multidrug resistance (SMR) antibiotic efflux pump	1	Macrolide antibiotic, aminocoumarin antibiotic	Antibiotic efflux	Efflux pump
sul2	Sulfonamide resistant sul	1	Sulfonamide antibiotic	Antibiotic target replacement	Target replacement

SUPPL TABLE 1C: Virulence factors identified in the genome sequence of *A. baumannii* smu strain.

**VF Class**	**Virulence Factors**	**Genes**
Adherence	Outer membrane protein<br>Type IV pili (Neisseri)	*OmpA*<br>*pilE*
Biofilm formation	AdeFGH efflux pump/transport autoinducer	*adeF*<br>*adeG*<br>*adeH*
	Biofilm-associated protein	*bap*
	Csu pili	*csuA/B*<br>*csuA*<br>*csuB*<br>*csuC*<br>*csuD*<br>*csuE*
	PNAG (Polysaccharide poly-N-acetylglucosamine	*pgaA*<br>*pgaB*<br>*pgaC*<br>*pgaD*
Enzyme	Phospholipase C<br>Phospholipase D	*plc*<br>*plcD*
Immune evasion	Capsule<br>Lipopolysaccharide	-<br>*IpxB*<br>*IpxC*<br>*IpxD*<br>*IpxL*<br>*IpxM*
Iron uptake	Acinetobactin	*barA*<br>*barb*<br>*basA*<br>*basB*<br>*basC*<br>*basD*<br>*basF*<br>*basG*<br>*bash*<br>*basI*<br>*basJ*<br>*bauA*<br>*bauB*<br>*bauC*<br>*bauD*<br>*bauE*<br>*bauF*<br>*entE*
	Heme utilization	*hemO*
Regulation	Quorum sensing	*abaI*

		abaR
	Two-component system	*bfmR*<br>*bfmS*
Serum resistance	PbpG	*pbpG*

SUPPL TABLE 1D

GeneID	361BC	6080	769BC	7804	96	966CSF	AB-38_FGL	AB17-AUFF
astB	1	1	1	1	1	1	1	1
kgtP	1	1	1	1	1	1	1	1
rho	1	1	1	1	1	1	1	1
mmgC	1	1	1	1	1	1	1	1
pqqC	1	1	1	1	1	1	1	1
tolB	1	1	1	1	1	1	1	1
ansB	1	1	1	1	1	1	1	1
trmR	1	1	1	1	1	1	1	1
yajR	1	1	1	1	1	1	1	1
nudC	1	1	1	1	1	1	1	1
ftsI	1	1	1	1	1	1	1	1
mreB	1	1	1	1	1	1	1	1
gltB	1	1	1	1	1	1	1	1
feoB	1	1	1	1	1	1	1	1
hisA	1	1	1	1	1	1	1	1
ruvA	1	1	1	1	1	1	1	1
atpH	1	1	1	1	1	1	1	1
pheA	1	1	1	1	1	1	1	1
ssuD	1	1	1	1	1	1	1	1
yihG	1	1	1	1	1	1	1	1
linX	1	1	1	1	1	1	1	1
trpD	1	1	1	1	1	1	1	1
nadX	1	1	1	1	1	1	1	1
gltP	1	1	1	1	1	1	1	1
nfsB	1	1	1	1	1	1	1	1
fadA	1	1	1	1	1	1	1	1
uvrC	1	1	1	1	1	1	1	1
mnmA	1	1	1	1	1	1	1	1
gapN	1	1	1	1	1	1	1	1
aatA	1	1	1	1	1	1	1	1
nadA	1	1	1	1	1	1	1	1
mymA	1	1	1	1	1	1	1	1
fusA	1	1	1	1	1	1	1	1
sfnG	1	1	1	1	1	1	1	1
clsC	1	1	1	1	1	1	1	1
pntA	1	1	1	1	1	1	1	1
ohrR	1	1	1	1	1	1	1	1
scpB	1	1	1	1	1	1	1	1
trpG	1	1	1	1	1	1	1	1
etfB	1	1	1	1	1	1	1	1
kdsA	1	1	1	1	1	1	1	1
ybjJ	1	1	1	1	1	1	1	1
clpV1	1	1	1	1	1	1	1	1
pcaH	1	1	1	1	1	1	1	1
leuA	1	1	1	1	1	1	1	1
panD	1	1	1	1	1	1	1	1
groL	1	1	1	1	1	1	1	1
dmlR	1	1	1	1	1	1	1	1

glnD	1	1	1	1	1	1	1	1
htpG	1	1	1	1	1	1	1	1
tsaQ1	1	1	1	1	1	1	1	1
thiG	1	1	1	1	1	1	1	1
sucB	1	1	1	1	1	1	1	1
yciK	1	1	1	1	1	1	1	1
accC	1	1	1	1	1	1	1	1
argA	1	1	1	1	1	1	1	1
catA	1	1	1	1	1	1	1	1
tal	1	1	1	1	1	1	1	1
sdhC	1	1	1	1	1	1	1	1
radA	1	1	1	1	1	1	1	1
glpK	1	1	1	1	1	1	1	1
edd	1	1	1	1	1	1	1	1
nuoL	1	1	1	1	1	1	1	1
lon	1	1	1	1	1	1	1	1
sstT	1	1	1	1	1	1	1	1
hmp	1	1	1	1	1	1	1	1
recR	1	1	1	1	1	1	1	1
yhfA	1	1	1	1	1	1	1	1
cyoA	1	1	1	1	1	1	1	1
fabR	1	1	1	1	1	1	1	1
tig	1	1	1	1	1	1	1	1
pta	1	1	1	1	1	1	1	1
pyrC'	1	1	1	1	1	1	1	1
cysH	1	1	1	1	1	1	1	1
gntT	1	1	1	1	1	1	1	1
dapA	1	1	1	1	1	1	1	1
betA	1	1	1	1	1	1	1	1
lolB	1	1	1	1	1	1	1	1
tas	1	1	1	1	1	1	1	1
thrH	1	1	1	1	1	1	1	1
hmrR	1	1	1	1	1	1	1	1
gltK	1	1	1	1	1	1	1	1
corC	1	1	1	1	1	1	1	1
zipA	1	1	1	1	1	1	1	1
ribE	1	1	1	1	1	1	1	1
fumC	1	1	1	1	1	1	1	1
rpsG	1	1	1	1	1	1	1	1
caiT	1	1	1	1	1	1	1	1
lrp	1	1	1	1	1	1	1	1
rarD	1	1	1	1	1	1	1	1
lldP	1	1	1	1	1	1	1	1
gltJ	1	1	1	1	1	1	1	1
parB	1	1	1	1	1	1	1	1
menH	1	1	1	1	1	1	1	1
grpE	1	1	1	1	1	1	1	1
exbD	1	1	1	1	1	1	1	1
pyrD	1	1	1	1	1	1	1	1
ubiG	1	1	1	1	1	1	1	1

caiD	1	1	1	1	1	1	1	1
pgaC	1	1	1	1	1	1	1	1
metK	1	1	1	1	1	1	1	1
cydB	1	1	1	1	1	1	1	1
pilQ	1	1	1	1	1	1	1	1
fdx	1	1	1	1	1	1	1	1
ftsB	1	1	1	1	1	1	1	1
cysW	1	1	1	1	1	1	1	1
yfeX	1	1	1	1	1	1	1	1
katG	1	1	1	1	1	1	1	1
sspA	1	1	1	1	1	1	1	1
hemC	1	1	1	1	1	1	1	1
frmA	1	1	1	1	1	1	1	1
purU	1	1	1	1	1	1	1	1
glnK	1	1	1	1	1	1	1	1
acoB	1	1	1	1	1	1	1	1
clpA	1	1	1	1	1	1	1	1
nrtP	1	1	1	1	1	1	1	1
spoT	1	1	1	1	1	1	1	1
mscS	1	1	1	1	1	1	1	1
rhtC	1	1	1	1	1	1	1	1
pheS	1	1	1	1	1	1	1	1
rpsJ	1	1	1	1	1	1	1	1
uvrA	1	1	1	1	1	1	1	1
ybdG	1	1	1	1	1	1	1	1
tolQ	1	1	1	1	1	1	1	1
hda	1	1	1	1	1	1	1	1
purM	1	1	1	1	1	1	1	1
relA	1	1	1	1	1	1	1	1
rlmB	1	1	1	1	1	1	1	1
rpsL	1	1	1	1	1	1	1	1
yedI	1	1	1	1	1	1	1	1
lip	1	1	1	1	1	1	1	1
alaA	1	1	1	1	1	1	1	1
cinA	1	1	1	1	1	1	1	1
rpoN	1	1	1	1	1	1	1	1
rplF	1	1	1	1	1	1	1	1
dadA	1	1	1	1	1	1	1	1
murC	1	1	1	1	1	1	1	1
fabB	1	1	1	1	1	1	1	1
kefC	1	1	1	1	1	1	1	1
mmpI	1	1	1	1	1	1	1	1
hdfR	1	1	1	1	1	1	1	1
phoB	1	1	1	1	1	1	1	1
rpe	1	1	1	1	1	1	1	1
glsA	1	1	1	1	1	1	1	1
zntR	1	1	1	1	1	1	1	1
baiA	1	1	1	1	1	1	1	1
citN	1	1	1	1	1	1	1	1
rubB	1	1	1	1	1	1	1	1

rocC	1	1	1	1	1	1	1	1
ftsW	1	1	1	1	1	1	1	1
queA	1	1	1	1	1	1	1	1
nuoG	1	1	1	1	1	1	1	1
lysA	1	1	1	1	1	1	1	1
mutS	1	1	1	1	1	1	1	1
cysN	1	1	1	1	1	1	1	1
rhtA	1	1	1	1	1	1	1	1
rutR	1	1	1	1	1	1	1	1
sasA	1	1	1	1	1	1	1	1
dxr	1	1	1	1	1	1	1	1
iucD	1	1	1	1	1	1	1	1
rhaS	1	1	1	1	1	1	1	1
valS	1	1	1	1	1	1	1	1
znuC	1	1	1	1	1	1	1	1
tolC	1	1	1	1	1	1	1	1
aroB	1	1	1	1	1	1	1	1
znuB	1	1	1	1	1	1	1	1
prpC	1	1	1	1	1	1	1	1
prlC	1	1	1	1	1	1	1	1
acsA	1	1	1	1	1	1	1	1
sbp	1	1	1	1	1	1	1	1
catC	1	1	1	1	1	1	1	1
xecA1	1	1	1	1	1	1	1	1
fldP	1	1	1	1	1	1	1	1
alr1	1	1	1	1	1	1	1	1
tatA	1	1	1	1	1	1	1	1
ppiC	1	1	1	1	1	1	1	1
macB	1	1	1	1	1	1	1	1
hutI	1	1	1	1	1	1	1	1
pcaF	1	1	1	1	1	1	1	1
lipA	1	1	1	1	1	1	1	1
sadH	1	1	1	1	1	1	1	1
clsA	1	1	1	1	1	1	1	1
gltC	1	1	1	1	1	1	1	1
nimR	1	1	1	1	1	1	1	1
nit1	1	1	1	1	1	1	1	1
fkpA	1	1	1	1	1	1	1	1
ribB	1	1	1	1	1	1	1	1
soj	1	1	1	1	1	1	1	1
nlpD	1	1	1	1	1	1	1	1
antC	1	1	1	1	1	1	1	1
purN	1	1	1	1	1	1	1	1
norG	1	1	1	1	1	1	1	1
pcaU	1	1	1	1	1	1	1	1
murI	1	1	1	1	1	1	1	1
lexA	1	1	1	1	1	1	1	1
argJ	1	1	1	1	1	1	1	1
sutR	1	1	1	1	1	1	1	1
murF	1	1	1	1	1	1	1	1

prtR	1	1	1	1	1	1	1	1
hsrA	1	1	1	1	1	1	1	1
proS	1	1	1	1	1	1	1	1
btuE	1	1	1	1	1	1	1	1
dctA	1	1	1	1	1	1	1	1
hemE	1	1	1	1	1	1	1	1
teaD	1	1	1	1	1	1	1	1
mrpF	1	1	1	1	1	1	1	1
murB	1	1	1	1	1	1	1	1
mutM	1	1	1	1	1	1	1	1
metZ	1	1	1	1	1	1	1	1
mneA	1	1	1	1	1	1	1	1
gabD	1	1	1	1	1	1	1	1
glaR	1	1	1	1	1	1	1	1
prpF	1	1	1	1	1	1	1	1
htpX	1	1	1	1	1	1	1	1
glnH	1	1	1	1	1	1	1	1
bioA	1	1	1	1	1	1	1	1
rpsP	1	1	1	1	1	1	1	1
yheS	1	1	1	1	1	1	1	1
rpmE	1	1	1	1	1	1	1	1
thlA	1	1	1	1	1	1	1	1
tolR	1	1	1	1	1	1	1	1
hisQ	1	1	1	1	1	1	1	1
hisD	1	1	1	1	1	1	1	1
mrkD	1	1	1	1	1	1	1	1
proC	1	1	1	1	1	1	1	1
dhaT	1	1	1	1	1	1	1	1
ybiV	1	1	1	1	1	1	1	1
ppsC	1	1	1	1	1	1	1	1
acoA	1	1	1	1	1	1	1	1
lepB	1	1	1	1	1	1	1	1
ispU	1	1	1	1	1	1	1	1
yahB	1	1	1	1	1	1	1	1
trpE	1	1	1	1	1	1	1	1
rlmA	1	1	1	1	1	1	1	1
ispD	1	1	1	1	1	1	1	1
cbdA	1	1	1	1	1	1	1	1
lolA	1	1	1	1	1	1	1	1
aprA	1	1	1	1	1	1	1	1
nagR	1	1	1	1	1	1	1	1
allS	1	1	1	1	1	1	1	1
nagZ	1	1	1	1	1	1	1	1
rhtB	1	1	1	1	1	1	1	1
glyS	1	1	1	1	1	1	1	1
cysQ	1	1	1	1	1	1	1	1
hcaB	1	1	1	1	1	1	1	1
macA	1	1	1	1	1	1	1	1
xerC	1	1	1	1	1	1	1	1
sbnF	1	1	1	1	1	1	1	1

smc	1	1	1	1	1	1	1	1
hemA	1	1	1	1	1	1	1	1
dedA	1	1	1	1	1	1	1	1
folE	1	1	1	1	1	1	1	1
rplB	1	1	1	1	1	1	1	1
yjhB	1	1	1	1	1	1	1	1
pucK	1	1	1	1	1	1	1	1
prfC	1	1	1	1	1	1	1	1
truA	1	1	1	1	1	1	1	1
guaD	1	1	1	1	1	1	1	1
smpB	1	1	1	1	1	1	1	1
ssuC	1	1	1	1	1	1	1	1
plaP	1	1	1	1	1	1	1	1
apaH	1	1	1	1	1	1	1	1
lpxD	1	1	1	1	1	1	1	1
glxR	1	1	1	1	1	1	1	1
pcaJ	1	1	1	1	1	1	1	1
ahr	1	1	1	1	1	1	1	1
ttgC	1	1	1	1	1	1	1	1
paaE	1	1	1	1	1	1	1	1
outO	1	1	1	1	1	1	1	1
soxC	1	1	1	1	1	1	1	1
folC	1	1	1	1	1	1	1	1
rsxB	1	1	1	1	1	1	1	1
hisP	1	1	1	1	1	1	1	1
deaD	1	1	1	1	1	1	1	1
feaR	1	1	1	1	1	1	1	1
proY	1	1	1	1	1	1	1	1
rodZ	1	1	1	1	1	1	1	1
uao	1	1	1	1	1	1	1	1
aes	1	1	1	1	1	1	1	1
mmsA	1	1	1	1	1	1	1	1
argC	1	1	1	1	1	1	1	1
yclP	1	1	1	1	1	1	1	1
gabP	1	1	1	1	1	1	1	1
recC	1	1	1	1	1	1	1	1
phaC	1	1	1	1	1	1	1	1
lnt	1	1	1	1	1	1	1	1
metG	1	1	1	1	1	1	1	1
vibB	1	1	1	1	1	1	1	1
recJ	1	1	1	1	1	1	1	1
selO	1	1	1	1	1	1	1	1
lifO	1	1	1	1	1	1	1	1
dsbC	1	1	1	1	1	1	1	1
yofA	1	1	1	1	1	1	1	1
birA	1	1	1	1	1	1	1	1
pxpA	1	1	1	1	1	1	1	1
nanT	1	1	1	1	1	1	1	1
xpt	1	1	1	1	1	1	1	1
bamD	1	1	1	1	1	1	1	1

groS	1	1	1	1	1	1	1	1
ycfH	1	1	1	1	1	1	1	1
bcr	1	1	1	1	1	1	1	1
folK	1	1	1	1	1	1	1	1
rpmE2	1	1	1	1	1	1	1	1
qseC	1	1	1	1	1	1	1	1
paaG	1	1	1	1	1	1	1	1
cidB	1	1	1	1	1	1	1	1
tdeA	1	1	1	1	1	1	1	1
dauR	1	1	1	1	1	1	1	1
lipB	1	1	1	1	1	1	1	1
thiL	1	1	1	1	1	1	1	1
ndh	1	1	1	1	1	1	1	1
gltR	1	1	1	1	1	1	1	1
rdmC	1	1	1	1	1	1	1	1
ybdL	1	1	1	1	1	1	1	1
ubiK	1	1	1	1	1	1	1	1
nsrR	1	1	1	1	1	1	1	1
pcaK	1	1	1	1	1	1	1	1
mmsB	1	1	1	1	1	1	1	1
bauC	1	1	1	1	1	1	1	1
ydeP	1	1	1	1	1	1	1	1
amiE	1	1	1	1	1	1	1	1
pabC	1	1	1	1	1	1	1	1
acdA	1	1	1	1	1	1	1	1
ssuB	1	1	1	1	1	1	1	1
ydgI	1	1	1	1	1	1	1	1
rpsO	1	1	1	1	1	1	1	1
rpmA	1	1	1	1	1	1	1	1
gph	1	1	1	1	1	1	1	1
yqgF	1	1	1	1	1	1	1	1
mutY	1	1	1	1	1	1	1	1
yejB	1	1	1	1	1	1	1	1
antB	1	1	1	1	1	1	1	1
sacA	1	1	1	1	1	1	1	1
srpA	1	1	1	1	1	1	1	1
rpmF	1	1	1	1	1	1	1	1
glmS	1	1	1	1	1	1	1	1
accA1	1	1	1	1	1	1	1	1
catB	1	1	1	1	1	1	1	1
yaaA	1	1	1	1	1	1	1	1
fmt	1	1	1	1	1	1	1	1
glnQ	1	1	1	1	1	1	1	1
mpl	1	1	1	1	1	1	1	1
pobR	1	1	1	1	1	1	1	1
gstB	1	1	1	1	1	1	1	1
phnR	1	1	1	1	1	1	1	1
mtnN	1	1	1	1	1	1	1	1
madA	1	1	1	1	1	1	1	1
glnP	1	1	1	1	1	1	1	1

algC	1	1	1	1	1	1	1	1
leuO	1	1	1	1	1	1	1	1
mshB	1	1	1	1	1	1	1	1
tmk	1	1	1	1	1	1	1	1
bauA	1	1	1	1	1	1	1	1
bepC	1	1	1	1	1	1	1	1
rsmJ	1	1	1	1	1	1	1	1
hemH	1	1	1	1	1	1	1	1
msbA	1	1	1	1	1	1	1	1
styD	1	1	1	1	1	1	1	1
epmA	1	1	1	1	1	1	1	1
accA	1	1	1	1	1	1	1	1
cbpA	1	1	1	1	1	1	1	1
hisC	1	1	1	1	1	1	1	1
serB1	1	1	1	1	1	1	1	1
mupP	1	1	1	1	1	1	1	1
fadD3	1	1	1	1	1	1	1	1
pheT	1	1	1	1	1	1	1	1
atpD1	1	1	1	1	1	1	1	1
mnhE1	1	1	1	1	1	1	1	1
fadJ	1	1	1	1	1	1	1	1
bamA	1	1	1	1	1	1	1	1
thiD	1	1	1	1	1	1	1	1
lpxL	1	1	1	1	1	1	1	1
hpxO	1	1	1	1	1	1	1	1
hutG	1	1	1	1	1	1	1	1
ydcO	1	1	1	1	1	1	1	1
pdxJ	1	1	1	1	1	1	1	1
rnfH	1	1	1	1	1	1	1	1
fpr	1	1	1	1	1	1	1	1
liuE	1	1	1	1	1	1	1	1
kdsB	1	1	1	1	1	1	1	1
fatB	1	1	1	1	1	1	1	1
pnuC	1	1	1	1	1	1	1	1
thiC	1	1	1	1	1	1	1	1
srpG	1	1	1	1	1	1	1	1
group	1	1	1	1	1	1	1	1
astD	1	1	1	1	1	1	1	1
tauD	1	1	1	1	1	1	1	1
ttuB	1	1	1	1	1	1	1	1
pncB2	1	1	1	1	1	1	1	1
glpQ	1	1	1	1	1	1	1	1
pobA	1	1	1	1	1	1	1	1
thiO	1	1	1	1	1	1	1	1
iolS	1	1	1	1	1	1	1	1
purT	1	1	1	1	1	1	1	1
metN	1	1	1	1	1	1	1	1
menF	1	1	1	1	1	1	1	1
yceJ	1	1	1	1	1	1	1	1
hcaR	1	1	1	1	1	1	1	1

pyrF	1	1	1	1	1	1	1	1
epd	1	1	1	1	1	1	1	1
paaJ	1	1	1	1	1	1	1	1
rutF	1	1	1	1	1	1	1	1
ybaN	1	1	1	1	1	1	1	1
rsmI	1	1	1	1	1	1	1	1
ilvB	1	1	1	1	1	1	1	1
rnhB	1	1	1	1	1	1	1	1
ydeN	1	1	1	1	1	1	1	1
yccS	1	1	1	1	1	1	1	1
rpmC	1	1	1	1	1	1	1	1
dsbD	1	1	1	1	1	1	1	1
emrA	1	1	1	1	1	1	1	1
virS	1	1	1	1	1	1	1	1
paaA	1	1	1	1	1	1	1	1
dmoA	1	1	1	1	1	1	1	1
acr3	1	1	1	1	1	1	1	1
nagL	1	1	1	1	1	1	1	1
lolE	1	1	1	1	1	1	1	1
fsr	1	1	1	1	1	1	1	1
adh1	1	1	1	1	1	1	1	1
dld	1	1	1	1	1	1	1	1
moaC	1	1	1	1	1	1	1	1
bolA	1	1	1	1	1	1	1	1
mmuM	1	1	1	1	1	1	1	1
budC	1	1	1	1	1	1	1	1
truB	1	1	1	1	1	1	1	1
leuD	1	1	1	1	1	1	1	1
gloB	1	1	1	1	1	1	1	1
rcsC	1	1	1	1	1	1	1	1
hxlR	1	1	1	1	1	1	1	1
cybC	1	1	1	1	1	1	1	1
rsmE	1	1	1	1	1	1	1	1
mgtA	1	1	1	1	1	1	1	1
denD	1	1	1	1	1	1	1	1
gcvH	1	1	1	1	1	1	1	1
ureF	1	1	1	1	1	1	1	1
ygiC	1	1	1	1	1	1	1	1
mazG	1	1	1	1	1	1	1	1
paaF	1	1	1	1	1	1	1	1
azoR1	1	1	1	1	1	1	1	1
rimI	1	1	1	1	1	1	1	1
tdh	1	1	1	1	1	1	1	1
queH	1	1	1	1	1	1	1	1
ureD	1	1	1	1	1	1	1	1
alc	1	1	1	1	1	1	1	1
ansA	1	1	1	1	1	1	1	1
ureE	1	1	1	1	1	1	1	1
modB	1	1	1	1	1	1	1	1
secG	1	1	1	1	1	1	1	1

pucI	1	1	1	1	1	1	1	1
thiE	1	1	1	1	1	1	1	1
engB	1	1	1	1	1	1	1	1
hemF	1	1	1	1	1	1	1	1
bacC	1	1	1	1	1	1	1	1
pqqB	1	1	1	1	1	1	1	1
acm	1	1	1	1	1	1	1	1
minC	1	1	1	1	1	1	1	1
pcaI	1	1	1	1	1	1	1	1
tauA	1	1	1	1	1	1	1	1
ygiD	1	1	1	1	1	1	1	1
ywrO	1	1	1	1	1	1	1	1
aaeB	1	1	1	1	1	1	1	1
leuC	1	1	1	1	1	1	1	1
acnA	1	1	1	1	1	1	1	1
coaX	1	1	1	1	1	1	1	1
tam	1	1	1	1	1	1	1	1
ureB	1	1	1	1	1	1	1	1
ubiI	1	1	1	1	1	1	1	1
benC	1	1	1	1	1	1	1	1
nimT	1	1	1	1	1	1	1	1
sufS	1	1	1	1	1	1	1	1
gabR	1	1	1	1	1	1	1	1
paiB	1	1	1	1	1	1	1	1
lpxK	1	1	1	1	1	1	1	1
farB	1	1	1	1	1	1	1	1
cobT	1	1	1	1	1	1	1	1
ycgJ	1	1	1	1	1	1	1	1
norR	1	1	1	1	1	1	1	1
blh	1	1	1	1	1	1	1	1
quiB	1	1	1	1	1	1	1	1
pcaB	1	1	1	1	1	1	1	1
rsuA	1	1	1	1	1	1	1	1
hchA	1	1	1	1	1	1	1	1
pgaA	1	1	1	1	1	1	1	1
purH	1	1	1	1	1	1	1	1
pucD	1	1	1	1	1	1	1	1
yddE	1	1	1	1	1	1	1	1
pld	1	1	1	1	1	1	1	1
ampG	1	1	1	1	1	1	1	1
kefF	1	1	1	1	1	1	1	1
yhbO	1	1	1	1	1	1	1	1
mdtI	1	1	1	1	1	1	1	1
COQ5	1	1	1	1	1	1	1	1
racE	1	1	1	1	1	1	1	1
oatA	1	1	1	1	1	1	1	1
yliI	1	1	1	1	1	1	1	1
yfcG	1	1	1	1	1	1	1	1
ampD	1	1	1	1	1	1	1	1
atpF	1	1	1	1	1	1	1	1

cueR	1	1	1	1	1	1	1	1
murD	1	1	1	1	1	1	1	1
ephD	1	1	1	1	1	1	1	1
rep	1	1	1	1	1	1	1	1
recB	1	1	1	1	1	1	1	1
recD	1	1	1	1	1	1	1	1
hemW	1	1	1	1	1	1	1	1
ptsP	1	1	1	1	1	1	1	1
leuE	1	1	1	1	1	1	1	1
pcrA	1	1	1	1	1	1	1	1
ybaL	1	1	1	1	1	1	1	1
tatB	1	1	1	1	1	1	1	1
crt	1	1	1	1	1	1	1	1
rlmD	1	1	1	1	1	1	1	1
rapZ	1	1	1	1	1	1	1	1
yqaA	1	1	1	1	1	1	1	1
tsaB	1	1	1	1	1	1	1	1
fghA	1	1	1	1	1	1	1	1
ogt	1	1	1	1	1	1	1	1
yfiH	1	1	1	1	1	1	1	1
yggS	1	1	1	1	1	1	1	1
gudP	1	1	1	1	1	1	1	1
def	1	1	1	1	1	1	1	1
mro	1	1	1	1	1	1	1	1
dsdA	1	1	1	1	1	1	1	1
pfeA	1	1	1	1	1	1	1	1
ytrE	1	1	1	1	1	1	1	1
ygfA	1	1	1	1	1	1	1	1
zitB	1	1	1	1	1	1	1	1
phnO	1	1	1	1	1	1	1	1
actP	1	1	1	1	1	1	1	1
xseA	1	1	1	1	1	1	1	1
lcdH	1	1	1	1	1	1	1	1
paaH	1	1	1	1	1	1	1	1
yghU	1	1	1	1	1	1	1	1
mdcC	1	1	1	1	1	1	1	1
arsR2	1	1	1	1	1	1	1	1
cmr	1	1	1	1	1	1	1	1
smf-1	1	1	1	1	1	1	1	1
cobB	1	1	1	1	1	1	1	1
tadA	1	1	1	1	1	1	1	1
iutA	1	1	1	1	1	1	1	1
srpC	1	1	1	1	1	1	1	1
tauB	1	1	1	1	1	1	1	1
bdhA	1	1	1	1	1	1	1	1
C1-hpah	1	1	1	1	1	1	1	1
mopA	1	1	1	1	1	1	1	1
pqqD	1	1	1	1	1	1	1	1
pqqE	1	1	1	1	1	1	1	1
surE	1	1	1	1	1	1	1	1

araC	1	1	1	1	1	1	1	1
kynU	1	1	1	1	1	1	1	1
lpxA	1	1	1	1	1	1	1	1
mobA	1	1	1	1	1	1	1	1
plcN	1	1	1	1	1	1	1	1
thiM	1	1	1	1	1	1	1	1
eutB	1	1	1	1	1	1	1	1
glnS	1	1	1	1	1	1	1	1
acnB	1	1	1	1	1	1	1	1
prmC	1	1	1	1	1	1	1	1
fgd	1	1	1	1	1	1	1	1
pepN	1	1	1	1	1	1	1	1
btuB	1	1	1	1	1	1	1	1
lagD	1	1	1	1	1	1	1	1
entE	1	1	1	1	1	1	1	1
phrB	1	1	1	1	1	1	1	1
nmoA	1	1	1	1	1	1	1	1
recO	1	1	1	1	1	1	1	1
degQ	1	1	1	1	1	1	1	1
cpdA	1	1	1	1	1	1	1	1
acrA	1	1	1	1	1	1	1	1
cmpR	1	1	1	1	1	1	1	1
cobO	1	1	1	1	1	1	1	1
aidA	1	1	1	1	1	1	1	1
pdhD	1	1	1	1	1	1	1	1
metC	1	1	1	1	1	1	1	1
craA	1	1	1	1	1	1	1	1
bfr	1	1	1	1	1	1	1	1
aroK	1	1	1	1	1	1	1	1
ynfM	1	1	1	1	1	1	1	1
puuE	1	1	1	1	1	1	1	1
ydgC	1	1	1	1	1	1	1	1
lrpC	1	1	1	1	1	1	1	1
cntO	1	1	1	1	1	1	1	1
lipR	1	1	1	1	1	1	1	1
allA	1	1	1	1	1	1	1	1
tag	1	1	1	1	1	1	1	1
ybjG	1	1	1	1	1	1	1	1
ndmA	1	1	1	1	1	1	1	1
katE	1	1	1	1	1	1	1	1
tcdA	1	1	1	1	1	1	1	1
atzF	1	1	1	1	1	1	1	1
ssuA	1	1	1	1	1	1	1	1
desA3	1	1	1	1	1	1	1	1
tyrB	1	1	1	1	1	1	1	1
rluA	1	1	1	1	1	1	1	1
dadX	1	1	1	1	1	1	1	1
yqjF	1	1	1	1	1	1	1	1
ant1	1	1	1	1	1	1	1	1
yqjI	1	1	1	1	1	1	1	1

comR	1	1	1	1	1	1	1	1
betI	1	1	1	1	1	1	1	1
ribD	1	1	1	1	1	1	1	1
gluQ	1	1	1	1	1	1	1	1
phoU	1	1	1	1	1	1	1	1
ubiB	1	1	1	1	1	1	1	1
ubiJ	1	1	1	1	1	1	1	1
trmB	1	1	1	1	1	1	1	1
crcB	1	1	1	1	1	1	1	1
sfnC	1	1	1	1	1	1	1	1
mshA	1	1	1	1	1	1	1	1
uup	1	1	1	1	1	1	1	1
calB	1	1	1	1	1	1	1	1
msrA	1	1	1	1	1	1	1	1
rdgB	1	1	1	1	1	1	1	1
eda	1	1	1	1	1	1	1	1
lapB	1	1	1	1	1	1	1	1
hisS	1	1	1	1	1	1	1	1
dusA	1	1	1	1	1	1	1	1
pntAA	1	1	1	1	1	1	1	1
cysM	1	1	1	1	1	1	1	1
infC	1	1	1	1	1	1	1	1
tilS	1	1	1	1	1	1	1	1
nudL	1	1	1	1	1	1	1	1
yrdA	1	1	1	1	1	1	1	1
gcvA	1	1	1	1	1	1	1	1
uctC	1	1	1	1	1	1	1	1
ifcA	1	1	1	1	1	1	1	1
ais	1	1	1	1	1	1	1	1
ndhC	1	1	1	1	1	1	1	1
bioF	1	1	1	1	1	1	1	1
bioD1	1	1	1	1	1	1	1	1
pth	1	1	1	1	1	1	1	1
rplY	1	1	1	1	1	1	1	1
nlhH	1	1	1	1	1	1	1	1
hemB	1	1	1	1	1	1	1	1
ggt	1	1	1	1	1	1	1	1
yeiR	1	1	1	1	1	1	1	1
pgaB	1	1	1	1	1	1	1	1
catD	1	1	1	1	1	1	1	1
proP	1	1	1	1	1	1	1	1
egtB	1	1	1	1	1	1	1	1
tesA	1	1	1	1	1	1	1	1
nlpE	1	1	1	1	1	1	1	1
gdhA	1	1	1	1	1	1	1	1
pepP	1	1	1	1	1	1	1	1
ubiH	1	1	1	1	1	1	1	1
argO	1	1	1	1	1	1	1	1
garK	1	1	1	1	1	1	1	1
hisZ	1	1	1	1	1	1	1	1

vfr	1	1	1	1	1	1	1	1
cbdB	1	1	1	1	1	1	1	1
benM	1	1	1	1	1	1	1	1
kdsC	1	1	1	1	1	1	1	1
lpxP	1	1	1	1	1	1	1	1
ddl	1	1	1	1	1	1	1	1
sdaA	1	1	1	1	1	1	1	1
paaZ	1	1	1	1	1	1	1	1
paaC	1	1	1	1	1	1	1	1
paaD	1	1	1	1	1	1	1	1
phnX	1	1	1	1	1	1	1	1
carC	1	1	1	1	1	1	1	1
dpgD	1	1	1	1	1	1	1	1
kmo	1	1	1	1	1	1	1	1
yjdJ	1	1	1	1	1	1	1	1
glnM	1	1	1	1	1	1	1	1
cidA	1	1	1	1	1	1	1	1
nfdA	1	1	1	1	1	1	1	1
dapL	1	1	1	1	1	1	1	1
ygaZ	1	1	1	1	1	1	1	1
metQ	1	1	1	1	1	1	1	1
htrE	1	1	1	1	1	1	1	1
yadV	1	1	1	1	1	1	1	1
pitA	1	1	1	1	1	1	1	1
yceF	1	1	1	1	1	1	1	1
surA	1	1	1	1	1	1	1	1
lptD	1	1	1	1	1	1	1	1
murU	1	1	1	1	1	1	1	1
rsmG	1	1	1	1	1	1	1	1
hbpA	1	1	1	1	1	1	1	1
gsiC	1	1	1	1	1	1	1	1
ybaK	1	1	1	1	1	1	1	1
mtlD	1	1	1	1	1	1	1	1
cobP	1	1	1	1	1	1	1	1
aroF	1	1	1	1	1	1	1	1
lpxB	1	1	1	1	1	1	1	1
aspA	1	1	1	1	1	1	1	1
pcpR	1	1	1	1	1	1	1	1
dhbA	1	1	1	1	1	1	1	1
yhcR	1	1	1	1	1	1	1	1
emrB	1	1	1	1	1	1	1	1
bbsG	1	1	1	1	1	1	1	1
soxA	1	1	1	1	1	1	1	1
lacC	1	1	1	1	1	1	1	1
decR	1	1	1	1	1	1	1	1
hslR	1	1	1	1	1	1	1	1
rimO	1	1	1	1	1	1	1	1
moaE1	1	1	1	1	1	1	1	1
norW	1	1	1	1	1	1	1	1
cmpC	1	1	1	1	1	1	1	1

umuC	1	1	1	1	1	1	1	1
prs2	1	1	1	1	1	1	1	1
feaB	1	1	1	1	1	1	1	1
pheA2	1	1	1	1	1	1	1	1
recD2	1	1	1	1	1	1	1	1
potE	1	1	1	1	1	1	1	1
lpxH	1	1	1	1	1	1	1	1
mutL	1	1	1	1	1	1	1	1
lplT	1	1	1	1	1	1	1	1
coq7	1	1	1	1	1	1	1	1
kdpE	1	1	1	1	1	1	1	1
folB	1	1	1	1	1	1	1	1
moeB	1	1	1	1	1	1	1	1
rpsR	1	1	1	1	1	1	1	1
glcC	1	1	1	1	1	1	1	1
eamB	1	1	1	1	1	1	1	1
COQ3	1	1	1	1	1	1	1	1
bepA	1	1	1	1	1	1	1	1
ghrB	1	1	1	1	1	1	1	1
rlhA	1	1	1	1	1	1	1	1
emrE	1	1	1	1	1	1	1	1
hisM	1	1	1	1	1	1	1	1
rpsB	1	1	1	1	1	1	1	1
ubiA	1	1	1	1	1	1	1	1
trpC	1	1	1	1	1	1	1	1
mutS2	1	1	1	1	1	1	1	1
entC	1	1	1	1	1	1	1	1
hdc	1	1	1	1	1	1	1	1
entB	1	1	1	1	1	1	1	1
fatC	1	1	1	1	1	1	1	1
fatD	1	1	1	1	1	1	1	1
thadh	1	1	1	1	1	1	1	1
yhhW	1	1	1	1	1	1	1	1
dacA	1	1	1	1	1	1	1	1
sohB	1	1	1	1	1	1	1	1
cysT	1	1	1	1	1	1	1	1
hipO	1	1	1	1	1	1	1	1
comEC	1	1	1	1	1	1	1	1
ybhR	1	1	1	1	1	1	1	1
etfA	1	1	1	1	1	1	1	1
dapF	1	1	1	1	1	1	1	1
pdxB	1	1	1	1	1	1	1	1
fdhD	1	1	1	1	1	1	1	1
drgA	1	1	1	1	1	1	1	1
mltF	1	1	1	1	1	1	1	1
folD	1	1	1	1	1	1	1	1
ftsH	1	1	1	1	1	1	1	1
greA	1	1	1	1	1	1	1	1
nhaP	1	1	1	1	1	1	1	1
trmL	1	1	1	1	1	1	1	1

sdsA	1	1	1	1	1	1	1	1
arnT	1	1	1	1	1	1	1	1
ispA	1	1	1	1	1	1	1	1
yhdE	1	1	1	1	1	1	1	1
cnbA	1	1	1	1	1	1	1	1
mdtC	1	1	1	1	1	1	1	1
rsmC	1	1	1	1	1	1	1	1
psiE	1	1	1	1	1	1	1	1
dgcN	1	1	1	1	1	1	1	1
fadH	1	1	1	1	1	1	1	1
kstR2	1	1	1	1	1	1	1	1
recG	1	1	1	1	1	1	1	1
trmD	1	1	1	1	1	1	1	1
ispH	1	1	1	1	1	1	1	1
mrcA	1	1	1	1	1	1	1	1
cat1	1	1	1	1	1	1	1	1
hisB	1	1	1	1	1	1	1	1
rnd	1	1	1	1	1	1	1	1
tenA	1	1	1	1	1	1	1	1
murG	1	1	1	1	1	1	1	1
pyrE	1	1	1	1	1	1	1	1
dsbB	1	1	1	1	1	1	1	1
mepA	1	1	1	1	1	1	1	1
phoR	1	1	1	1	1	1	1	1
psd	1	1	1	1	1	1	1	1
yciC	1	1	1	1	1	1	1	1
panS	1	1	1	1	1	1	1	1
pncA	1	1	1	1	1	1	1	1
yfdC	1	1	1	1	1	1	1	1
dkgB	1	1	1	1	1	1	1	1
mdtA	1	1	1	1	1	1	1	1
cysL	1	1	1	1	1	1	1	1
ilvD	1	1	1	1	1	1	1	1
rsmB	1	1	1	1	1	1	1	1
alx	1	1	1	1	1	1	1	1
rpmH	1	1	1	1	1	1	1	1
galU	1	1	1	1	1	1	1	1
ruvB	1	1	1	1	1	1	1	1
yjjV	1	1	1	1	1	1	1	1
yejF	1	1	1	1	1	1	1	1
czcD	1	1	1	1	1	1	1	1
trpF	1	1	1	1	1	1	1	1
ssuR	1	1	1	1	1	1	1	1
pgrR	1	1	1	1	1	1	1	1
fabG	1	1	1	1	1	1	1	1
yfcF	1	1	1	1	1	1	1	1
madC	1	1	1	1	1	1	1	1
arsC	1	1	1	1	1	1	1	1
hisI	1	1	1	1	1	1	1	1
puuA	1	1	1	1	1	1	1	1

kdpC	1	1	1	1	1	1	1	1
angR	1	1	1	1	1	1	1	1
fhuA	1	1	1	1	1	1	1	1
atoC	1	1	1	1	1	1	1	1
gdx	1	1	1	1	1	1	1	1
rsmD	1	1	1	1	1	1	1	1
sotB	1	1	1	1	1	1	1	1
azoR	1	1	1	1	1	1	1	1
citG	1	1	1	1	1	1	1	1
fabD	1	1	1	1	1	1	1	1
cysA	1	1	1	1	1	1	1	1
pliG	1	1	1	1	1	1	1	1
kdpD	1	1	1	1	1	1	1	1
pepD	1	1	1	1	1	1	1	1
yfhL	1	1	1	1	1	1	1	1
lgrE	1	1	1	1	1	1	1	1
ves	1	1	1	1	1	1	1	1
nudF	1	1	1	1	1	1	1	1
ephA	1	1	1	1	1	1	1	1
aac	1	1	1	1	1	1	1	1
add	1	1	1	1	1	1	1	1
otsB	1	1	1	1	1	1	1	1
ytcD	1	1	1	1	1	1	1	1
phnW	1	1	1	1	1	1	1	1
hyuE	1	1	1	1	1	1	1	1
cobS	1	1	1	1	1	1	1	1
pdhC	1	1	1	1	1	1	1	1
gatA	1	1	1	1	1	1	1	1
quiC	1	1	1	1	1	1	1	1
moeA	1	1	1	1	1	1	1	1
ampC	1	1	1	1	1	1	1	1
aroE	1	1	1	1	1	1	1	1
sttH	1	1	1	1	1	1	1	1
abaF	1	1	1	1	1	1	1	1
baiN	1	1	1	1	1	1	1	1
copA	1	1	1	1	1	1	1	1
cynR	1	1	1	1	1	1	1	1
cpnA	1	1	1	1	1	1	1	1
atsA	1	1	1	1	1	1	1	1
lvr	1	1	1	1	1	1	1	1
hcnC	1	1	1	1	1	1	1	1
madD	1	1	1	1	1	1	1	1
nnr	1	1	1	1	1	1	1	1
xcpX	1	1	1	1	1	1	1	1
nicP	1	1	1	1	1	1	1	1
yrfG	1	1	1	1	1	1	1	1
hcaT	1	1	1	1	1	1	1	1
eutC	1	1	1	1	1	1	1	1
kdpB	1	1	1	1	1	1	1	1
kdpA	1	1	1	1	1	1	1	1

eno	1	1	1	1	1	1	1	1
dapD	1	1	1	1	1	1	1	1
ispG	1	1	1	1	1	1	1	1
glyQ	1	1	1	1	1	1	1	1
syrM1	1	1	1	1	1	1	1	1
rpmB	1	1	1	1	1	1	1	1
secY	1	1	1	1	1	1	1	1
ilvI	1	1	1	1	1	1	1	1
glyA	1	1	1	1	1	1	1	1
yaeQ	1	1	1	1	1	1	1	1
panZ	1	1	1	1	1	1	1	1
ypjD	1	1	1	1	1	1	1	1
dgkA	1	1	1	1	1	1	1	1
grxD	1	1	1	1	1	1	1	1
rlmE	1	1	1	1	1	1	1	1
purE	1	1	1	1	1	1	1	1
ppsA	1	1	1	1	1	1	1	1
rpoD	1	1	1	1	1	1	1	1
hcp1	1	1	1	1	1	1	1	1
sucD	1	1	1	1	1	1	1	1
rpsK	1	1	1	1	1	1	1	1
iscU	1	1	1	1	1	1	1	1
cysD	1	1	1	1	1	1	1	1
fkpB	1	1	1	1	1	1	1	1
ilvC	1	1	1	1	1	1	1	1
yacG	1	1	1	1	1	1	1	1
rubA	1	1	1	1	1	1	1	1
corA	1	1	1	1	1	1	1	1
rpoZ	1	1	1	1	1	1	1	1
secB	1	1	1	1	1	1	1	1
pleD	1	1	1	1	1	1	1	1
mscL	1	1	1	1	1	1	1	1
rpsQ	1	1	1	1	1	1	1	1
bfd	1	1	1	1	1	1	1	1
rlmH	1	1	1	1	1	1	1	1
slyD	1	1	1	1	1	1	1	1
ybgC	1	1	1	1	1	1	1	1
yceD	1	1	1	1	1	1	1	1
lepA	1	1	1	1	1	1	1	1
glnL	1	1	1	1	1	1	1	1
trmO	1	1	1	1	1	1	1	1
rpsN	1	1	1	1	1	1	1	1
ispF	1	1	1	1	1	1	1	1
mreC	1	1	1	1	1	1	1	1
rplD	1	1	1	1	1	1	1	1
serA	1	1	1	1	1	1	1	1
rpmG	1	1	1	1	1	1	1	1
nuoE	1	1	1	1	1	1	1	1
cyoC	1	1	1	1	1	1	1	1
ydiK	1	1	1	1	1	1	1	1

atpG	1	1	1	1	1	1	1	1
gltA	1	1	1	1	1	1	1	1
pal	1	1	1	1	1	1	1	1
mrdB	1	1	1	1	1	1	1	1
ybaB	1	1	1	1	1	1	1	1
ribH	1	1	1	1	1	1	1	1
pnp	1	1	1	1	1	1	1	1
lptA	1	1	1	1	1	1	1	1
pyrH	1	1	1	1	1	1	1	1
dksA	1	1	1	1	1	1	1	1
rplR	1	1	1	1	1	1	1	1
orn	1	1	1	1	1	1	1	1
acpP	1	1	1	1	1	1	1	1
rplS	1	1	1	1	1	1	1	1
hom	1	1	1	1	1	1	1	1
serB2	1	1	1	1	1	1	1	1
rlmJ	1	1	1	1	1	1	1	1
rpsF	1	1	1	1	1	1	1	1
accD	1	1	1	1	1	1	1	1
yciB	1	1	1	1	1	1	1	1
argB	1	1	1	1	1	1	1	1
ilvH	1	1	1	1	1	1	1	1
upp	1	1	1	1	1	1	1	1
frr	1	1	1	1	1	1	1	1
ureG	1	1	1	1	1	1	1	1
tusA	1	1	1	1	1	1	1	1
gshB	1	1	1	1	1	1	1	1
sodB	1	1	1	1	1	1	1	1
panB	1	1	1	1	1	1	1	1
ompR	1	1	1	1	1	1	1	1
rpsH	1	1	1	1	1	1	1	1
slyX	1	1	1	1	1	1	1	1
atpE	1	1	1	1	1	1	1	1
dnaA	1	1	1	1	1	1	1	1
nuoK	1	1	1	1	1	1	1	1
yebC	1	1	1	1	1	1	1	1
hpt	1	1	1	1	1	1	1	1
rplO	1	1	1	1	1	1	1	1
ptsO	1	1	1	1	1	1	1	1
lemA	1	1	1	1	1	1	1	1
lptC	1	1	1	1	1	1	1	1
rnt	1	1	1	1	1	1	1	1
panC	1	1	1	1	1	1	1	1
prmB	1	1	1	1	1	1	1	1
greB	1	1	1	1	1	1	1	1
gloA	1	1	1	1	1	1	1	1
prfB	1	1	1	1	1	1	1	1
hpf	1	1	1	1	1	1	1	1
nfuA	1	1	1	1	1	1	1	1
der	1	1	1	1	1	1	1	1

adk	1	1	1	1	1	1	1	1
nuoI	1	1	1	1	1	1	1	1
gacA	1	1	1	1	1	1	1	1
IMPDH	1	1	1	1	1	1	1	1
doxA	1	1	1	1	1	1	1	1
acr1	1	1	1	1	1	1	1	1
yqeY	1	1	1	1	1	1	1	1
fabZ	1	1	1	1	1	1	1	1
ppnP	1	1	1	1	1	1	1	1
erpA	1	1	1	1	1	1	1	1
lptB	1	1	1	1	1	1	1	1
yibN	1	1	1	1	1	1	1	1
cspV	1	1	1	1	1	1	1	1
csrA	1	1	1	1	1	1	1	1
cyoD	1	1	1	1	1	1	1	1
guaB	1	1	1	1	1	1	1	1
dnaJ	1	1	1	1	1	1	1	1
alsT	1	1	1	1	1	1	1	1
psaB	1	1	1	1	1	1	1	1
yajC	1	1	1	1	1	1	1	1
rplQ	1	1	1	1	1	1	1	1
nuoB	1	1	1	1	1	1	1	1
pgsA	1	1	1	1	1	1	1	1
hupB	1	1	1	1	1	1	1	1
secE	1	1	1	1	1	1	1	1
ruvC	1	1	1	1	1	1	1	1
ilvA	1	1	1	1	1	1	1	1
rpmD	1	1	1	1	1	1	1	1
rplW	1	1	1	1	1	1	1	1
rplK	1	1	1	1	1	1	1	1
exoA	1	1	1	1	1	1	1	1
metXA	1	1	1	1	1	1	1	1
cspG	1	1	1	1	1	1	1	1
rpsD	1	1	1	1	1	1	1	1
iscS	1	1	1	1	1	1	1	1
hvrA	1	1	1	1	1	1	1	1
yiaD	1	1	1	1	1	1	1	1
iscX	1	1	1	1	1	1	1	1
rpsU	1	1	1	1	1	1	1	1
rplE	1	1	1	1	1	1	1	1
gmk	1	1	1	1	1	1	1	1
rpsT	1	1	1	1	1	1	1	1
gap2	1	1	1	1	1	1	1	1
ftsA	1	1	1	1	1	1	1	1
bigR	1	1	1	1	1	1	1	1
rnc	1	1	1	1	1	1	1	1
rcnR	1	1	1	1	1	1	1	1
rpsS	1	1	1	1	1	1	1	1
rpsM	1	1	1	1	1	1	1	1
rplC	1	1	1	1	1	1	1	1

fur	1	1	1	1	1	1	1	1
efp	1	1	1	1	1	1	1	1
infA	1	1	1	1	1	1	1	1
purC	1	1	1	1	1	1	1	1
tesB	1	1	1	1	1	1	1	1
nusG	1	1	1	1	1	1	1	1
rpsE	1	1	1	1	1	1	1	1
rplN	1	1	1	1	1	1	1	1
rplL	1	1	1	1	1	1	1	1
gpx1	1	1	1	1	1	1	1	1
ihfB	1	1	1	1	1	1	1	1
rppH	1	1	1	1	1	1	1	1
nadK	1	1	1	1	1	1	1	1
yjcH	1	1	1	1	1	1	1	1
msrB	1	1	1	1	1	1	1	1
ihfA	1	1	1	1	1	1	1	1
ettA	1	1	1	1	1	1	1	1
rpmJ	1	1	1	1	1	1	1	1
rplU	1	1	1	1	1	1	1	1
lspA	1	1	1	1	1	1	1	1
tsaC	1	1	1	1	1	1	1	1
ureA	1	1	1	1	1	1	1	1
rplA	1	1	1	1	1	1	1	1
paaB	1	1	1	1	1	1	1	1
grxC	1	1	1	1	1	1	1	1
nrdR	1	1	1	1	1	1	1	1
mraY	1	1	1	1	1	1	1	1
rstA	1	1	1	1	1	1	1	1
sdhD	1	1	1	1	1	1	1	1
minE	1	1	1	1	1	1	1	1
ftsL	1	1	1	1	1	1	1	1
ahpC	1	1	1	1	1	1	1	1
aroQ	1	1	1	1	1	1	1	1
rpmI	1	1	1	1	1	1	1	1
rpsI	1	1	1	1	1	1	1	1
mlaD	1	1	1	1	1	1	1	1
hisG	1	1	1	1	1	1	1	1
rplX	1	1	1	1	1	1	1	1
fis	1	1	1	1	1	1	1	1
phoP	1	1	1	1	1	1	1	1
rng	1	1	1	1	1	1	1	1
gloC	1	1	1	1	1	1	1	1
atoE	1	1	1	1	1	1	1	1
yveA	1	1	1	1	1	1	1	1
lolD	1	1	1	1	1	1	1	1
xanP	1	1	1	1	1	1	1	1
yceI	1	1	1	1	1	1	1	1
fadD	1	1	1	1	1	1	1	1
tpiA	1	1	1	1	1	1	1	1
clpX	1	1	1	1	1	1	1	1

astE	1	1	1	1	1	1	1	1
ung	1	1	1	1	1	1	1	1
yfcA	1	1	1	1	1	1	1	1
dapE	1	1	1	1	1	1	1	1
rlmN	1	1	1	1	1	1	1	1
ppx	1	1	1	1	1	1	1	1
catM	1	1	1	1	1	1	1	1
serS	1	1	1	1	1	1	1	1
btsR	1	1	1	1	1	1	1	1
sthA	1	1	1	1	1	1	1	1
rpoH	1	1	1	1	1	1	1	1
serC	1	1	1	1	1	1	1	1
dnaK	1	1	1	1	1	1	1	1
acnD	1	1	1	1	1	1	1	1
purL	1	1	1	1	1	1	1	1
pap	1	1	1	1	1	1	1	1
trxC	1	1	1	1	1	1	1	1
rpsA	1	1	1	1	1	1	1	1
ugpQ	1	1	1	1	1	1	1	1
miaA	1	1	1	1	1	1	1	1
zapE	1	1	1	1	1	1	1	1
ktrB	1	1	1	1	1	1	1	1
murJ	1	1	1	1	1	1	1	1
recA	1	1	1	1	1	1	1	1
ypeA	1	1	1	1	1	1	1	1
dmdC	1	1	1	1	1	1	1	1
uppP	1	1	1	1	1	1	1	1
purD	1	1	1	1	1	1	1	1
naiP	1	1	1	1	1	1	1	1
ilvE	1	1	1	1	1	1	1	1
aspS	1	1	1	1	1	1	1	1
ahpF	1	1	1	1	1	1	1	1
epsF	1	1	1	1	1	1	1	1
tsaD	1	1	1	1	1	1	1	1
nth	1	1	1	1	1	1	1	1
csaA	1	1	1	1	1	1	1	1
rplP	1	1	1	1	1	1	1	1
parE	1	1	1	1	1	1	1	1
scpA	1	1	1	1	1	1	1	1
kdgR	1	1	1	1	1	1	1	1
rutC	1	1	1	1	1	1	1	1
ompA	1	1	1	1	1	1	1	1
yegS	1	1	1	1	1	1	1	1
pgl	1	1	1	1	1	1	1	1
nuoF	1	1	1	1	1	1	1	1
rpsC	1	1	1	1	1	1	1	1
gbpR	1	1	1	1	1	1	1	1
aroP	1	1	1	1	1	1	1	1
dltA	1	1	1	1	1	1	1	1
cmk	1	1	1	1	1	1	1	1

gatB	1	1	1	1	1	1	1	1
mltG	1	1	1	1	1	1	1	1
dsbA	1	1	1	1	1	1	1	1
yafJ	1	1	1	1	1	1	1	1
yciV	1	1	1	1	1	1	1	1
cycA	1	1	1	1	1	1	1	1
yffB	1	1	1	1	1	1	1	1
rhlB	1	1	1	1	1	1	1	1
mnmC	1	1	1	1	1	1	1	1
accB	1	1	1	1	1	1	1	1
bpt	1	1	1	1	1	1	1	1
lldD	1	1	1	1	1	1	1	1
astC	1	1	1	1	1	1	1	1
clpP	1	1	1	1	1	1	1	1
yadG	1	1	1	1	1	1	1	1
parC	1	1	1	1	1	1	1	1
rseP	1	1	1	1	1	1	1	1
lptG	1	1	1	1	1	1	1	1
coaE	1	1	1	1	1	1	1	1
mfd	1	1	1	1	1	1	1	1
kdsD	1	1	1	1	1	1	1	1
lpdG	1	1	1	1	1	1	1	1
bamE	1	1	1	1	1	1	1	1
rarA	1	1	1	1	1	1	1	1
coaD	1	1	1	1	1	1	1	1
pdxA	1	1	1	1	1	1	1	1
nepI	1	1	1	1	1	1	1	1
iscR	1	1	1	1	1	1	1	1
aaeA	1	1	1	1	1	1	1	1
nadB	1	1	1	1	1	1	1	1
rtcB	1	1	1	1	1	1	1	1
hpd	1	1	1	1	1	1	1	1
pntB	1	1	1	1	1	1	1	1
purB	1	1	1	1	1	1	1	1
cydA	1	1	1	1	1	1	1	1
dnaN	1	1	1	1	1	1	1	1
fadB	1	1	1	1	1	1	1	1
bcp	1	1	1	1	1	1	1	1
gcd	1	1	1	1	1	1	1	1
dadA1	1	1	1	1	1	1	1	1
rsmH	1	1	1	1	1	1	1	1
leuB	1	1	1	1	1	1	1	1
argD	1	1	1	1	1	1	1	1
glmM	1	1	1	1	1	1	1	1
slt	1	1	1	1	1	1	1	1
yhgF	1	1	1	1	1	1	1	1
rhdA	1	1	1	1	1	1	1	1
oprM	1	1	1	1	1	1	1	1
mrpD	1	1	1	1	1	1	1	1
ygfZ	1	1	1	1	1	1	1	1

mexB	1	1	1	1	1	1	1	1
gpmI	1	1	1	1	1	1	1	1
rnpA	1	1	1	1	1	1	1	1
prc	1	1	1	1	1	1	1	1
sphX	1	1	1	1	1	1	1	1
astA	1	1	1	1	1	1	1	1
ppaX	1	1	1	1	1	1	1	1
ndhB	1	1	1	1	1	1	1	1
dacD	1	1	1	1	1	1	1	1
yabJ	1	1	1	1	1	1	1	1
argT	1	1	1	1	1	1	1	1
dat	1	1	1	1	1	1	1	1
maeB	1	1	1	1	1	1	1	1
glcB	1	1	1	1	1	1	1	1
rutG	1	1	1	1	1	1	1	1
echA8	1	1	1	1	1	1	1	1
rpoA	1	1	1	1	1	1	1	1
zur	1	1	1	1	1	1	1	1
dgt	1	1	1	1	1	1	1	1
hisH	1	1	1	1	1	1	1	1
oqxB9	1	1	1	1	1	1	1	1
ddc	1	1	1	1	1	1	1	1
ydhP	1	1	1	1	1	1	1	1
gatC	1	1	1	1	1	1	1	1
gutB	1	1	1	1	1	1	1	1
pdhR	1	1	1	1	1	1	1	1
brnQ	1	1	1	1	1	1	1	1
alkB	1	1	1	1	1	1	1	1
ribA	1	1	1	1	1	1	1	1
leuS	1	1	1	1	1	1	1	1
mrdA	1	1	1	1	1	1	1	1
ftsZ	1	1	1	1	1	1	1	1
scoB	1	1	1	1	1	1	1	1
nadC	1	1	1	1	1	1	1	1
degS	1	1	1	1	1	1	1	1
arnC	1	1	1	1	1	1	1	1
gntR	1	1	1	1	1	1	1	1
atpA	1	1	1	1	1	1	1	1
pcnB	1	1	1	1	1	1	1	1
rluB	1	1	1	1	1	1	1	1
ffh	1	1	1	1	1	1	1	1
metXS	1	1	1	1	1	1	1	1
ppc	1	1	1	1	1	1	1	1
argF	1	1	1	1	1	1	1	1
guaA	1	1	1	1	1	1	1	1
betT2	1	1	1	1	1	1	1	1
thrB	1	1	1	1	1	1	1	1
yadH	1	1	1	1	1	1	1	1
betB	1	1	1	1	1	1	1	1
glpG	1	1	1	1	1	1	1	1

mnmG	1	1	1	1	1	1	1	1
galE	1	1	1	1	1	1	1	1
recN	1	1	1	1	1	1	1	1
rplJ	1	1	1	1	1	1	1	1
sdhB	1	1	1	1	1	1	1	1
oxyR	1	1	1	1	1	1	1	1
yhjE	1	1	1	1	1	1	1	1
rsmA	1	1	1	1	1	1	1	1
cyoE	1	1	1	1	1	1	1	1
ktrA	1	1	1	1	1	1	1	1
ipdC	1	1	1	1	1	1	1	1
cyoB	1	1	1	1	1	1	1	1
nuoJ	1	1	1	1	1	1	1	1
ydjA	1	1	1	1	1	1	1	1
parA	1	1	1	1	1	1	1	1
dmlA	1	1	1	1	1	1	1	1
argG	1	1	1	1	1	1	1	1
lysS	1	1	1	1	1	1	1	1
rimM	1	1	1	1	1	1	1	1
ttcA	1	1	1	1	1	1	1	1
norM	1	1	1	1	1	1	1	1
envZ	1	1	1	1	1	1	1	1
epsE	1	1	1	1	1	1	1	1
yeaX	1	1	1	1	1	1	1	1
icd	1	1	1	1	1	1	1	1
antA	1	1	1	1	1	1	1	1
RBKS	1	1	1	1	1	1	1	1
pyrC	1	1	1	1	1	1	1	1
yjiE	1	1	1	1	1	1	1	1
nuoC	1	1	1	1	1	1	1	1
otsA	1	1	1	1	1	1	1	1
xerD	1	1	1	1	1	1	1	1
pilT	1	1	1	1	1	1	1	1
blc	1	1	1	1	1	1	1	1
cca	1	1	1	1	1	1	1	1
trpB	1	1	1	1	1	1	1	1
bioB	1	1	1	1	1	1	1	1
amtB	1	1	1	1	1	1	1	1
prsE	1	1	1	1	1	1	1	1
rne	1	1	1	1	1	1	1	1
aqpZ2	1	1	1	1	1	1	1	1
ald1	1	1	1	1	1	1	1	1
map	1	1	1	1	1	1	1	1
tusD	1	1	1	1	1	1	1	1
ligA	1	1	1	1	1	1	1	1
hutU	1	1	1	1	1	1	1	1
rapA	1	1	1	1	1	1	1	1
ychF	1	1	1	1	1	1	1	1
ohrB	1	1	1	1	1	1	1	1
exaE	1	1	1	1	1	1	1	1

ppsR	1	1	1	1	1	1	1	1
pyrB	1	1	1	1	1	1	1	1
ppk	1	1	1	1	1	1	1	1
polA	1	1	1	1	1	1	1	1
ribBA	1	1	1	1	1	1	1	1
dcd	1	1	1	1	1	1	1	1
kup	1	1	1	1	1	1	1	1
ydaD	1	1	1	1	1	1	1	1
caiA	1	1	1	1	1	1	1	1
pstB	1	1	1	1	1	1	1	1
oprB	1	1	1	1	1	1	1	1
queG	1	1	1	1	1	1	1	1
hyi	1	1	1	1	1	1	1	1
atpB	1	1	1	1	1	1	1	1
aceE	1	1	1	1	1	1	1	1
mnhC1	1	1	1	1	1	1	1	1
proB	1	1	1	1	1	1	1	1
gntK	1	1	1	1	1	1	1	1
ftsY	1	1	1	1	1	1	1	1
dnaE	1	1	1	1	1	1	1	1
iucA	1	1	1	1	1	1	1	1
yhjQ	1	1	1	1	1	1	1	1
nasD	1	1	1	1	1	1	1	1
rbfA	1	1	1	1	1	1	1	1
metR	1	1	1	1	1	1	1	1
thyA	1	1	1	1	1	1	1	1
fosB	1	1	1	1	1	1	1	1
nusB	1	1	1	1	1	1	1	1
sucC	1	1	1	1	1	1	1	1
lptF	1	1	1	1	1	1	1	1
queC	1	1	1	1	1	1	1	1
sucA	1	1	1	1	1	1	1	1
dinB	1	1	1	1	1	1	1	1
argS	1	1	1	1	1	1	1	1
rlpA	1	1	1	1	1	1	1	1
nemR	1	1	1	1	1	1	1	1
queE	1	1	1	1	1	1	1	1
pdxH	1	1	1	1	1	1	1	1
era	1	1	1	1	1	1	1	1
trpA	1	1	1	1	1	1	1	1
paaK	1	1	1	1	1	1	1	1
kbp	1	1	1	1	1	1	1	1
iscA	1	1	1	1	1	1	1	1
lldR	1	1	1	1	1	1	1	1
pepA	1	1	1	1	1	1	1	1
proA	1	1	1	1	1	1	1	1
putA	1	1	1	1	1	1	1	1
gshA	1	1	1	1	1	1	1	1
paaX	1	1	1	1	1	1	1	1
nuoN	1	1	1	1	1	1	1	1

carB	1	1	1	1	1	1	1	1
aroC	1	1	1	1	1	1	1	1
fbp	1	1	1	1	1	1	1	1
metI	1	1	1	1	1	1	1	1
loiP	1	1	1	1	1	1	1	1
glpE	1	1	1	1	1	1	1	1
purA	1	1	1	1	1	1	1	1
thrC	1	1	1	1	1	1	1	1
recF	1	1	1	1	1	1	1	1
tgt	1	1	1	1	1	1	1	1
ltxB	1	1	1	1	1	1	1	1
hutH	1	1	1	1	1	1	1	1
anmK	1	1	1	1	1	1	1	1
rpiA	1	1	1	1	1	1	1	1
scoA	1	1	1	1	1	1	1	1
argH	1	1	1	1	1	1	1	1
epmB	1	1	1	1	1	1	1	1
fabI	1	1	1	1	1	1	1	1
rluC	1	1	1	1	1	1	1	1
entS	1	1	1	1	1	1	1	1
eriC	1	1	1	1	1	1	1	1
tyrS	1	1	1	1	1	1	1	1
hslO	1	1	1	1	1	1	1	1
tdcB	1	1	1	1	1	1	1	1
dusC	1	1	1	1	1	1	1	1
nuoH	1	1	1	1	1	1	1	1
ndk	1	1	1	1	1	1	1	1
hflX	1	1	1	1	1	1	1	1
nadE	1	1	1	1	1	1	1	1
gsiA	1	1	1	1	1	1	1	1
qseB	1	1	1	1	1	1	1	1
fadE	1	1	1	1	1	1	1	1
yidC	1	1	1	1	1	1	1	1
ppa	1	1	1	1	1	1	1	1
purF	1	1	1	1	1	1	1	1
nrdB	1	1	1	1	1	1	1	1
nemA	1	1	1	1	1	1	1	1
dnaB	1	1	1	1	1	1	1	1
rplV	1	1	1	1	1	1	1	1
lptE	1	1	1	1	1	1	1	1
hisF	1	1	1	1	1	1	1	1
lysP	1	1	1	1	1	1	1	1
rpoB	1	1	1	1	1	1	1	1
infB	1	1	1	1	1	1	1	1
mrcB	1	1	1	1	1	1	1	1
rimP	1	1	1	1	1	1	1	1
trmJ	1	1	1	1	1	1	1	1
thrS	1	1	1	1	1	1	1	1
rplM	1	1	1	1	1	1	1	1
hscA	1	1	1	1	1	1	1	1

ybeY	1	1	1	1	1	1	1	1
ftsQ	1	1	1	1	1	1	1	1
rluD	1	1	1	1	1	1	1	1
wax-dgaT	1	1	1	1	1	1	1	1
fumB	1	1	1	1	1	1	1	1
cbbA	1	1	1	1	1	1	1	1
cysG	1	1	1	1	1	1	1	1
ycgM	1	1	1	1	1	1	1	1
mdh	1	1	1	1	1	1	1	1
cysS	1	1	1	1	1	1	1	1
murA	1	1	1	1	1	1	1	1
mqo	1	1	1	1	1	1	1	1
ybeZ	1	1	1	1	1	1	1	1
clpS	1	1	1	1	1	1	1	1
glmU	1	1	1	1	1	1	1	1
rsfS	1	1	1	1	1	1	1	1
holA	1	1	1	1	1	1	1	1
asd	1	1	1	1	1	1	1	1
mtcA2	1	1	1	1	1	1	1	1
bamB	1	1	1	1	1	1	1	1
rpoC	1	1	1	1	1	1	1	1
dxs	1	1	1	1	1	1	1	1
gltT	1	1	1	1	1	1	1	1
ydfG	1	1	1	1	1	1	1	1
mtgA	1	1	1	1	1	1	1	1
tktA	1	1	1	1	1	1	1	1
yciO	1	1	1	1	1	1	1	1
trmA	1	1	1	1	1	1	1	1
ftsN	1	1	1	1	1	1	1	1
rnhA	1	1	1	1	1	1	1	1
abaQ	1	1	1	1	1	1	1	1
priA	1	1	1	1	1	1	1	1
pcaG	1	1	1	1	1	1	1	1
obg	1	1	1	1	1	1	1	1
coaBC	1	1	1	1	1	1	1	1
sir	1	1	1	1	1	1	1	1
dusB	1	1	1	1	1	1	1	1
nusA	1	1	1	1	1	1	1	1
mlaC	1	1	1	1	1	1	1	1
ileS	1	1	1	1	1	1	1	1
prmA	1	1	1	1	1	1	1	1
alaS	1	1	1	1	1	1	1	1
sdhA	1	1	1	1	1	1	1	1
tusE	1	1	1	1	1	1	1	1
qmcA	1	1	1	1	1	1	1	1
putP	1	1	1	1	1	1	1	1
emrK	1	1	1	1	1	1	1	1
prfA	1	1	1	1	1	1	1	1
rnr	1	1	1	1	1	1	1	1
lpxC	1	1	1	1	1	1	1	1

hflD	1	1	1	1	1	1	1	1
cbpM	1	1	1	1	1	1	1	1
mltB	1	1	1	1	1	1	1	1
fadI	1	1	1	1	1	1	1	1
gltX	1	1	1	1	1	1	1	1
mrpG	1	1	1	1	1	1	1	1
gyrB	1	1	1	1	1	1	1	1
yfdE	1	1	1	1	1	1	1	1
aat	1	1	1	1	1	1	1	1
maeA	1	1	1	1	1	1	1	1
minD	1	1	1	1	1	1	1	1
gltS	1	1	1	1	1	1	1	1
ackA	1	1	1	1	1	1	1	1
dut	1	1	1	1	1	1	1	1
glnG	1	1	1	1	1	1	1	1
suhB	1	1	1	1	1	1	1	1
pgk	1	1	1	1	1	1	1	1
hemL	1	1	1	1	1	1	1	1
queF	1	1	1	1	1	1	1	1
trpS2	1	1	1	1	1	1	1	1
gyrA	1	1	1	1	1	1	1	1
ycaC	1	1	1	1	1	1	1	1
uvrB	1	1	1	1	1	1	1	1
secA	1	1	1	1	1	1	1	1
gltI	1	1	1	1	1	1	1	1
hscB	1	1	1	1	1	1	1	1
pyrG	1	1	1	1	1	1	1	1
miaB	1	1	1	1	1	1	1	1
carA	1	1	1	1	1	1	1	1
cysE	1	1	1	1	1	1	1	1
yciI	1	1	1	1	1	1	1	1
prpB	1	1	1	1	1	1	1	1
rsgA	1	1	1	1	1	1	1	1
metF	1	1	1	1	1	1	1	1
ahcY	1	1	1	1	1	1	1	1
baeR	1	1	1	1	1	1	1	1
nuoM	1	1	1	1	1	1	1	1
pckG	1	1	1	1	1	1	1	1
atpC	1	1	1	1	1	1	1	1
tatC	1	1	1	1	1	1	1	1
tufA	1	1	1	1	1	1	1	1
tolQ	1	1	1	1	1	0	1	1
cntB	1	1	1	0	1	1	1	1
pdtaR	1	1	1	1	1	1	1	1
chbG	1	1	0	1	1	1	1	1
znuA	0	1	1	1	1	1	1	1
epsF	1	1	1	0	1	1	1	1
gltP	0	1	1	1	1	1	1	1
lgt	1	1	1	1	1	1	1	1
thcD	1	1	1	0	1	1	1	1

ureC	1	1	0	1	1	1	1	1
clpB	1	1	1	0	1	1	1	1
rlmL	1	1	0	1	1	1	1	1
dmoA	1	1	1	1	1	1	1	1
hdfR	1	1	1	1	1	1	1	1
amgK	1	1	1	1	1	1	1	1
tolQ	1	1	1	0	1	1	1	1
gdhB	1	1	1	1	1	1	1	1
recX	0	1	1	1	1	1	1	1
fhuE	1	1	1	1	1	1	1	1
tsf	1	1	1	1	1	1	1	1
tcyP	1	1	1	1	0	1	1	1
typA	0	1	1	1	1	1	1	1
plsB	1	1	1	1	1	1	1	1
gltD	1	1	1	1	1	1	1	1
murE	1	1	1	1	1	1	1	1
gabP	1	1	1	1	1	1	1	1
waaA	1	1	1	1	1	1	1	1
hiuH	1	1	0	1	1	1	1	1
dtd	1	1	1	1	1	1	1	1
mnmE	1	1	1	1	1	1	1	1
trpB	1	1	1	1	1	1	1	1
pheP	1	1	1	1	1	1	1	1
iutA	1	1	1	1	1	1	1	1
mdaB	1	1	1	1	1	1	1	1
metQ	1	1	1	1	0	1	1	1
rph	1	1	1	0	1	1	1	1
epsE	1	1	1	1	1	1	1	1
eptA	1	1	1	1	1	1	1	1
naiP	1	1	1	1	1	1	1	1
yhhW	1	0	1	1	1	1	1	1
adeP	1	0	1	1	1	1	1	1
ycgL	1	1	1	1	1	1	1	1
fruA	1	1	1	1	1	1	1	1
bicA	1	1	1	1	1	1	1	1
ubiC	1	1	1	1	1	1	1	1
dtpT	1	1	1	1	1	1	1	1
yidD	1	1	1	1	1	1	1	1
cmoA	1	1	1	0	1	1	1	1
dhfrIII	1	1	1	1	1	1	1	1
pknD	1	1	1	1	1	1	1	1
eamA	1	1	1	1	1	1	1	1
czcA	1	1	0	1	1	1	1	1
dnaG	1	1	0	1	1	1	1	1
betT1	1	1	1	1	1	1	1	1
lip1	1	1	1	1	1	1	1	1
hcnB	1	1	1	1	1	1	1	1
oppA	1	1	1	1	1	1	1	1
ppiD	1	1	1	1	1	1	1	1
cntA	1	1	1	1	1	1	1	1

folP	1	1	1	0	1	1	1	1
fdx	1	1	1	0	1	1	1	1
secF	1	1	1	1	1	1	1	1
tufA	1	1	1	1	1	0	1	1
menH	1	1	1	1	1	1	1	1
cpdA	1	1	1	0	1	1	1	1
fabG	1	1	1	1	1	1	1	1
ygeA	1	1	1	1	0	1	1	1
cstA	1	1	1	1	1	1	1	1
prs	0	1	1	1	1	1	1	1
perR	1	1	1	1	1	1	1	1
fadH	1	1	1	1	1	1	1	1
tynA	1	1	1	1	1	1	1	1
hsaA	1	1	1	1	1	1	1	0
cpoB	0	1	1	1	1	1	1	1
nasA	1	1	1	0	1	1	1	1
nudJ	1	1	1	0	0	1	1	1
btuD	1	0	1	1	1	1	1	1
anoI	1	1	1	1	1	1	1	1
anoR	1	1	1	1	1	1	1	1
fucA	1	1	1	1	1	1	1	0
tgnC	1	1	1	1	1	1	1	1
tgnE	1	1	1	1	1	1	1	1
moaA	1	1	1	1	1	1	1	0
cat	1	1	1	0	1	0	1	1
pspB	1	1	1	0	1	1	1	1
aptB	1	1	1	1	1	1	1	1
pksN	1	1	1	1	1	1	1	1
echA8	1	1	1	0	1	1	1	1
nicT	1	1	1	1	1	1	1	1
aptA	1	1	1	1	1	1	1	1
tgnD	1	1	1	1	1	1	1	1
tgnA	1	1	1	1	1	1	1	1
tgnB	1	1	1	1	1	1	1	1
fecE	1	1	1	1	1	1	1	0
adhA	1	1	1	1	1	1	1	0
yddG	1	1	1	1	0	1	1	1
pqiB	1	1	1	1	1	1	0	1
hfq	1	1	1	1	1	1	1	1
ygiN	1	1	1	1	1	1	1	1
rsmE	1	1	1	0	1	0	1	1
htrE	1	1	1	1	1	1	1	1
pcaF	1	1	1	1	1	1	1	1
oprD	1	1	1	1	1	1	1	1
budC	1	0	1	1	0	1	1	1
styA	1	0	1	1	1	1	1	1
yclQ	1	1	1	1	1	1	1	0
napA	1	1	1	1	1	1	1	0
galE	1	1	1	1	1	1	1	0
choD	1	0	1	1	0	1	1	1

fecI	1	1	1	1	1	1	1	1
rfnT	1	1	1	0	1	1	1	1
ybhS	1	1	1	1	1	1	1	0
metH	1	1	1	1	1	1	1	0
aarA	1	1	1	1	1	1	0	1
yigZ	1	1	1	1	1	1	1	1
soxR	1	0	1	1	0	1	1	1
quiA	1	1	1	0	1	1	1	1
rplI	1	1	1	1	1	1	1	0
aprE	1	1	1	1	1	1	1	1
ybhR	1	1	1	1	1	1	1	0
acrB	1	1	1	1	1	1	1	1
pigA	1	1	1	1	1	1	1	1
dhbF	1	1	1	1	1	1	1	1
yybR	1	1	1	1	1	1	1	1
mexA	1	1	1	1	1	1	1	1
srrA	1	1	1	1	1	1	1	1
sadH	1	0	1	1	0	1	1	1
namA	1	1	1	0	1	1	1	1
mlaE	1	1	1	1	1	1	1	0
pgpA	1	1	1	0	1	1	1	1
ybhF	1	1	1	1	1	1	1	0
comM	1	1	1	1	1	1	1	1
ada	1	1	1	1	1	1	1	1
yddE	1	0	1	1	0	1	1	1
hdfR	1	1	1	1	1	1	0	1
ribF	1	0	1	1	0	1	1	1
caf1A	1	1	1	1	1	1	1	0
nicP	1	0	1	1	0	1	1	1
dapB	1	1	1	1	1	1	1	0
yadV	1	1	1	1	1	1	1	1
bshA	1	1	1	0	1	1	1	1
virS	1	1	1	1	1	1	1	1
yejE	1	1	1	1	1	1	1	0
lipR	1	0	1	0	0	1	1	1
hcaB	1	1	1	1	1	1	1	1
yddE	1	1	1	1	1	1	0	1
group	1	1	1	0	1	1	1	0
namA	0	1	0	1	1	0	1	1
mdtE	1	0	1	1	0	1	1	0
topA	1	1	1	1	1	1	1	1
puuB	1	0	1	1	0	1	1	1
puuP	1	0	1	1	0	1	1	1
gpsA	0	0	1	1	0	0	1	1
rbn	1	1	1	0	1	1	1	1
purK	1	1	1	1	1	1	1	0
aroA	1	1	1	0	1	1	1	1
modA	1	0	1	1	0	1	1	0
pcaK	1	0	1	0	0	1	1	1
dmlR	0	1	0	1	1	0	1	1

nagR	0	1	0	1	1	0	1	1
ttuB	0	1	0	1	1	0	1	1
iscR	0	1	0	1	1	0	1	1
prr	1	0	1	1	0	1	1	1
vdh	1	0	1	1	0	1	1	1
tsaM1	1	0	1	1	0	1	1	1
slmA	0	1	0	1	1	0	1	1
emrY	0	1	0	1	1	0	1	1
relE	1	1	1	1	1	1	1	1
pcpD	1	0	1	1	0	1	1	1
sauU	0	1	0	1	1	0	1	1
secD	1	1	1	1	1	1	1	1
astC	1	0	0	1	0	1	1	1
yfcG	0	1	1	1	1	1	1	0
menE	1	0	1	0	0	1	1	1
cycA	1	0	0	1	0	1	1	1
aroP	1	0	1	1	0	1	1	1
esiB	1	1	1	0	1	1	0	1
bioH	1	0	1	1	0	1	0	1
astA	1	0	0	1	0	1	1	1
fatA	1	1	1	0	1	1	0	1
rcsC	1	0	1	1	1	1	0	0
rplT	0	1	0	1	1	1	1	0
atsA	1	0	1	1	0	1	1	0
gpmA	1	1	1	1	1	1	0	0
ydhP	1	1	1	1	1	1	1	0
fimW	1	1	1	1	0	1	0	0
cntI	1	0	0	1	0	1	1	1
pepO	1	0	1	1	0	1	1	0
garD	1	0	0	1	0	1	1	1
yjcH	1	1	1	1	1	1	0	0
norR	1	1	1	1	1	1	0	0
gno	1	1	1	1	1	1	0	0
gudP	1	0	0	1	0	1	1	1
lutR	1	0	0	1	0	1	1	1
cpo	1	1	1	1	1	1	0	0
fhuE	1	0	1	1	0	1	1	0
smf-1	0	0	0	1	0	1	1	1
asnC	1	0	0	1	0	1	1	1
sbmA	1	0	1	1	0	1	1	0
smvA	1	1	1	0	1	1	0	1
calB	1	1	1	1	1	1	0	0
actP	1	1	1	1	1	1	0	0
dmdB	1	1	1	1	1	1	0	0
shlB	1	0	1	1	0	1	1	0
mlaF	1	0	0	1	0	1	0	1
betB	1	0	1	1	1	1	0	0
mhpT	1	0	1	1	0	1	1	0
gloB	0	1	0	1	1	0	1	1
ppiA	0	1	1	1	1	0	0	1

fatA	1	1	1	0	1	1	1	0
dcyD	0	1	0	1	1	0	1	1
pcaJ	1	1	1	0	1	1	0	1
pcaJ	0	1	0	0	1	0	1	1
yjdF	0	1	0	1	0	0	1	1
wbpA	1	1	0	1	0	1	1	0
group	1	1	1	1	1	1	0	0
mphL	1	0	1	1	0	1	1	0
bfrD	1	0	1	1	0	1	0	0
tauB	1	0	1	0	0	1	1	0
czcB	1	1	1	0	1	1	1	0
czcA	1	1	1	0	1	1	1	0
xamoA	1	0	1	1	0	1	1	0
aspT	1	1	1	0	1	1	1	0
asD	1	1	1	0	1	1	1	0
esiB	1	1	1	0	1	1	1	0
glpD	0	1	0	1	1	0	1	1
cntO	0	1	1	0	1	0	0	1
mphP	1	0	1	1	0	1	1	0
dmpM	1	0	1	1	0	1	1	0
ptk	1	1	1	1	1	1	0	0
mopR	1	0	0	1	0	1	1	0
pgi	0	1	0	1	1	0	1	0
ompW	0	0	1	0	0	1	1	0
yyaP	0	1	0	1	1	0	1	0
lexA	1	1	1	0	1	1	0	0
glnE	1	0	1	1	1	1	0	0
neo	1	0	1	0	1	1	0	0
ycjY	1	0	1	0	0	1	0	1
intS	0	1	0	1	1	0	1	1
eptA	1	1	0	0	1	1	1	0
mdcG	1	1	1	0	1	1	0	0
comM	0	1	0	0	1	0	1	0
gudD	0	0	0	1	0	0	1	1
oprM	1	1	1	0	1	1	0	0
ptp	1	1	1	1	1	1	0	0
pgrR	1	0	1	0	0	1	0	1
bauC	1	1	1	0	1	1	0	0
gcvA	1	0	1	0	0	1	1	0
ethR	1	1	1	0	1	1	0	0
pao	1	1	1	0	1	1	0	0
aidA	1	1	1	0	1	1	0	0
xcpQ	1	0	1	0	0	1	1	0
rhtC	0	1	0	1	1	0	0	1
amtB	1	0	1	1	0	1	0	0
czcR	0	0	0	1	0	0	1	1
hiuH	0	0	0	1	0	0	1	1
COQ3	0	0	0	1	0	0	1	1
blc	0	0	0	1	0	0	1	1
pmfR	1	1	1	0	1	1	0	0

glmM	1	0	1	0	1	1	0	0
dnaB	1	0	1	0	1	1	1	0
hmrR	0	1	0	1	1	0	0	0
ohrR	0	1	0	1	1	0	0	0
aes	0	1	0	1	1	0	0	0
cusR	0	0	0	1	0	0	1	1
ufaA1	0	0	0	1	0	0	1	1
btuB	0	1	0	0	1	0	1	0
exoX	0	0	0	1	1	0	1	1
uctC	1	0	1	1	0	1	0	0
katA	0	1	0	1	1	0	0	0
intS	0	1	0	1	1	0	0	0
folP	1	0	1	0	0	1	0	0
epsL	0	1	0	1	1	0	0	0
pac	0	1	0	0	1	0	1	1
ata	1	0	1	0	0	1	0	0
czcC	0	0	0	1	0	0	0	1
rppH	1	0	1	0	0	1	0	1
gdhB	0	1	0	0	1	0	0	1
scmP	0	1	0	0	1	0	0	1
pglF	0	1	0	1	0	0	1	0
recD2	1	0	1	0	0	1	0	0
ycaC	0	1	0	0	1	0	0	1
dmlR	0	1	0	0	1	0	0	1
kefC	0	1	0	0	1	0	0	1
czcS	0	0	0	1	0	0	1	1
yngG	0	0	0	1	0	0	1	1
smtB	0	0	0	1	0	0	1	1
argP	0	0	0	1	0	0	1	1
vgrG1	0	1	0	0	0	0	1	1
xerC	0	0	0	0	0	0	1	1
cdhR	1	0	1	0	0	1	0	0
wbpI	0	0	1	0	0	0	1	0
wbjC	0	0	1	0	0	0	1	0
novN	1	0	1	0	0	1	0	0
ipsA	1	0	1	0	0	1	0	0
betA	1	0	1	0	0	1	0	0
recF	1	0	1	0	0	1	0	0
xerC	0	0	0	0	0	0	1	0
ssuA	0	1	0	0	1	0	1	1
dkgA	1	0	1	0	0	1	0	0
nudL	1	0	1	0	0	1	0	0
nagZ	1	0	1	0	0	1	0	0
alkJ	1	0	1	0	0	1	0	0
ilvB	1	0	1	0	0	1	0	0
lamB	1	0	1	0	0	1	0	0
thiG	1	0	1	0	0	1	0	0
hisN	1	0	1	0	0	1	0	0
livQ	1	0	1	0	0	1	0	0
hpsN	1	0	1	0	0	1	0	0

yohK	1	0	1	0	0	1	0	0
cytR	1	0	1	0	0	1	0	0
clcD	1	0	1	0	0	1	0	0
ybjJ	1	0	1	0	0	1	0	0
bacC	1	0	1	0	0	1	0	0
nadX	1	0	1	0	0	1	0	0
rutD	1	0	1	0	0	1	0	0
puuC	1	0	1	0	0	1	0	0
farR	1	0	1	0	0	1	0	0
C1-hpah	1	0	1	0	0	1	0	0
C2-hpah	1	0	1	0	0	1	0	0
hpcH	1	0	1	0	0	1	0	0
hpcG	1	0	1	0	0	1	0	0
hpcD	1	0	1	0	0	1	0	0
hpcB	1	0	1	0	0	1	0	0
hpcE	1	0	1	0	0	1	0	0
btr	1	0	1	0	0	1	0	0
bcr	1	0	1	0	0	1	0	0
tyrS	1	0	1	0	0	1	0	0
betI	1	0	1	0	0	1	0	0
yejK	1	0	1	0	0	1	0	0
cirA	1	0	1	0	0	1	0	0
azoR1	1	0	1	0	0	1	0	0
dpnM	0	1	0	0	0	0	0	0
pglE	0	1	0	1	0	0	1	0
tnsB	0	1	0	0	1	0	0	1
cusR	0	0	0	0	0	0	1	1
mrkD	1	0	1	0	0	1	0	0
davD	1	0	1	0	0	1	0	0
yehB	0	0	0	0	0	0	1	1
rfbD	1	0	1	0	0	1	0	0
arpC	1	0	1	0	0	1	0	0
cugP	0	1	0	1	0	0	0	0
prlC	0	0	1	0	0	0	0	0
umuC	0	1	0	0	1	0	1	1
fic	0	0	0	0	0	0	0	1
mupP	0	1	0	0	1	0	1	0
silP	0	0	0	0	0	0	1	1
pilA	1	0	1	0	0	1	1	0
legG	0	1	0	1	0	0	1	0
yobA	0	0	0	0	0	0	1	1
cusS	0	0	0	0	0	0	1	1
nimR	0	0	0	1	0	0	1	0
fadD	0	0	0	0	0	0	1	0
legI	0	1	0	1	0	0	1	0
cmdD	0	1	0	0	1	0	0	0
ligK	0	1	0	0	1	0	1	0
lexA	0	1	0	1	0	0	0	0
tagO	0	0	1	0	0	0	0	0
gnu	0	0	1	0	0	0	0	0

ssuC	0	0	0	1	0	0	1	0
kipR	0	1	0	0	1	0	1	0
panS	0	0	0	0	0	0	1	0
golD	0	0	0	0	0	0	1	0
pcpR	0	0	0	0	0	0	1	0
prtR	1	0	0	0	0	1	0	0
yejK	0	1	0	0	1	0	0	0
yddG	0	1	0	0	1	0	0	1
eutQ	0	1	0	0	1	0	0	1
rubB	0	1	0	0	1	0	0	1
gbsA	0	1	0	0	1	0	0	1
yeaW	0	1	0	0	1	0	0	1
uvrA	0	0	0	1	0	0	1	0
recE	1	0	1	0	0	1	1	0
cas3	0	0	0	1	0	0	0	1
msbA	0	0	0	1	0	0	0	0
yejK	1	0	1	0	0	1	0	0
cas1	0	0	0	1	0	0	0	1
csy2	0	0	0	1	0	0	0	1
btuB	0	0	0	1	0	0	0	0
lspA	0	0	0	0	0	0	0	1
feoB	0	1	0	0	0	0	0	0
merR1	0	0	0	0	0	0	0	1
dnaA	1	0	0	0	1	1	0	0
prtR	1	0	1	0	0	1	0	0
intA	1	0	1	0	0	1	0	0
queC	0	1	0	0	1	0	0	1
ycfH	0	1	0	0	1	0	0	1
rmtB	0	1	0	0	1	0	0	1
hupB	0	0	1	0	0	1	0	0
capD	0	0	1	0	0	0	0	0
csy1	0	0	0	1	0	0	0	1
csy3	0	0	0	1	0	0	0	1
cas6f	0	0	0	1	0	0	0	1
rtcB	0	0	0	1	0	0	0	0
rbn	0	0	0	1	0	0	0	0
rhaR	0	0	0	1	0	0	1	0
tnsB	0	1	0	0	1	0	0	1
tetR	0	0	0	0	1	0	0	1
tetA	0	0	0	0	1	0	0	1
hscC	0	0	0	0	0	0	1	0
bicA	0	0	0	0	1	0	1	1
shlB	0	0	0	1	0	0	1	0
ttuB	0	0	0	1	0	0	0	0
aphA	0	0	0	1	0	0	0	1
tatB	0	0	0	1	0	0	0	0
tatA	0	0	0	1	0	0	0	0
plcN	0	1	0	0	0	0	0	0
folP	0	1	0	0	0	0	0	1
ftsP	0	0	0	0	0	0	0	1

arsH	0	0	0	0	0	0	0	0
acr3	0	0	0	0	0	0	0	0
arsC	0	0	0	0	0	0	0	0
ttdB	0	0	0	1	0	0	0	0
ttdA	0	0	0	1	0	0	0	0
dmlR	0	0	0	1	0	0	0	0
ssuD	0	0	0	1	0	0	0	0
gsiB	0	0	0	1	0	0	0	0
gsiC	0	0	0	1	0	0	0	0
soxB	0	0	0	1	0	0	0	0
sfnR	0	0	0	1	0	0	0	0
tatC	0	0	0	1	0	0	0	0
ssuC	0	0	0	1	0	0	0	0
oppA	0	0	0	1	0	0	0	0
cntO	0	0	0	1	0	0	0	0
btuB	0	1	0	0	1	0	0	0
ccrA2	0	1	0	0	1	0	0	0
gst	0	1	0	0	1	0	0	0
yybR	0	0	0	1	0	0	0	1
msr(E)	0	0	0	0	1	0	0	1
djlA	0	0	0	0	0	0	1	1
blaNDM-1	0	0	0	0	0	0	1	0
yjgH	0	0	0	0	0	0	1	0
ant1	0	1	0	0	0	0	0	1
tnsE	0	0	0	0	0	0	0	1
tnsC	0	0	0	0	0	0	0	1
tnsB	0	0	0	0	0	0	0	1
tnsA	0	0	0	0	0	0	0	1
mhpC	0	0	0	0	0	0	0	1
cspG	0	0	0	0	0	0	0	1
cntB	0	0	0	1	0	0	0	0
pcaJ	0	0	0	1	0	0	0	0
gltP	1	0	0	0	0	0	0	0
btuD	0	1	0	1	0	0	0	0
gltD	0	0	1	0	0	0	0	0
yhjE	1	0	1	0	0	1	0	0
ugd	1	0	1	0	0	1	0	0
groL	0	0	0	0	0	0	0	1
xerC	0	1	0	0	0	0	0	1
umuC	0	1	0	0	0	0	0	0
sauU	0	0	0	1	0	0	0	0
dgcM	0	0	0	0	0	0	1	0
nicC	0	0	0	1	0	0	0	0
ssb	0	0	0	1	0	0	0	0
tnpR	0	0	0	0	1	0	0	0
aldH	0	0	0	0	0	0	1	0
tetA	0	0	0	0	0	0	0	1
dnaB	0	0	0	0	0	0	0	0
intA	0	0	0	0	0	0	0	0
perB	0	0	0	0	0	0	0	0

eptA	1	0	0	0	0	1	0	0
merC	0	0	0	0	0	0	0	0
merA	0	0	0	0	0	0	0	0
atoC	0	0	0	0	0	1	0	0
hemN	0	0	0	0	0	0	0	1
dnaA	0	0	0	0	1	0	0	0
dapH	1	0	1	0	0	1	0	0
tuaG	1	0	1	0	0	1	0	0
gtfA	1	0	1	0	0	1	0	0
arnC	1	0	1	0	0	1	0	0
mdlY	0	1	0	0	1	0	0	0
exoX	0	1	0	0	0	0	0	0
emrE	0	1	0	0	0	0	0	1
emrE	0	0	0	0	0	0	0	1
thadh	0	1	0	0	1	0	0	0
ygcS	0	1	0	0	1	0	0	0
betL	0	1	0	0	1	0	0	0
fabB	0	1	0	0	1	0	0	0
fabG	0	1	0	0	1	0	0	0
ctcP	0	1	0	0	1	0	0	0
lolA	0	1	0	0	1	0	0	0
hutH	0	1	0	0	1	0	0	0
dltA	0	1	0	0	1	0	0	0
acpM	0	1	0	0	1	0	0	0
sotB	0	1	0	0	1	0	0	0
comR	0	1	0	0	1	0	0	0
pglH	0	1	0	1	0	0	0	0
mshA	0	1	0	1	0	0	0	0
lst	0	1	0	1	0	0	0	0
neuA	0	1	0	1	0	0	0	0
pglD	0	1	0	1	0	0	0	0
wbgU	0	0	1	0	0	0	0	0
rtcA	0	0	0	1	0	0	0	0
ompA	0	0	0	1	0	0	0	0
ltxB	0	0	0	1	0	0	0	0
prsE	0	0	0	1	0	0	0	0
yidH	0	0	0	1	0	0	0	0
rbn	0	0	0	1	0	0	0	0
mhpD	0	0	0	1	0	0	0	0
bphJ	0	0	0	1	0	0	0	0
bphI	0	0	0	1	0	0	0	0
todF	0	0	0	1	0	0	0	0
hcaE	0	0	0	1	0	0	0	0
hcaF	0	0	0	1	0	0	0	0
hcaC	0	0	0	1	0	0	0	0
mhpB	0	0	0	1	0	0	0	0
hcaD	0	0	0	1	0	0	0	0
mhpT	0	0	0	1	0	0	0	0
cntE	0	0	0	1	0	0	0	0
hcaR	0	0	0	1	0	0	0	0

mhpA	0	0	0	1	0	0	0	0
nagI	0	0	0	1	0	0	0	0
nagX	0	0	0	1	0	0	0	0
nagL	0	0	0	1	0	0	0	0
nagK	0	0	0	1	0	0	0	0
nicP	0	0	0	1	0	0	0	0
hcaB	0	0	0	1	0	0	0	0
pcaI	0	0	0	1	0	0	0	0
cmtAd	0	0	0	1	0	0	0	0
chqB	0	0	0	1	0	0	0	0
linF	0	0	0	1	0	0	0	0
bla	0	0	0	0	1	0	0	0
cysH	0	0	0	0	1	0	1	1
pglD	0	0	0	0	0	0	1	0
kptA	0	0	0	0	0	0	1	0
ywrO	0	0	0	0	0	0	1	0
cmlA5	0	0	0	0	0	0	0	1
dhfrI	0	0	0	0	0	0	0	1
wecD	0	0	0	0	0	0	0	1
tnsE	0	0	0	0	0	0	0	1
tnsC	0	0	0	0	0	0	0	1
tnsA	0	0	0	0	0	0	0	1
pcaF	0	0	0	1	0	0	0	0
smvA	1	0	0	1	0	1	0	0
czcO	0	0	0	0	0	0	0	0
arsC	0	0	0	0	0	0	0	0
zitB	0	0	0	0	0	0	0	0
cusA	0	0	0	0	0	0	0	0
mdtA	0	0	0	0	0	0	0	0
bauA	0	0	0	0	0	0	0	0
puuB	0	0	0	0	0	0	0	0
aldA	0	0	0	0	0	0	0	0
bicA	0	1	0	0	0	0	0	0
dauA	0	1	0	0	1	0	0	0
group	0	0	0	0	0	0	0	0
bicA	0	0	0	0	1	0	0	1
intS	0	0	0	0	0	0	0	0
cdiI	0	0	0	0	0	0	0	0
yybR	0	0	0	0	0	0	0	0
mhbT	0	0	0	0	1	1	0	0
oprB	0	0	0	0	0	0	0	0
rfbD	0	0	0	0	0	0	0	0
mopR	0	0	1	1	0	0	0	0
menE	1	0	0	0	0	0	0	0
ptsP	0	0	1	0	0	1	0	0
hcaD	0	0	0	1	0	0	0	0
ureC	0	0	1	0	0	1	0	0
recX	1	0	0	0	0	1	0	0
fhuE	0	0	0	0	0	0	0	0
tamB	0	0	1	0	0	0	0	0

tcyP	0	0	0	0	1	0	0	0
ttgA	1	0	0	0	0	1	0	0
typA	1	0	1	0	0	0	0	0
murE	1	0	0	0	0	0	0	0
gabR	1	0	1	0	0	0	0	0
umuC	0	0	0	0	0	0	0	0
folP	0	0	0	0	0	0	0	1
nahG	0	0	0	1	0	0	0	0
rssB	0	0	0	0	0	0	0	0
smvA	0	0	0	0	0	0	0	0
acrE	0	0	0	0	0	0	0	0
rclR	0	0	0	0	0	0	0	0
gpFI	0	0	0	1	0	0	0	0
exoX	0	0	0	1	0	0	0	0
intQ	0	0	0	1	0	0	0	0
garK	0	0	0	0	0	0	0	0
icaB	0	0	0	1	0	0	0	0
sldA	0	0	0	1	0	0	0	0
wbpE	0	0	0	0	1	0	0	0
wbpD	0	0	0	0	1	0	0	0
aacC1	0	0	0	0	0	0	0	1
kefF	0	0	0	0	0	0	0	0
glmM	0	0	0	0	0	0	0	1
cntO	0	0	0	0	0	0	0	0
dnaB	0	0	1	0	0	0	0	0
citN	0	0	0	0	0	0	0	0
cph2	0	0	0	0	0	0	1	0
dapF	1	0	0	0	0	1	0	0
creB	0	0	0	1	0	0	0	0
creC	0	0	0	1	0	0	0	0
creD	0	0	0	1	0	0	0	0
accA1	0	0	0	1	0	0	0	0
genK	0	0	0	1	0	0	0	0
pcpR	0	0	0	1	0	0	0	0
aqdA1	0	0	0	1	0	0	0	0
pcaK	0	0	0	1	0	0	0	0
yqjF	0	0	0	1	0	0	0	0
nepI	0	0	0	1	0	0	0	0
uvrB	0	0	0	1	0	0	0	0
dpnM	0	0	0	0	1	0	0	0
wbbD	0	0	0	0	1	0	0	0
wbpB	0	0	0	0	1	0	0	0
legF	0	0	0	0	0	0	1	0
pglD	0	0	0	0	0	0	1	0
pglF	0	0	0	0	0	0	1	0
copA	0	0	0	0	0	0	1	0
groS	0	0	0	0	0	0	1	0
pcaR	0	0	0	0	0	0	1	0
nudC	0	0	0	0	0	0	1	0
rutF	0	0	0	0	0	0	1	0

hpaIIM	0	0	0	0	0	0	1	0
vsr	0	0	0	0	0	0	1	0
ttr	0	0	0	0	0	0	1	0
rep	0	0	0	0	0	0	1	0
yfmJ	0	0	0	0	0	0	1	0
auaH	0	0	0	0	0	0	1	0
ephA	0	0	0	0	0	0	1	0
fosA	0	0	0	0	0	0	1	0
ydiO	0	0	0	0	0	0	0	1
noc	0	0	0	0	0	0	0	1
soj	0	0	0	0	0	0	0	1
xerC	0	0	0	0	0	0	0	1
dhfrI	0	0	0	0	0	0	0	0
wecD	0	0	0	0	0	0	0	0
ant1	0	0	0	0	0	0	0	0
ant1	0	0	0	0	0	0	0	1
fimA	0	0	0	0	0	0	0	1
clpP	0	0	0	0	0	0	0	1
dddP	0	0	0	0	0	0	0	1
betS	0	0	0	0	0	0	0	1
pld1	0	0	0	0	0	0	0	1
adc	0	0	0	0	0	0	0	1
uvrB	0	0	0	0	0	0	0	1
pcrA	0	0	0	0	0	0	0	1
recF	0	0	0	0	0	0	0	1
cspG	0	0	0	0	0	0	0	1
yafP	0	0	0	0	0	0	0	1
hin	0	0	0	0	0	0	0	1
dsbD	0	0	0	0	0	0	0	0
dtd3	0	0	0	0	0	0	0	0
hemA	0	0	0	0	0	0	0	0
asnO	0	0	0	0	0	0	0	0
hsdS	0	0	0	0	0	0	0	0
rapA	0	0	0	0	0	0	0	0
acp	0	0	0	0	0	0	0	0
btuB	0	0	0	0	0	0	0	0
wecB	0	0	0	0	0	0	0	0
tfpA	0	0	0	0	0	0	0	0
rhaS	0	0	0	0	0	0	0	0
elbB	0	0	0	0	0	0	0	0
folP	0	0	0	1	0	0	1	0
fusA	0	0	0	0	0	0	0	0
rpsG	0	0	0	0	0	0	0	0
caiT	0	0	0	0	0	0	0	0
tufA	0	0	0	0	0	0	0	0
rpsL	0	0	0	0	0	0	0	0
fabB	0	0	0	0	0	0	0	0
gabD	0	0	0	0	0	0	0	0
napA	0	0	0	0	0	0	0	0
aaeB	0	0	0	1	0	0	0	0

leuC1	0	0	0	0	0	1	0	0
acnA	0	0	0	1	0	0	0	0
choD	0	0	0	1	0	0	0	0
mdtD	0	0	1	0	0	0	0	0
purH	0	0	1	0	0	0	0	0
exoX	0	0	0	0	0	0	0	0
cas3	0	0	0	1	0	0	0	0
murD	0	0	0	1	0	0	0	0
recB	0	0	0	1	0	0	0	0
kefB	0	0	0	1	0	0	0	0
dmdD	0	0	0	1	0	0	0	0
rlmD	0	0	0	1	0	0	0	0
rapZ	0	1	0	0	0	0	0	0
pfeA	0	0	0	0	0	0	0	0
pqqE	0	0	0	1	0	0	0	0
ssuD	0	0	0	1	0	0	0	0
lpxA	1	0	0	0	0	0	0	0
mobA	0	0	0	0	0	0	0	0
gluQ	0	0	0	1	0	0	0	0
acnB	0	0	0	0	0	1	0	0
mmgC	0	0	0	0	0	0	0	0
entE	0	0	0	0	1	0	0	0
degP	0	0	0	0	0	0	0	0
cheB	0	0	0	0	0	0	0	0
craA	0	0	0	0	0	0	0	0
katE	1	0	0	0	0	0	0	0
ugd	0	0	1	0	0	0	0	0
czcA	0	0	0	0	0	1	0	0
umuC	0	1	0	0	0	0	0	0
aphA	0	0	0	0	0	1	0	0
csy2	0	0	0	1	0	0	0	0
nahG	0	0	0	1	0	0	0	0
upaG	0	0	0	0	0	0	0	0
srrA	0	0	0	0	0	0	0	0
smvA	0	0	0	0	0	0	0	0
acrE	0	0	0	0	0	0	0	0
nlhH	0	0	0	0	0	0	0	0
styD	0	0	0	0	0	0	0	0
arnT	0	0	1	0	0	0	0	0
tnpR	0	0	0	0	0	0	0	0
intA	0	0	0	0	0	0	0	0
bshA	0	0	0	0	0	0	0	0
aadB	0	0	0	0	0	0	0	0
xseA	0	0	0	0	0	0	0	0
dnaB	0	0	1	0	0	0	0	0
cydA	0	0	0	0	0	0	0	0
fhuA	0	0	0	0	0	0	0	0
pupA	0	0	0	0	0	0	0	0
fecR	0	0	0	0	0	0	0	0
fecI	0	0	0	0	0	0	0	0

ppnP	0	0	0	0	0	0	0	0
dapA	0	0	0	0	0	0	0	0
pycA	0	0	0	0	0	0	0	0
yddG	0	0	0	0	0	0	0	0
lcdH	0	0	0	0	0	0	0	0
ssuB	0	0	0	0	0	0	0	0
tolQ	0	0	0	0	0	0	0	0
exbD	0	0	0	0	0	0	0	0
atsK	0	0	0	0	0	0	0	0
ssuA	0	0	0	0	0	0	0	0
fyuA	0	0	0	0	0	0	0	0
ntaA	0	0	0	0	0	0	0	0
gpr	0	0	0	0	0	0	0	0
atsA	0	0	0	0	0	0	0	0
sasA	0	0	0	0	0	0	0	0
acrE	0	0	0	0	0	0	0	0
betI	0	0	0	0	0	0	0	0
pgrR	0	0	0	0	0	0	0	0
recD	0	0	0	0	0	0	0	0
relE	0	0	0	0	0	0	0	0
hin	0	0	0	0	0	0	0	0
yeeF	0	0	0	0	0	0	0	0
cntO	0	0	0	0	0	0	0	0
htrL	0	0	0	0	0	0	0	0
atpF	1	0	0	0	0	0	0	0
rsmC	1	0	0	0	0	0	0	0
mdtB	1	0	0	0	0	0	0	0
kdpD	0	0	0	0	0	1	0	0
benC	1	0	0	0	0	0	0	0
cat	0	1	0	0	0	0	0	0
aacA4	0	1	0	0	0	0	0	0
hisZ	0	0	1	0	0	0	0	0
QRSL1	0	0	1	0	0	0	0	0
tarM	0	0	0	1	0	0	0	0
rsuA	0	0	0	1	0	0	0	0
hcaB	0	0	0	1	0	0	0	0
pcaK	0	0	0	1	0	0	0	0
prmC	0	0	0	1	0	0	0	0
bioD	0	0	0	1	0	0	0	0
gabR	0	0	0	0	1	0	0	0
bla	0	0	0	0	0	0	0	0
quiA	0	0	0	0	1	0	0	0
eda	0	0	0	0	0	1	0	0
yceF	0	0	0	0	0	1	0	0
kdpB	0	0	0	0	0	1	0	0
soj	0	0	0	0	0	0	1	0
arsM	0	0	0	0	0	0	1	0
aqpZ2	0	0	0	0	0	0	1	0
hcaR	0	0	0	0	0	0	1	0
traG	0	0	0	0	0	0	1	0

virB4	0	0	0	0	0	0	1	0
folA	0	0	0	0	0	0	0	1
thyA	0	0	0	0	0	0	0	1
xerC	0	0	0	0	0	0	0	0
dhfrI	0	0	0	0	0	0	0	1
wecD	0	0	0	0	0	0	0	0
tnsE	0	0	0	0	0	0	0	0
tnsC	0	0	0	0	0	0	0	1
tnsB	0	0	0	0	0	0	0	0
tnsB	0	0	0	0	0	0	0	1
tnsA	0	0	0	0	0	0	0	1
quiC	0	0	0	1	0	0	0	0
aroE	0	0	0	1	0	0	0	0
lexA	0	0	0	0	0	0	0	0
dnaA	0	0	0	0	0	0	0	0
prtR	0	0	0	0	0	0	0	0
opuD	0	0	0	0	0	0	0	0
hpbD	0	0	0	0	0	0	0	0
mtaD	0	0	0	0	0	0	0	0
smtA	0	0	0	0	0	0	0	0
liuE	0	0	0	0	0	0	0	0
iclR	0	0	0	0	0	0	0	0
chrB1	0	0	0	0	0	0	0	0
chrA1	0	0	0	0	0	0	0	0
rcnR	0	0	0	0	0	0	0	0
czcB	0	0	0	0	0	0	0	0
yjiE	0	0	0	0	0	0	0	0
yddG	0	0	0	0	0	0	0	0
rutF	0	0	0	0	0	0	0	0
dncV	0	0	0	0	0	0	0	0
capV	0	0	0	0	0	0	0	0
copA	0	0	0	0	0	0	0	0
curA	0	0	0	0	0	0	0	0
nemR	0	0	0	0	0	0	0	0
nemA	0	0	0	0	0	0	0	0
group	0	0	0	0	0	0	0	0
yqhD	0	0	0	0	0	0	0	0
frmB	0	0	0	0	0	0	0	0
frmA	0	0	0	0	0	0	0	0
frmR	0	0	0	0	0	0	0	0
pks5	0	0	0	0	0	0	0	0
ppsC	0	0	0	0	0	0	0	0
dmlR	0	0	0	0	0	0	0	0
rffG	0	0	0	0	0	0	0	0
rfbA	0	0	0	0	0	0	0	0
rfbC	0	0	0	0	0	0	0	0
btuB	0	0	0	0	0	0	0	0
gdx	0	0	0	0	0	0	0	0
amtB	0	0	0	0	0	0	0	0
gcvT	0	0	0	0	0	0	0	0

narL	0	0	0	0	0	0	0	0
yeaX	0	0	0	0	0	0	0	0
glnK	0	0	0	0	0	0	0	0
purU	0	0	0	0	0	0	0	0
folD	0	0	0	0	0	0	0	0
soxB	0	0	0	0	0	0	0	0
soxA	0	0	0	0	0	0	0	0
puuR	0	0	0	0	0	0	0	0
purF	0	0	0	0	0	0	0	0
fni	0	0	0	0	0	0	0	0
yfcA	0	0	0	0	0	0	0	0
rutC	0	0	0	0	0	0	0	0
gltC	0	0	0	0	0	0	0	0
argE	0	0	0	0	0	0	0	0
Hgd	0	0	0	0	0	0	0	0
rutG	0	0	0	0	0	0	0	0
rutB	0	0	0	0	0	0	0	0
rutA	0	0	0	0	0	0	0	0
rutE	0	0	0	0	0	0	0	0
tnsE	0	0	0	0	0	0	0	0
tnsC	0	0	0	0	0	0	0	0
tnsA	0	0	0	0	0	0	0	0
acrF	0	0	0	0	0	0	0	0
pseI	0	0	0	0	0	0	0	0
pseG	0	0	0	0	0	0	0	0
pseC	0	0	0	0	0	0	0	0
pseB	0	0	0	0	0	0	0	0
pphB	0	0	0	0	0	0	0	0
dnaC	0	0	0	0	0	0	0	0
hsdR	0	0	0	0	0	0	0	0
dgkA	0	0	0	0	0	0	0	0
eptA	0	0	0	0	0	0	0	0
xseA	0	0	0	0	0	0	0	0
ppa	0	0	0	0	0	0	0	0
crcB	0	0	0	0	0	0	0	0
bicA	0	0	0	0	0	0	0	0
hicB	0	0	0	0	0	0	0	0
rsrIM	0	0	0	0	0	0	0	0
epsO	0	0	0	0	0	0	0	0
arnC	0	0	0	0	0	0	0	0
oatA	0	0	0	0	0	0	0	0
dnaB	0	0	0	0	0	0	0	0
smc	0	0	0	0	0	0	0	0
umuD	0	0	0	0	0	0	0	0
betT1	0	0	0	0	0	0	0	0
linX	0	0	0	0	0	0	0	0
iolG	0	0	0	0	0	0	0	0
perA	0	0	0	0	0	0	0	0
ytcD	0	0	0	0	0	0	0	0
qorA	0	0	0	0	0	0	0	0

mdlD	0	0	0	0	0	1	0	0
trxB	0	0	0	0	0	0	0	0
ywnA	0	0	0	0	0	0	0	0
pknD	0	0	0	0	0	0	0	0
recE	0	0	1	0	0	0	0	0
glnP	0	0	0	0	0	0	0	0
mrdA	0	0	0	0	0	0	0	0
thrS	0	0	0	0	0	0	0	0
pyrC	0	0	0	0	0	0	0	0
ccmM	0	0	0	0	0	0	0	0
hisI	0	0	0	0	0	0	0	0
folE2	0	0	0	0	0	0	0	0
fur	0	0	0	0	0	0	0	0
cysS	0	0	0	0	0	0	0	0
yciC	0	0	0	0	0	0	0	0
bigR	0	0	0	0	0	0	0	0
glpE	0	0	0	0	0	0	0	0
linC	0	0	0	0	0	0	0	0
cydA	0	0	0	0	0	0	0	0
norM	0	0	0	0	0	0	0	0
apxIB	0	0	0	0	0	0	0	0
cvaA	0	0	0	0	0	0	0	0
ahpF	0	0	0	0	0	0	0	0
surA	0	0	0	0	0	0	0	0
repA	0	0	0	0	0	0	0	0
tufB	0	0	0	0	0	0	0	0
fis	0	0	0	0	0	0	0	0
xcpX	0	0	0	1	0	0	0	0
prsA	0	0	0	0	0	1	0	0
tri1	0	0	0	0	0	0	0	0
katB	0	0	0	0	0	0	0	0
higA-2	0	0	0	0	0	0	0	0
higB-2	0	0	0	0	0	0	0	0
cspE	0	0	0	0	0	0	0	0
higB2	0	0	0	0	0	0	0	0
esiB	0	0	0	0	0	0	0	0
ohrB	0	0	0	0	0	0	0	0
ohrR	0	0	0	0	0	0	0	0
ant1	0	0	0	0	0	0	0	0
pse1	0	0	0	0	0	0	0	0
ere(B)	0	0	0	0	0	0	0	0
cntA	0	0	0	0	0	0	0	0
yciV	0	0	0	0	0	0	0	0
coaD	0	0	0	0	0	0	0	0
ppc	1	0	0	0	0	0	0	0
ppc	0	0	1	0	0	0	0	0
dmlA	0	0	0	0	0	0	0	0
nadE	0	0	0	0	0	0	0	0

AB30	AC1633	AF-401	AR_0083	AR_0101	DETAB-E51	JUNP543	MRSN1531	Nord4-2
1	1	1	1	1	1	1	1	1
1	1	1	1	1	1	1	1	1
1	1	1	1	1	1	1	1	1
1	1	1	1	1	1	1	1	1
1	1	1	1	1	1	1	1	1
1	1	1	1	1	1	1	1	1
1	1	1	1	1	1	1	1	1
1	1	1	1	1	1	1	1	1
1	1	1	1	1	1	1	1	1
1	1	1	1	1	1	1	1	1
1	1	1	1	1	1	1	1	1
1	1	1	1	1	1	1	1	1
1	1	1	1	1	1	1	1	1
1	1	1	1	1	1	1	1	1
1	1	1	1	1	1	1	1	1
1	1	1	1	1	1	1	1	1
1	1	1	1	1	1	1	1	1
1	1	1	1	1	1	1	1	1
1	1	1	1	1	1	1	1	1
1	1	1	1	1	1	1	1	1
1	1	1	1	1	1	1	1	1
1	1	1	1	1	1	1	1	1
1	1	1	1	1	1	1	1	1
1	1	1	1	1	1	1	1	1
1	1	1	1	1	1	1	1	1
1	1	1	1	1	1	1	1	1
1	1	1	1	1	1	1	1	1
1	1	1	1	1	1	1	1	1
1	1	1	1	1	1	1	1	1
1	1	1	1	1	1	1	1	1
1	1	1	1	1	1	1	1	1
1	1	1	1	1	1	1	1	1
1	1	1	1	1	1	1	1	1
1	1	1	1	1	1	1	1	1
1	1	1	1	1	1	1	1	1
1	1	1	1	1	1	1	1	1
1	1	1	1	1	1	1	1	1
1	1	1	1	1	1	1	1	1
1	1	1	1	1	1	1	1	1
1	1	1	1	1	1	1	1	1
1	1	1	1	1	1	1	1	1
1	1	1	1	1	1	1	1	1
1	1	1	1	1	1	1	1	1
1	1	1	1	1	1	1	1	1
1	1	1	1	1	1	1	1	1
1	1	1	1	1	1	1	1	1
1	1	1	1	1	1	1	1	1
1	1	1	1	1	1	1	1	1

1	1	1	1	1	1	1	1	1
1	1	1	1	1	1	1	1	1
1	1	1	1	1	1	1	1	1
1	1	1	1	1	1	1	1	1
1	1	1	1	1	1	1	1	1
1	1	1	1	1	1	1	1	1
1	1	1	1	1	1	1	1	1
1	1	1	1	1	1	1	1	1
1	1	1	1	1	1	1	1	1
1	1	1	1	1	1	1	1	1
1	1	1	1	1	1	1	1	1
1	1	1	1	1	1	1	1	1
1	1	1	1	1	1	1	1	1
1	1	1	1	1	1	1	1	1
1	1	1	1	1	1	1	1	1
1	1	1	1	1	1	1	1	1
1	1	1	1	1	1	1	1	1
1	1	1	1	1	1	1	1	1
1	1	1	1	1	1	1	1	1
1	1	1	1	1	1	1	1	1
1	1	1	1	1	1	1	1	1
1	1	1	1	1	1	1	1	1
1	1	1	1	1	1	1	1	1
1	1	1	1	1	1	1	1	1
1	1	1	1	1	1	1	1	1
1	1	1	1	1	1	1	1	1
1	1	1	1	1	1	1	1	1
1	1	1	1	1	1	1	1	1
1	1	1	1	1	1	1	1	1
1	1	1	1	1	1	1	1	1
1	1	1	1	1	1	1	1	1
1	1	1	1	1	1	1	1	1
1	1	1	1	1	1	1	1	1
1	1	1	1	1	1	1	1	1
1	1	1	1	1	1	1	1	1
1	1	1	1	1	1	1	1	1
1	1	1	1	1	1	1	1	1
1	1	1	1	1	1	1	1	1
1	1	1	1	1	1	1	1	1
1	1	1	1	1	1	1	1	1
1	1	1	1	1	1	1	1	1
1	1	1	1	1	1	1	1	1
1	1	1	1	1	1	1	1	1
1	1	1	1	1	1	1	1	1
1	1	1	1	1	1	1	1	1
1	1	1	1	1	1	1	1	1
1	1	1	1	1	1	1	1	1
1	1	1	1	1	1	1	1	1
1	1	1	1	1	1	1	1	1
1	1	1	1	1	1	1	1	1

1	1	1	1	1	1	1	1	1
1	1	1	1	1	1	1	1	1
1	1	1	1	1	1	1	1	1
1	1	1	1	1	1	1	1	1
1	1	1	1	1	1	1	1	1
1	1	1	1	1	1	1	1	1
1	1	1	1	1	1	1	1	1
1	1	1	1	1	1	1	1	1
1	1	1	1	1	1	1	1	1
1	1	1	1	1	1	1	1	1
1	1	1	1	1	1	1	1	1
1	1	1	1	1	1	1	1	1
1	1	1	1	1	1	1	1	1
1	1	1	1	1	1	1	1	1
1	1	1	1	1	1	1	1	1
1	1	1	1	1	1	1	1	1
1	1	1	1	1	1	1	1	1
1	1	1	1	1	1	1	1	1
1	1	1	1	1	1	1	1	1
1	1	1	1	1	1	1	1	1
1	1	1	1	1	1	1	1	1
1	1	1	1	1	1	1	1	1
1	1	1	1	1	1	1	1	1
1	1	1	1	1	1	1	1	1
1	1	1	1	1	1	1	1	1
1	1	1	1	1	1	1	1	1
1	1	1	1	1	1	1	1	1
1	1	1	1	1	1	1	1	1
1	1	1	1	1	1	1	1	1
1	1	1	1	1	1	1	1	1
1	1	1	1	1	1	1	1	1
1	1	1	1	1	1	1	1	1
1	1	1	1	1	1	1	1	1
1	1	1	1	1	1	1	1	1
1	1	1	1	1	1	1	1	1
1	1	1	1	1	1	1	1	1
1	1	1	1	1	1	1	1	1
1	1	1	1	1	1	1	1	1
1	1	1	1	1	1	1	1	1
1	1	1	1	1	1	1	1	1
1	1	1	1	1	1	1	1	1
1	1	1	1	1	1	1	1	1
1	1	1	1	1	1	1	1	1
1	1	1	1	1	1	1	1	1
1	1	1	1	1	1	1	1	1
1	1	1	1	1	1	1	1	1
1	1	1	1	1	1	1	1	1
1	1	1	1	1	1	1	1	1
1	1	1	1	1	1	1	1	1
1	1	1	1	1	1	1	1	1

1	1	1	1	1	1	1	1	1
1	1	1	1	1	1	1	1	1
1	1	1	1	1	1	1	1	1
1	1	1	1	1	1	1	1	1
1	1	1	1	1	1	1	1	1
1	1	1	1	1	1	1	1	1
1	1	1	1	1	1	1	1	1
1	1	1	1	1	1	1	1	1
1	1	1	1	1	1	1	1	1
1	1	1	1	1	1	1	1	1
1	1	1	1	1	1	1	1	1
1	1	1	1	1	1	1	1	1
1	1	1	1	1	1	1	1	1
1	1	1	1	1	1	1	1	1
1	1	1	1	1	1	1	1	1
1	1	1	1	1	1	1	1	1
1	1	1	1	1	1	1	1	1
1	1	1	1	1	1	1	1	1
1	1	1	1	1	1	1	1	1
1	1	1	1	1	1	1	1	1
1	1	1	1	1	1	1	1	1
1	1	1	1	1	1	1	1	1
1	1	1	1	1	1	1	1	1
1	1	1	1	1	1	1	1	1
1	1	1	1	1	1	1	1	1
1	1	1	1	1	1	1	1	1
1	1	1	1	1	1	1	1	1
1	1	1	1	1	1	1	1	1
1	1	1	1	1	1	1	1	1
1	1	1	1	1	1	1	1	1
1	1	1	1	1	1	1	1	1
1	1	1	1	1	1	1	1	1
1	1	1	1	1	1	1	1	1
1	1	1	1	1	1	1	1	1
1	1	1	1	1	1	1	1	1
1	1	1	1	1	1	1	1	1
1	1	1	1	1	1	1	1	1
1	1	1	1	1	1	1	1	1
1	1	1	1	1	1	1	1	1
1	1	1	1	1	1	1	1	1
1	1	1	1	1	1	1	1	1
1	1	1	1	1	1	1	1	1
1	1	1	1	1	1	1	1	1
1	1	1	1	1	1	1	1	1
1	1	1	1	1	1	1	1	1
1	1	1	1	1	1	1	1	1
1	1	1	1	1	1	1	1	1
1	1	1	1	1	1	1	1	1
1	1	1	1	1	1	1	1	1
1	1	1	1	1	1	1	1	1

1	1	1	1	1	1	1	1	1
1	1	1	1	1	1	1	1	1
1	1	1	1	1	1	1	1	1
1	1	1	1	1	1	1	1	1
1	1	1	1	1	1	1	1	1
1	1	1	1	1	1	1	1	1
1	1	1	1	1	1	1	1	1
1	1	1	1	1	1	1	1	1
1	1	1	1	1	1	1	1	1
1	1	1	1	1	1	1	1	1
1	1	1	1	1	1	1	1	1
1	1	1	1	1	1	1	1	1
1	1	1	1	1	1	1	1	1
1	1	1	1	1	1	1	1	1
1	1	1	1	1	1	1	1	1
1	1	1	1	1	1	1	1	1
1	1	1	1	1	1	1	1	1
1	1	1	1	1	1	1	1	1
1	1	1	1	1	1	1	1	1
1	1	1	1	1	1	1	1	1
1	1	1	1	1	1	1	1	1
1	1	1	1	1	1	1	1	1
1	1	1	1	1	1	1	1	1
1	1	1	1	1	1	1	1	1
1	1	1	1	1	1	1	1	1
1	1	1	1	1	1	1	1	1
1	1	1	1	1	1	1	1	1
1	1	1	1	1	1	1	1	1
1	1	1	1	1	1	1	1	1
1	1	1	1	1	1	1	1	1
1	1	1	1	1	1	1	1	1
1	1	1	1	1	1	1	1	1
1	1	1	1	1	1	1	1	1
1	1	1	1	1	1	1	1	1
1	1	1	1	1	1	1	1	1
1	1	1	1	1	1	1	1	1
1	1	1	1	1	1	1	1	1
1	1	1	1	1	1	1	1	1
1	1	1	1	1	1	1	1	1
1	1	1	1	1	1	1	1	1
1	1	1	1	1	1	1	1	1
1	1	1	1	1	1	1	1	1
1	1	1	1	1	1	1	1	1
1	1	1	1	1	1	1	1	1
1	1	1	1	1	1	1	1	1
1	1	1	1	1	1	1	1	1
1	1	1	1	1	1	1	1	1
1	1	1	1	1	1	1	1	1
1	1	1	1	1	1	1	1	1
1	1	1	1	1	1	1	1	1

1	1	1	1	1	1	1	1	1
1	1	1	1	1	1	1	1	1
1	1	1	1	1	1	1	1	1
1	1	1	1	1	1	1	1	1
1	1	1	1	1	1	1	1	1
1	1	1	1	1	1	1	1	1
1	1	1	1	1	1	1	1	1
1	1	1	1	1	1	1	1	1
1	1	1	1	1	1	1	1	1
1	1	1	1	1	1	1	1	1
1	1	1	1	1	1	1	1	1
1	1	1	1	1	1	1	1	1
1	1	1	1	1	1	1	1	1
1	1	1	1	1	1	1	1	1
1	1	1	1	1	1	1	1	1
1	1	1	1	1	1	1	1	1
1	1	1	1	1	1	1	1	1
1	1	1	1	1	1	1	1	1
1	1	1	1	1	1	1	1	1
1	1	1	1	1	1	1	1	1
1	1	1	1	1	1	1	1	1
1	1	1	1	1	1	1	1	1
1	1	1	1	1	1	1	1	1
1	1	1	1	1	1	1	1	1
1	1	1	1	1	1	1	1	1
1	1	1	1	1	1	1	1	1
1	1	1	1	1	1	1	1	1
1	1	1	1	1	1	1	1	1
1	1	1	1	1	1	1	1	1
1	1	1	1	1	1	1	1	1
1	1	1	1	1	1	1	1	1
1	1	1	1	1	1	1	1	1
1	1	1	1	1	1	1	1	1
1	1	1	1	1	1	1	1	1
1	1	1	1	1	1	1	1	1
1	1	1	1	1	1	1	1	1
1	1	1	1	1	1	1	1	1
1	1	1	1	1	1	1	1	1
1	1	1	1	1	1	1	1	1
1	1	1	1	1	1	1	1	1
1	1	1	1	1	1	1	1	1
1	1	1	1	1	1	1	1	1
1	1	1	1	1	1	1	1	1
1	1	1	1	1	1	1	1	1
1	1	1	1	1	1	1	1	1
1	1	1	1	1	1	1	1	1
1	1	1	1	1	1	1	1	1
1	1	1	1	1	1	1	1	1
1	1	1	1	1	1	1	1	1
1	1	1	1	1	1	1	1	1

1	1	1	1	1	1	1	1	1
1	1	1	1	1	1	1	1	1
1	1	1	1	1	1	1	1	1
1	1	1	1	1	1	1	1	1
1	1	1	1	1	1	1	1	1
1	1	1	1	1	1	1	1	1
1	1	1	1	1	1	1	1	1
1	1	1	1	1	1	1	1	1
1	1	1	1	1	1	1	1	1
1	1	1	1	1	1	1	1	1
1	1	1	1	1	1	1	1	1
1	1	1	1	1	1	1	1	1
1	1	1	1	1	1	1	1	1
1	1	1	1	1	1	1	1	1
1	1	1	1	1	1	1	1	1
1	1	1	1	1	1	1	1	1
1	1	1	1	1	1	1	1	1
1	1	1	1	1	1	1	1	1
1	1	1	1	1	1	1	1	1
1	1	1	1	1	1	1	1	1
1	1	1	1	1	1	1	1	1
1	1	1	1	1	1	1	1	1
1	1	1	1	1	1	1	1	1
1	1	1	1	1	1	1	1	1
1	1	1	1	1	1	1	1	1
1	1	1	1	1	1	1	1	1
1	1	1	1	1	1	1	1	1
1	1	1	1	1	1	1	1	1
1	1	1	1	1	1	1	1	1
1	1	1	1	1	1	1	1	1
1	1	1	1	1	1	1	1	1
1	1	1	1	1	1	1	1	1
1	1	1	1	1	1	1	1	1
1	1	1	1	1	1	1	1	1
1	1	1	1	1	1	1	1	1
1	1	1	1	1	1	1	1	1
1	1	1	1	1	1	1	1	1
1	1	1	1	1	1	1	1	1
1	1	1	1	1	1	1	1	1
1	1	1	1	1	1	1	1	1
1	1	1	1	1	1	1	1	1
1	1	1	1	1	1	1	1	1
1	1	1	1	1	1	1	1	1
1	1	1	1	1	1	1	1	1
1	1	1	1	1	1	1	1	1
1	1	1	1	1	1	1	1	1
1	1	1	1	1	1	1	1	1
1	1	1	1	1	1	1	1	1
1	1	1	1	1	1	1	1	1
1	1	1	1	1	1	1	1	1

1	1	1	1	1	1	1	1	1
1	1	1	1	1	1	1	1	1
1	1	1	1	1	1	1	1	1
1	1	1	1	1	1	1	1	1
1	1	1	1	1	1	1	1	1
1	1	1	1	1	1	1	1	1
1	1	1	1	1	1	1	1	1
1	1	1	1	1	1	1	1	1
1	1	1	1	1	1	1	1	1
1	1	1	1	1	1	1	1	1
1	1	1	1	1	1	1	1	1
1	1	1	1	1	1	1	1	1
1	1	1	1	1	1	1	1	1
1	1	1	1	1	1	1	1	1
1	1	1	1	1	1	1	1	1
1	1	1	1	1	1	1	1	1
1	1	1	1	1	1	1	1	1
1	1	1	1	1	1	1	1	1
1	1	1	1	1	1	1	1	1
1	1	1	1	1	1	1	1	1
1	1	1	1	1	1	1	1	1
1	1	1	1	1	1	1	1	1
1	1	1	1	1	1	1	1	1
1	1	1	1	1	1	1	1	1
1	1	1	1	1	1	1	1	1
1	1	1	1	1	1	1	1	1
1	1	1	1	1	1	1	1	1
1	1	1	1	1	1	1	1	1
1	1	1	1	1	1	1	1	1
1	1	1	1	1	1	1	1	1
1	1	1	1	1	1	1	1	1
1	1	1	1	1	1	1	1	1
1	1	1	1	1	1	1	1	1
1	1	1	1	1	1	1	1	1
1	1	1	1	1	1	1	1	1
1	1	1	1	1	1	1	1	1
1	1	1	1	1	1	1	1	1
1	1	1	1	1	1	1	1	1
1	1	1	1	1	1	1	1	1
1	1	1	1	1	1	1	1	1
1	1	1	1	1	1	1	1	1
1	1	1	1	1	1	1	1	1
1	1	1	1	1	1	1	1	1
1	1	1	1	1	1	1	1	1
1	1	1	1	1	1	1	1	1
1	1	1	1	1	1	1	1	1
1	1	1	1	1	1	1	1	1
1	1	1	1	1	1	1	1	1
1	1	1	1	1	1	1	1	1
1	1	1	1	1	1	1	1	1

1	1	1	1	1	1	1	1	1
1	1	1	1	1	1	1	1	1
1	1	1	1	1	1	1	1	1
1	1	1	1	1	1	1	1	1
1	1	1	1	1	1	1	1	1
1	1	1	1	1	1	1	1	1
1	1	1	1	1	1	1	1	1
1	1	1	1	1	1	1	1	1
1	1	1	1	1	1	1	1	1
1	1	1	1	1	1	1	1	1
1	1	1	1	1	1	1	1	1
1	1	1	1	1	1	1	1	1
1	1	1	1	1	1	1	1	1
1	1	1	1	1	1	1	1	1
1	1	1	1	1	1	1	1	1
1	1	1	1	1	1	1	1	1
1	1	1	1	1	1	1	1	1
1	1	1	1	1	1	1	1	1
1	1	1	1	1	1	1	1	1
1	1	1	1	1	1	1	1	1
1	1	1	1	1	1	1	1	1
1	1	1	1	1	1	1	1	1
1	1	1	1	1	1	1	1	1
1	1	1	1	1	1	1	1	1
1	1	1	1	1	1	1	1	1
1	1	1	1	1	1	1	1	1
1	1	1	1	1	1	1	1	1
1	1	1	1	1	1	1	1	1
1	1	1	1	1	1	1	1	1
1	1	1	1	1	1	1	1	1
1	1	1	1	1	1	1	1	1
1	1	1	1	1	1	1	1	1
1	1	1	1	1	1	1	1	1
1	1	1	1	1	1	1	1	1
1	1	1	1	1	1	1	1	1
1	1	1	1	1	1	1	1	1
1	1	1	1	1	1	1	1	1
1	1	1	1	1	1	1	1	1
1	1	1	1	1	1	1	1	1
1	1	1	1	1	1	1	1	1
1	1	1	1	1	1	1	1	1
1	1	1	1	1	1	1	1	1
1	1	1	1	1	1	1	1	1
1	1	1	1	1	1	1	1	1
1	1	1	1	1	1	1	1	1
1	1	1	1	1	1	1	1	1
1	1	1	1	1	1	1	1	1
1	1	1	1	1	1	1	1	1
1	1	1	1	1	1	1	1	1
1	1	1	1	1	1	1	1	1

1	1	1	1	1	1	1	1	1
1	1	1	1	1	1	1	1	1
1	1	1	1	1	1	1	1	1
1	1	1	1	1	1	1	1	1
1	1	1	1	1	1	1	1	1
1	1	1	1	1	1	1	1	1
1	1	1	1	1	1	1	1	1
1	1	1	1	1	1	1	1	1
1	1	1	1	1	1	1	1	1
1	1	1	1	1	1	1	1	1
1	1	1	1	1	1	1	1	1
1	1	1	1	1	1	1	1	1
1	1	1	1	1	1	1	1	1
1	1	1	1	1	1	1	1	1
1	1	1	1	1	1	1	1	1
1	1	1	1	1	1	1	1	1
1	1	1	1	1	1	1	1	1
1	1	1	1	1	1	1	1	1
1	1	1	1	1	1	1	1	1
1	1	1	1	1	1	1	1	1
1	1	1	1	1	1	1	1	1
1	1	1	1	1	1	1	1	1
1	1	1	1	1	1	1	1	1
1	1	1	1	1	1	1	1	1
1	1	1	1	1	1	1	1	1
1	1	1	1	1	1	1	1	1
1	1	1	1	1	1	1	1	1
1	1	1	1	1	1	1	1	1
1	1	1	1	1	1	1	1	1
1	1	1	1	1	1	1	1	1
1	1	1	1	1	1	1	1	1
1	1	1	1	1	1	1	1	1
1	1	1	1	1	1	1	1	1
1	1	1	1	1	1	1	1	1
1	1	1	1	1	1	1	1	1
1	1	1	1	1	1	1	1	1
1	1	1	1	1	1	1	1	1
1	1	1	1	1	1	1	1	1
1	1	1	1	1	1	1	1	1
1	1	1	1	1	1	1	1	1
1	1	1	1	1	1	1	1	1
1	1	1	1	1	1	1	1	1
1	1	1	1	1	1	1	1	1
1	1	1	1	1	1	1	1	1
1	1	1	1	1	1	1	1	1
1	1	1	1	1	1	1	1	1
1	1	1	1	1	1	1	1	1
1	1	1	1	1	1	1	1	1
1	1	1	1	1	1	1	1	1
1	1	1	1	1	1	1	1	1

1	1	1	1	1	1	1	1	1
1	1	1	1	1	1	1	1	1
1	1	1	1	1	1	1	1	1
1	1	1	1	1	1	1	1	1
1	1	1	1	1	1	1	1	1
1	1	1	1	1	1	1	1	1
1	1	1	1	1	1	1	1	1
1	1	1	1	1	1	1	1	1
1	1	1	1	1	1	1	1	1
1	1	1	1	1	1	1	1	1
1	1	1	1	1	1	1	1	1
1	1	1	1	1	1	1	1	1
1	1	1	1	1	1	1	1	1
1	1	1	1	1	1	1	1	1
1	1	1	1	1	1	1	1	1
1	1	1	1	1	1	1	1	1
1	1	1	1	1	1	1	1	1
1	1	1	1	1	1	1	1	1
1	1	1	1	1	1	1	1	1
1	1	1	1	1	1	1	1	1
1	1	1	1	1	1	1	1	1
1	1	1	1	1	1	1	1	1
1	1	1	1	1	1	1	1	1
1	1	1	1	1	1	1	1	1
1	1	1	1	1	1	1	1	1
1	1	1	1	1	1	1	1	1
1	1	1	1	1	1	1	1	1
1	1	1	1	1	1	1	1	1
1	1	1	1	1	1	1	1	1
1	1	1	1	1	1	1	1	1
1	1	1	1	1	1	1	1	1
1	1	1	1	1	1	1	1	1
1	1	1	1	1	1	1	1	1
1	1	1	1	1	1	1	1	1
1	1	1	1	1	1	1	1	1
1	1	1	1	1	1	1	1	1
1	1	1	1	1	1	1	1	1
1	1	1	1	1	1	1	1	1
1	1	1	1	1	1	1	1	1
1	1	1	1	1	1	1	1	1
1	1	1	1	1	1	1	1	1
1	1	1	1	1	1	1	1	1
1	1	1	1	1	1	1	1	1
1	1	1	1	1	1	1	1	1
1	1	1	1	1	1	1	1	1
1	1	1	1	1	1	1	1	1
1	1	1	1	1	1	1	1	1
1	1	1	1	1	1	1	1	1
1	1	1	1	1	1	1	1	1
1	1	1	1	1	1	1	1	1

1	1	1	1	1	1	1	1	1
1	1	1	1	1	1	1	1	1
1	1	1	1	1	1	1	1	1
1	1	1	1	1	1	1	1	1
1	1	1	1	1	1	1	1	1
1	1	1	1	1	1	1	1	1
1	1	1	1	1	1	1	1	1
1	1	1	1	1	1	1	1	1
1	1	1	1	1	1	1	1	1
1	1	1	1	1	1	1	1	1
1	1	1	1	1	1	1	1	1
1	1	1	1	1	1	1	1	1
1	1	1	1	1	1	1	1	1
1	1	1	1	1	1	1	1	1
1	1	1	1	1	1	1	1	1
1	1	1	1	1	1	1	1	1
1	1	1	1	1	1	1	1	1
1	1	1	1	1	1	1	1	1
1	1	1	1	1	1	1	1	1
1	1	1	1	1	1	1	1	1
1	1	1	1	1	1	1	1	1
1	1	1	1	1	1	1	1	1
1	1	1	1	1	1	1	1	1
1	1	1	1	1	1	1	1	1
1	1	1	1	1	1	1	1	1
1	1	1	1	1	1	1	1	1
1	1	1	1	1	1	1	1	1
1	1	1	1	1	1	1	1	1
1	1	1	1	1	1	1	1	1
1	1	1	1	1	1	1	1	1
1	1	1	1	1	1	1	1	1
1	1	1	1	1	1	1	1	1
1	1	1	1	1	1	1	1	1
1	1	1	1	1	1	1	1	1
1	1	1	1	1	1	1	1	1
1	1	1	1	1	1	1	1	1
1	1	1	1	1	1	1	1	1
1	1	1	1	1	1	1	1	1
1	1	1	1	1	1	1	1	1
1	1	1	1	1	1	1	1	1
1	1	1	1	1	1	1	1	1
1	1	1	1	1	1	1	1	1
1	1	1	1	1	1	1	1	1
1	1	1	1	1	1	1	1	1
1	1	1	1	1	1	1	1	1
1	1	1	1	1	1	1	1	1
1	1	1	1	1	1	1	1	1
1	1	1	1	1	1	1	1	1
1	1	1	1	1	1	1	1	1
1	1	1	1	1	1	1	1	1

1	1	1	1	1	1	1	1	1
1	1	1	1	1	1	1	1	1
1	1	1	1	1	1	1	1	1
1	1	1	1	1	1	1	1	1
1	1	1	1	1	1	1	1	1
1	1	1	1	1	1	1	1	1
1	1	1	1	1	1	1	1	1
1	1	1	1	1	1	1	1	1
1	1	1	1	1	1	1	1	1
1	1	1	1	1	1	1	1	1
1	1	1	1	1	1	1	1	1
1	1	1	1	1	1	1	1	1
1	1	1	1	1	1	1	1	1
1	1	1	1	1	1	1	1	1
1	1	1	1	1	1	1	1	1
1	1	1	1	1	1	1	1	1
1	1	1	1	1	1	1	1	1
1	1	1	1	1	1	1	1	1
1	1	1	1	1	1	1	1	1
1	1	1	1	1	1	1	1	1
1	1	1	1	1	1	1	1	1
1	1	1	1	1	1	1	1	1
1	1	1	1	1	1	1	1	1
1	1	1	1	1	1	1	1	1
1	1	1	1	1	1	1	1	1
1	1	1	1	1	1	1	1	1
1	1	1	1	1	1	1	1	1
1	1	1	1	1	1	1	1	1
1	1	1	1	1	1	1	1	1
1	1	1	1	1	1	1	1	1
1	1	1	1	1	1	1	1	1
1	1	1	1	1	1	1	1	1
1	1	1	1	1	1	1	1	1
1	1	1	1	1	1	1	1	1
1	1	1	1	1	1	1	1	1
1	1	1	1	1	1	1	1	1
1	1	1	1	1	1	1	1	1
1	1	1	1	1	1	1	1	1
1	1	1	1	1	1	1	1	1
1	1	1	1	1	1	1	1	1
1	1	1	1	1	1	1	1	1
1	1	1	1	1	1	1	1	1
1	1	1	1	1	1	1	1	1
1	1	1	1	1	1	1	1	1
1	1	1	1	1	1	1	1	1
1	1	1	1	1	1	1	1	1
1	1	1	1	1	1	1	1	1
1	1	1	1	1	1	1	1	1
1	1	1	1	1	1	1	1	1
1	1	1	1	1	1	1	1	1

1	1	1	1	1	1	1	1	1
1	1	1	1	1	1	1	1	1
1	1	1	1	1	1	1	1	1
1	1	1	1	1	1	1	1	1
1	1	1	1	1	1	1	1	1
1	1	1	1	1	1	1	1	1
1	1	1	1	1	1	1	1	1
1	1	1	1	1	1	1	1	1
1	1	1	1	1	1	1	1	1
1	1	1	1	1	1	1	1	1
1	1	1	1	1	1	1	1	1
1	1	1	1	1	1	1	1	1
1	1	1	1	1	1	1	1	1
1	1	1	1	1	1	1	1	1
1	1	1	1	1	1	1	1	1
1	1	1	1	1	1	1	1	1
1	1	1	1	1	1	1	1	1
1	1	1	1	1	1	1	1	1
1	1	1	1	1	1	1	1	1
1	1	1	1	1	1	1	1	1
1	1	1	1	1	1	1	1	1
1	1	1	1	1	1	1	1	1
1	1	1	1	1	1	1	1	1
1	1	1	1	1	1	1	1	1
1	1	1	1	1	1	1	1	1
1	1	1	1	1	1	1	1	1
1	1	1	1	1	1	1	1	1
1	1	1	1	1	1	1	1	1
1	1	1	1	1	1	1	1	1
1	1	1	1	1	1	1	1	1
1	1	1	1	1	1	1	1	1
1	1	1	1	1	1	1	1	1
1	1	1	1	1	1	1	1	1
1	1	1	1	1	1	1	1	1
1	1	1	1	1	1	1	1	1
1	1	1	1	1	1	1	1	1
1	1	1	1	1	1	1	1	1
1	1	1	1	1	1	1	1	1
1	1	1	1	1	1	1	1	1
1	1	1	1	1	1	1	1	1
1	1	1	1	1	1	1	1	1
1	1	1	1	1	1	1	1	1
1	1	1	1	1	1	1	1	1
1	1	1	1	1	1	1	1	1
1	1	1	1	1	1	1	1	1
1	1	1	1	1	1	1	1	1
1	1	1	1	1	1	1	1	1
1	1	1	1	1	1	1	1	1
1	1	1	1	1	1	1	1	1
1	1	1	1	1	1	1	1	1

1	1	1	1	1	1	1	1	1
1	1	1	1	1	1	1	1	1
1	1	1	1	1	1	1	1	1
1	1	1	1	1	1	1	1	1
1	1	1	1	1	1	1	1	1
1	1	1	1	1	1	1	1	1
1	1	1	1	1	1	1	1	1
1	1	1	1	1	1	1	1	1
1	1	1	1	1	1	1	1	1
1	1	1	1	1	1	1	1	1
1	1	1	1	1	1	1	1	1
1	1	1	1	1	1	1	1	1
1	1	1	1	1	1	1	1	1
1	1	1	1	1	1	1	1	1
1	1	1	1	1	1	1	1	1
1	1	1	1	1	1	1	1	1
1	1	1	1	1	1	1	1	1
1	1	1	1	1	1	1	1	1
1	1	1	1	1	1	1	1	1
1	1	1	1	1	1	1	1	1
1	1	1	1	1	1	1	1	1
1	1	1	1	1	1	1	1	1
1	1	1	1	1	1	1	1	1
1	1	1	1	1	1	1	1	1
1	1	1	1	1	1	1	1	1
1	1	1	1	1	1	1	1	1
1	1	1	1	1	1	1	1	1
1	1	1	1	1	1	1	1	1
1	1	1	1	1	1	1	1	1
1	1	1	1	1	1	1	1	1
1	1	1	1	1	1	1	1	1
1	1	1	1	1	1	1	1	1
1	1	1	1	1	1	1	1	1
1	1	1	1	1	1	1	1	1
1	1	1	1	1	1	1	1	1
1	1	1	1	1	1	1	1	1
1	1	1	1	1	1	1	1	1
1	1	1	1	1	1	1	1	1
1	1	1	1	1	1	1	1	1
1	1	1	1	1	1	1	1	1
1	1	1	1	1	1	1	1	1
1	1	1	1	1	1	1	1	1
1	1	1	1	1	1	1	1	1
1	1	1	1	1	1	1	1	1
1	1	1	1	1	1	1	1	1
1	1	1	1	1	1	1	1	1
1	1	1	1	1	1	1	1	1
1	1	1	1	1	1	1	1	1
1	1	1	1	1	1	1	1	1
1	1	1	1	1	1	1	1	1

1	1	1	1	1	1	1	1	1
1	1	1	1	1	1	1	1	1
1	1	1	1	1	1	1	1	1
1	1	1	1	1	1	1	1	1
1	1	1	1	1	1	1	1	1
1	1	1	1	1	1	1	1	1
1	1	1	1	1	1	1	1	1
1	1	1	1	1	1	1	1	1
1	1	1	1	1	1	1	1	1
1	1	1	1	1	1	1	1	1
1	1	1	1	1	1	1	1	1
1	1	1	1	1	1	1	1	1
1	1	1	1	1	1	1	1	1
1	1	1	1	1	1	1	1	1
1	1	1	1	1	1	1	1	1
1	1	1	1	1	1	1	1	1
1	1	1	1	1	1	1	1	1
1	1	1	1	1	1	1	1	1
1	1	1	1	1	1	1	1	1
1	1	1	1	1	1	1	1	1
1	1	1	1	1	1	1	1	1
1	1	1	1	1	1	1	1	1
1	1	1	1	1	1	1	1	1
1	1	1	1	1	1	1	1	1
1	1	1	1	1	1	1	1	1
1	1	1	1	1	1	1	1	1
1	1	1	1	1	1	1	1	1
1	1	1	1	1	1	1	1	1
1	1	1	1	1	1	1	1	1
1	1	1	1	1	1	1	1	1
1	1	1	1	1	1	1	1	1
1	1	1	1	1	1	1	1	1
1	1	1	1	1	1	1	1	1
1	1	1	1	1	1	1	1	1
1	1	1	1	1	1	1	1	1
1	1	1	1	1	1	1	1	1
1	1	1	1	1	1	1	1	1
1	1	1	1	1	1	1	1	1
1	1	1	1	1	1	1	1	1
1	1	1	1	1	1	1	1	1
1	1	1	1	1	1	1	1	1
1	1	1	1	1	1	1	1	1
1	1	1	1	1	1	1	1	1
1	1	1	1	1	1	1	1	1
1	1	1	1	1	1	1	1	1
1	1	1	1	1	1	1	1	1
1	1	1	1	1	1	1	1	1
1	1	1	1	1	1	1	1	1
1	1	1	1	1	1	1	1	1
1	1	1	1	1	1	1	1	1

1	1	1	1	1	1	1	1	1
1	1	1	1	1	1	1	1	1
1	1	1	1	1	1	1	1	1
1	1	1	1	1	1	1	1	1
1	1	1	1	1	1	1	1	1
1	1	1	1	1	1	1	1	1
1	1	1	1	1	1	1	1	1
1	1	1	1	1	1	1	1	1
1	1	1	1	1	1	1	1	1
1	1	1	1	1	1	1	1	1
1	1	1	1	1	1	1	1	1
1	1	1	1	1	1	1	1	1
1	1	1	1	1	1	1	1	1
1	1	1	1	1	1	1	1	1
1	1	1	1	1	1	1	1	1
1	1	1	1	1	1	1	1	1
1	1	1	1	1	1	1	1	1
1	1	1	1	1	1	1	1	1
1	1	1	1	1	1	1	1	1
1	1	1	1	1	1	1	1	1
1	1	1	1	1	1	1	1	1
1	1	1	1	1	1	1	1	1
1	1	1	1	1	1	1	1	1
1	1	1	1	1	1	1	1	1
1	1	1	1	1	1	1	1	1
1	1	1	1	1	1	1	1	1
1	1	1	1	1	1	1	1	1
1	1	1	1	1	1	1	1	1
1	1	1	1	1	1	1	1	1
1	1	1	1	1	1	1	1	1
1	1	1	1	1	1	1	1	1
1	1	1	1	1	1	1	1	1
1	1	1	1	1	1	1	1	1
1	1	1	1	1	1	1	1	1
1	1	1	1	1	1	1	1	1
1	1	1	1	1	1	1	1	1
1	1	1	1	1	1	1	1	1
1	1	1	1	1	1	1	1	1
1	1	1	1	1	1	1	1	1
1	1	1	1	1	1	1	1	1
1	1	1	1	1	1	1	1	1
1	1	1	1	1	1	1	1	1
1	1	1	1	1	1	1	1	1
1	1	1	1	1	1	1	1	1
1	1	1	1	1	1	1	1	1
1	1	1	1	1	1	1	1	1
1	1	1	1	1	1	1	1	1
1	1	1	1	1	1	1	1	1
1	1	1	1	1	1	1	1	1
1	1	1	1	1	1	1	1	1

1	1	1	1	1	1	1	1	1
1	1	1	1	1	1	1	1	1
1	1	1	1	1	1	1	1	1
1	1	1	1	1	1	1	1	1
1	1	1	1	1	1	1	1	1
1	1	1	1	1	1	1	1	1
1	1	1	1	1	1	1	1	1
1	1	1	1	1	1	1	1	1
1	1	1	1	1	1	1	1	1
1	1	1	1	1	1	1	1	1
1	1	1	1	1	1	1	1	1
1	1	1	1	1	1	1	1	1
1	1	1	1	1	1	1	1	1
1	1	1	1	1	1	1	1	1
1	1	1	1	1	1	1	1	1
1	1	1	1	1	1	1	1	1
1	1	1	1	1	1	1	1	1
1	1	1	1	1	1	1	1	1
1	1	1	1	1	1	1	1	1
1	1	1	1	1	1	1	1	1
1	1	1	1	1	1	1	1	1
1	1	1	1	1	1	1	1	1
1	1	1	1	1	1	1	1	1
1	1	1	1	1	1	1	1	1
1	1	1	1	1	1	1	1	1
1	1	1	1	1	1	1	1	1
1	1	1	1	1	1	1	1	1
1	1	1	1	1	1	1	1	1
1	1	1	1	1	1	1	1	1
1	1	1	1	1	1	1	1	1
1	1	1	1	1	1	1	1	1
1	1	1	1	1	1	1	1	1
1	1	1	1	1	1	1	1	1
1	1	1	1	1	1	1	1	1
1	1	1	1	1	1	1	1	1
1	1	1	1	1	1	1	1	1
1	1	1	1	1	1	1	1	1
1	1	1	1	1	1	1	1	1
1	1	1	1	1	1	1	1	1
1	1	1	1	1	1	1	1	1
1	1	1	1	1	1	1	1	1
1	1	1	1	1	1	1	1	1
1	1	1	1	1	1	1	1	1
1	1	1	1	1	1	1	1	1
1	1	1	1	1	1	1	1	1
1	1	1	1	1	1	1	1	1
1	1	1	1	1	1	1	1	1
1	1	1	1	1	1	1	1	1
1	1	1	1	1	1	1	1	1
1	1	1	1	1	1	1	1	1

1	1	1	1	1	1	1	1	1
1	1	1	1	1	1	1	1	1
1	1	1	1	1	1	1	1	1
1	1	1	1	1	1	1	1	1
1	1	1	1	1	1	1	1	1
1	1	1	1	1	1	1	1	1
1	1	1	1	1	1	1	1	1
1	1	1	1	1	1	1	1	1
1	1	1	1	1	1	1	1	1
1	1	1	1	1	1	1	1	1
1	1	1	1	1	1	1	1	1
1	1	1	1	1	1	1	1	1
1	1	1	1	1	1	1	1	1
1	1	1	1	1	1	1	1	1
1	1	1	1	1	1	1	1	1
1	1	1	1	1	1	1	1	1
1	1	1	1	1	1	1	1	1
1	1	1	1	1	1	1	1	1
1	1	1	1	1	1	1	1	1
1	1	1	1	1	1	1	1	1
1	1	1	1	1	1	1	1	1
1	1	1	1	1	1	1	1	1
1	1	1	1	1	1	1	1	1
1	1	1	1	1	1	1	1	1
1	1	1	1	1	1	1	1	1
1	1	1	1	1	1	1	1	1
1	1	1	1	1	1	1	1	1
1	1	1	1	1	1	1	1	1
1	1	1	1	1	1	1	1	1
1	1	1	1	1	1	1	1	1
1	1	1	1	1	1	1	1	1
1	1	1	1	1	1	1	1	1
1	1	1	1	1	1	1	1	1
1	1	1	1	1	1	1	1	1
1	1	1	1	1	1	1	1	1
1	1	1	1	1	1	1	1	1
1	1	1	1	1	1	1	1	1
1	1	1	1	1	1	1	1	1
1	1	1	1	1	1	1	1	1
1	1	1	1	1	1	1	1	1
1	1	1	1	1	1	1	1	1
1	1	1	1	1	1	1	1	1
1	1	1	1	1	1	1	1	1
1	1	1	1	1	1	1	1	1
1	1	1	1	1	1	1	1	1
1	1	1	1	1	1	1	1	1
1	1	1	1	1	1	1	1	1
1	1	1	1	1	1	1	1	1
1	1	1	1	1	1	1	1	1
1	1	1	1	1	1	1	1	1

1	1	1	1	1	1	1	1	1
1	1	1	1	1	1	1	1	1
1	1	1	1	1	1	1	1	1
1	1	1	1	1	1	1	1	1
1	1	1	1	1	1	1	1	1
1	1	1	1	1	1	1	1	1
1	1	1	1	1	1	1	1	1
1	1	1	1	1	1	1	1	1
1	1	1	1	1	1	1	1	1
1	1	1	1	1	1	1	1	1
1	1	1	1	1	1	1	1	1
1	1	1	1	1	1	1	1	1
1	1	1	1	1	1	1	1	1
1	1	1	1	1	1	1	1	1
1	1	1	1	1	1	1	1	1
1	1	1	1	1	1	1	1	1
1	1	1	1	1	1	1	1	1
1	1	1	1	1	1	1	1	1
1	1	1	1	1	1	1	1	1
1	1	1	1	1	1	1	1	1
1	1	1	1	1	1	1	1	1
1	1	1	1	1	1	1	1	1
1	1	1	1	1	1	1	1	1
1	1	1	1	1	1	1	1	1
1	1	1	1	1	1	1	1	1
1	1	1	1	1	1	1	1	1
1	1	1	1	1	1	1	1	1
1	1	1	1	1	1	1	1	1
1	1	1	1	1	1	1	1	1
1	1	1	1	1	1	1	1	1
1	1	1	1	1	1	1	1	1
1	1	1	1	1	1	1	1	1
1	1	1	1	1	1	1	1	1
1	1	1	1	1	1	1	1	1
1	1	1	1	1	1	1	1	1
1	1	1	1	1	1	1	1	1
1	1	1	1	1	1	1	1	1
1	1	1	1	1	1	1	1	1
1	1	1	1	1	1	1	1	1
1	1	1	1	1	1	1	1	1
1	1	1	1	1	1	1	1	1
1	1	1	1	1	1	1	1	1
1	1	1	1	1	1	1	1	1
1	1	1	1	1	1	1	1	1
1	1	1	1	1	1	1	1	1
1	1	1	1	1	1	1	1	1
1	1	1	1	1	1	1	1	1
1	1	1	1	1	1	1	1	1
1	1	1	1	1	1	1	1	1
1	1	1	1	1	1	1	1	1

1	1	1	1	1	1	1	1	1
1	1	1	1	1	1	1	1	1
1	1	1	1	1	1	1	1	1
1	1	1	1	1	1	1	1	1
1	1	1	1	1	1	1	1	1
1	1	1	1	1	1	1	1	1
1	1	1	1	1	1	1	1	1
1	1	1	1	1	1	1	1	1
1	1	1	1	1	1	1	1	1
1	1	1	1	1	1	1	1	1
1	1	1	1	1	1	1	1	1
1	1	1	1	1	1	1	1	1
1	1	1	1	1	1	1	1	1
1	1	1	1	1	1	1	1	1
1	1	1	1	1	1	1	1	1
1	1	1	1	1	1	1	1	1
1	1	1	1	1	1	1	1	1
1	1	1	1	1	1	1	1	1
1	1	1	1	1	1	1	1	1
1	1	1	1	1	1	1	1	1
1	1	1	1	1	1	1	1	1
1	1	1	1	1	1	1	1	1
1	1	1	1	1	1	1	1	1
1	1	1	1	1	1	1	1	1
1	1	1	1	1	1	1	1	1
1	1	1	1	1	1	1	1	1
1	1	1	1	1	1	1	1	1
1	1	1	1	1	1	1	1	1
1	1	1	1	1	1	1	1	1
1	1	1	1	1	1	1	1	1
1	1	1	1	1	1	1	1	1
1	1	1	1	1	1	1	1	1
1	1	1	1	1	1	1	1	1
1	1	1	1	1	1	1	1	1
1	1	1	1	1	1	1	1	1
1	1	1	1	1	1	1	1	1
1	1	1	1	1	1	1	1	1
1	1	1	1	1	1	1	1	1
1	1	1	1	1	1	1	1	1
1	1	1	1	1	1	1	1	1
1	1	1	1	1	1	1	1	1
1	1	1	1	1	1	1	1	1
1	1	1	1	1	1	1	1	1
1	1	1	1	1	1	1	1	1
1	1	1	1	1	1	1	1	1
1	1	1	1	1	1	1	1	1
1	1	1	1	1	1	1	1	1
1	1	1	1	1	1	1	1	1
1	1	1	1	1	1	1	1	1
1	1	1	1	1	1	1	1	1

1	1	1	1	1	1	1	1	1
1	1	1	1	1	1	1	1	1
1	1	1	1	1	1	1	1	1
1	1	1	1	1	1	1	1	1
1	1	1	1	1	1	1	1	1
1	1	1	1	1	1	1	1	1
1	1	1	1	1	1	1	1	1
1	1	1	1	1	1	1	1	1
1	1	1	1	1	1	1	1	1
1	1	1	1	1	1	1	1	1
1	1	1	1	1	1	1	1	1
1	1	1	1	1	1	1	1	1
1	1	1	1	1	1	1	1	1
1	1	1	1	1	1	1	1	1
1	1	1	1	1	1	1	1	1
1	1	1	1	1	1	1	1	1
1	1	1	1	1	1	1	1	1
1	1	1	1	1	1	1	1	1
1	1	1	1	1	1	1	1	1
1	1	1	1	1	1	1	1	1
1	1	1	1	1	1	1	1	1
1	1	1	1	1	1	1	1	1
1	1	1	1	1	1	1	1	1
1	1	1	1	1	1	1	1	1
1	1	1	1	1	1	1	1	1
1	1	1	1	1	1	1	1	1
1	1	1	1	1	1	1	1	1
1	1	1	1	1	1	1	1	1
1	1	1	1	1	1	1	1	1
1	1	1	1	1	1	1	1	1
1	1	1	1	1	1	1	1	1
1	1	1	1	1	1	1	1	1
1	1	1	1	1	1	1	1	1
1	1	1	1	1	1	1	1	1
1	1	1	1	1	1	1	1	1
1	1	1	1	1	1	1	1	1
1	1	1	1	1	1	1	1	1
1	1	1	1	1	1	1	1	1
1	1	1	1	1	1	1	1	1
1	1	1	1	1	1	1	1	1
1	1	1	1	1	1	1	1	1
1	1	1	1	1	1	1	1	1
1	1	1	1	1	1	1	1	1
1	1	1	1	1	1	1	1	1
1	1	1	1	1	1	1	1	1
1	1	1	1	1	1	1	1	1
1	1	1	1	1	1	1	1	1
1	1	1	1	1	1	1	1	1
1	1	1	1	1	1	1	1	1
1	1	1	1	1	1	1	1	1

1	1	1	1	1	1	1	1	1
1	1	1	1	1	1	1	1	1
1	1	1	1	1	1	1	1	1
1	1	1	1	1	1	1	1	1
1	1	1	1	1	1	1	1	1
1	1	1	1	1	1	1	1	1
1	1	1	1	1	1	1	1	1
1	1	1	1	1	1	1	1	1
1	1	1	1	1	1	1	1	1
1	1	1	1	1	1	1	1	1
1	1	1	1	1	1	1	1	1
1	1	1	1	1	1	1	1	1
1	1	1	1	1	1	1	1	1
1	1	1	1	1	1	1	1	1
1	1	1	1	1	1	1	1	1
1	1	1	1	1	1	1	1	1
1	1	1	1	1	1	1	1	1
1	1	1	1	1	1	1	1	1
1	1	1	1	1	1	1	1	1
1	1	1	1	1	1	1	1	1
1	1	1	1	1	1	1	1	1
1	1	1	1	1	1	1	1	1
1	1	1	1	1	1	1	1	1
1	1	1	1	1	1	1	1	1
1	1	1	1	1	1	1	1	1
1	1	1	1	1	1	1	1	1
1	1	1	1	1	1	1	1	1
1	1	1	1	1	1	1	1	1
1	1	1	1	1	1	1	1	1
1	1	1	1	1	1	1	1	1
1	1	1	1	1	1	1	1	1
1	1	1	1	1	1	1	1	1
1	1	1	1	1	1	1	1	1
1	1	1	1	1	1	1	1	1
1	1	1	1	1	1	1	1	1
1	1	1	1	1	1	1	1	1
1	1	1	1	1	1	1	1	1
1	1	1	1	1	1	1	1	1
1	1	1	1	1	1	1	1	1
1	1	1	1	1	1	1	1	1
1	1	1	1	1	1	1	1	1
1	1	1	1	1	1	1	1	1
1	1	1	1	1	1	1	1	1
1	1	1	1	1	1	1	1	1
1	1	1	1	1	1	1	1	1
1	1	1	1	1	1	1	1	1
1	1	1	1	1	1	1	1	1
1	1	1	1	1	1	1	1	1
1	1	1	1	1	1	1	1	1
1	1	1	1	1	1	1	1	1

1	1	1	1	1	1	1	1	1
1	1	1	1	1	1	1	1	1
1	1	1	1	1	1	1	1	1
1	1	1	1	1	1	1	1	1
1	1	1	1	1	1	1	1	1
1	1	1	1	1	1	1	1	1
1	1	1	1	1	1	1	1	1
1	1	1	1	1	1	1	1	1
1	1	1	1	1	1	1	1	1
1	1	1	1	1	1	1	1	1
1	1	1	1	1	1	1	1	1
1	1	1	1	1	1	1	1	1
1	1	1	1	1	1	1	1	1
1	1	1	1	1	1	1	1	1
1	1	1	1	1	1	1	1	1
1	1	1	1	1	1	1	1	1
1	1	1	1	1	1	1	1	1
1	1	1	1	1	1	1	1	1
1	1	1	1	1	1	1	1	1
1	1	1	1	1	1	1	1	1
1	1	1	1	1	1	1	1	1
1	1	1	1	1	1	1	1	1
1	1	1	1	1	1	1	1	1
1	1	1	1	1	1	1	1	1
1	1	1	1	1	1	1	1	1
1	1	1	1	1	1	1	1	1
1	1	1	1	1	1	1	1	1
1	1	1	1	1	1	1	1	1
1	1	1	1	1	1	1	1	1
1	1	1	1	1	1	1	1	1
1	1	1	1	1	1	1	1	1
1	1	1	1	1	1	1	1	1
1	1	1	1	1	1	1	1	1
1	1	1	1	1	1	1	1	1
1	1	1	1	1	1	1	1	1
1	1	1	1	1	1	1	1	1
1	1	1	1	1	1	1	1	1
1	1	1	1	1	1	1	1	1
1	1	1	1	1	1	1	1	1
1	1	1	1	1	1	1	1	1
1	1	1	1	1	1	1	1	1
1	1	1	1	1	1	1	1	1
1	1	1	1	1	1	1	1	1
1	1	1	1	1	1	1	1	1
1	1	1	1	1	1	1	1	1
1	1	1	1	1	1	1	1	1
1	1	1	1	1	1	1	1	1
1	1	1	1	1	1	1	1	1
1	1	1	1	1	1	1	1	1
1	1	1	1	1	1	1	1	1

1	1	1	1	1	1	1	1	1
1	1	1	1	1	1	1	1	1
1	1	1	1	1	1	1	1	1
1	1	1	1	1	1	1	1	1
1	1	1	1	1	1	1	1	1
1	1	1	1	1	1	1	1	1
1	1	1	1	1	1	1	1	1
1	1	1	1	1	1	1	1	1
1	1	1	1	1	1	1	1	1
1	1	1	1	1	1	1	1	1
1	1	1	1	1	1	1	1	1
1	1	1	1	1	1	1	1	1
1	1	1	1	1	1	1	1	1
1	1	1	1	1	1	1	1	1
1	1	1	1	1	1	1	1	1
1	1	1	1	1	1	1	1	1
1	1	1	1	1	1	1	1	1
1	1	1	1	1	1	1	1	1
1	1	1	1	1	1	1	1	1
1	1	1	1	1	1	1	1	1
1	1	1	1	1	1	1	1	1
1	1	1	1	1	1	1	1	1
1	1	1	1	1	1	1	1	1
1	1	1	1	1	1	1	1	1
1	1	1	1	1	1	1	1	1
1	1	1	1	1	1	1	1	1
1	1	1	1	1	1	1	1	1
1	1	1	1	1	1	1	1	1
1	1	1	1	1	1	1	1	1
1	1	1	1	1	1	1	1	1
1	1	1	1	1	1	1	1	1
1	1	1	1	1	1	1	1	1
1	1	1	1	1	1	1	1	1
1	1	1	1	1	1	1	1	1
1	1	1	1	1	1	1	1	1
1	1	1	1	1	1	1	1	1
1	1	1	1	1	1	1	1	1
1	1	1	1	1	1	1	1	1
1	1	1	1	1	1	1	1	1
1	1	1	1	1	1	1	1	1
1	1	1	1	1	1	1	1	1
1	1	1	1	1	1	1	1	1
1	1	1	1	1	1	1	1	1
1	1	1	1	1	1	1	1	1
1	1	1	1	1	1	1	1	1
1	1	1	1	1	1	1	1	1
1	1	1	1	1	1	1	1	1
1	1	1	1	1	1	1	1	1
1	1	1	1	1	1	1	1	1
1	1	1	1	1	1	1	1	1

1	1	1	1	1	1	1	1	1
1	1	1	1	1	1	1	1	1
1	1	1	1	1	1	1	1	1
1	1	1	1	1	1	1	1	1
1	1	1	1	1	1	1	1	1
1	1	1	1	1	1	1	1	1
1	1	1	1	1	1	1	1	1
1	1	1	1	1	1	1	1	1
1	1	1	1	1	1	1	1	1
1	1	1	1	1	1	1	1	1
1	1	1	1	1	1	1	1	1
1	1	1	1	1	1	1	1	1
1	1	1	1	1	1	1	1	1
1	1	1	1	1	1	1	1	1
1	1	1	1	1	1	1	1	1
1	1	1	1	1	1	1	1	1
1	1	1	1	1	1	1	1	1
1	1	1	1	1	1	1	1	1
1	1	1	1	1	1	1	1	1
1	1	1	1	1	1	1	1	1
1	1	1	1	1	1	1	1	1
1	1	1	1	1	1	1	1	1
1	1	1	1	1	1	1	1	1
1	1	1	1	1	1	1	1	1
1	1	1	1	1	1	1	1	1
1	1	1	1	1	1	1	1	1
1	1	1	1	1	1	1	1	1
1	1	1	1	1	1	1	1	1
1	1	1	1	1	1	1	1	1
1	1	1	1	1	1	1	1	1
1	1	1	1	1	1	1	1	1
1	1	1	1	1	1	1	1	1
1	1	1	1	1	1	1	1	1
1	1	1	1	1	1	1	1	1
1	1	1	1	1	1	1	1	1
1	1	1	1	1	1	1	1	1
1	1	1	1	1	1	1	1	1
1	1	1	1	1	1	1	1	1
1	1	1	1	1	1	1	1	1
1	1	1	1	1	1	1	1	1
1	1	1	1	1	1	1	1	1
1	1	1	1	1	1	1	1	1
1	1	1	1	1	1	1	1	1
1	1	1	1	1	1	1	1	1
1	1	1	1	1	1	1	1	1
1	1	1	1	1	1	1	1	1
1	1	1	1	1	1	1	1	1
1	1	1	1	1	1	1	1	1
1	1	1	1	1	1	1	1	1
1	1	1	1	1	1	1	1	1

1	1	1	1	1	1	1	1	1
1	1	1	1	1	1	1	1	1
1	1	1	1	1	1	1	1	1
1	1	1	1	1	1	1	1	1
1	1	1	1	1	1	1	1	1
1	1	1	1	1	1	1	1	1
1	1	1	1	1	1	1	1	1
1	1	1	1	1	1	1	1	1
1	1	1	1	1	1	1	1	1
1	1	1	1	1	1	1	1	1
1	1	1	1	1	1	1	1	1
1	1	1	1	1	1	1	1	1
1	1	1	1	1	1	1	1	1
1	1	1	1	1	1	1	1	1
1	1	1	1	1	1	1	1	1
1	1	1	1	1	1	1	1	1
1	1	1	1	1	1	1	1	1
1	1	1	1	1	1	1	1	1
1	1	1	1	1	1	1	1	1
1	1	1	1	1	1	1	1	1
1	1	1	1	1	1	1	1	1
1	1	1	1	1	1	1	1	1
1	1	1	1	1	1	1	1	1
1	1	1	1	1	1	1	1	1
1	1	1	1	1	1	1	1	1
1	1	1	1	1	1	1	1	1
1	1	1	1	1	1	1	1	1
1	1	1	1	1	1	1	1	1
1	1	1	1	1	1	1	1	1
1	1	1	1	1	1	1	1	1
1	1	1	1	1	1	1	1	1
1	1	1	1	1	1	1	1	1
1	1	1	1	1	1	1	1	1
1	1	1	1	1	1	1	1	1
1	1	1	1	1	1	1	1	1
1	1	1	1	1	1	1	1	1
1	1	1	1	1	1	1	1	1
1	1	1	1	1	1	1	1	1
1	1	1	1	1	1	1	1	1
1	1	1	1	1	1	1	1	1
1	1	1	1	1	1	1	1	1
1	1	1	1	1	1	1	1	1
1	1	1	1	1	1	1	1	1
1	1	1	1	1	1	1	1	1
1	1	1	1	1	1	1	1	1
1	1	1	1	1	1	1	1	1
1	1	1	1	1	1	1	1	1
1	1	1	1	1	1	1	1	1
1	1	1	1	1	1	1	1	1
1	1	1	1	1	1	1	1	1

1	1	1	1	1	1	1	1	1
1	1	1	1	1	1	1	1	1
1	1	1	1	1	1	1	1	1
1	1	1	1	1	1	1	1	1
1	1	1	1	1	1	1	1	1
1	1	1	1	1	1	1	1	1
1	1	1	1	1	1	1	1	1
1	1	1	1	1	1	1	1	1
1	1	1	1	1	1	1	1	1
1	1	1	1	1	1	1	1	1
1	1	1	1	1	1	1	1	1
1	1	1	1	1	1	1	1	1
1	1	1	1	1	1	1	1	1
1	1	1	1	1	1	1	1	1
1	1	1	1	1	1	1	1	1
1	1	1	1	1	1	1	1	1
1	1	1	1	1	1	1	1	1
1	1	1	1	1	1	1	1	1
1	1	1	1	1	1	1	1	1
1	1	1	1	1	1	1	1	1
1	1	1	1	1	1	1	1	1
1	1	1	1	1	1	1	1	1
1	1	1	1	1	1	1	1	1
1	1	1	1	1	1	1	1	1
1	1	1	1	1	1	1	1	1
1	1	1	1	1	1	1	1	1
1	1	1	1	1	1	1	1	1
1	1	1	1	1	1	1	1	1
1	1	1	1	1	1	1	1	1
1	1	1	1	1	1	1	1	1
1	1	1	1	1	1	1	1	1
1	1	1	1	1	1	1	1	1
1	1	1	1	1	1	1	1	1
1	1	1	1	1	1	1	1	1
1	1	1	1	1	1	1	1	1
1	1	1	1	1	1	1	1	1
1	1	1	1	1	1	1	1	1
1	1	1	1	1	1	1	1	1
1	1	1	1	1	1	1	1	1
1	1	1	1	1	1	1	1	1
1	1	1	1	1	1	1	1	1
1	1	1	1	1	1	1	1	1
1	1	1	1	1	1	1	1	1
1	1	1	1	1	1	1	1	1
1	1	1	1	1	1	1	1	1
1	1	1	1	1	1	1	1	1
1	1	1	1	1	1	1	1	1
1	1	1	1	1	1	1	1	1
1	1	1	1	1	1	1	1	1
1	1	1	1	1	1	1	1	1

1	1	1	1	1	1	1	1	1
1	1	1	1	1	1	1	1	1
1	1	1	1	1	1	1	1	1
1	1	1	1	1	1	1	1	1
1	1	1	1	1	1	1	1	1
1	1	1	1	1	1	1	1	1
1	1	1	1	1	1	1	1	1
1	1	1	1	1	1	1	1	1
1	1	1	1	1	1	1	1	1
1	1	1	1	1	1	1	1	1
1	1	1	1	1	1	1	1	1
1	1	1	1	1	1	1	1	1
1	1	1	1	1	1	1	1	1
1	1	1	1	1	1	1	1	1
1	1	1	1	1	1	1	1	1
1	1	1	1	1	1	1	1	1
1	1	1	1	1	1	1	1	1
1	1	1	1	1	1	1	1	1
1	1	1	1	1	1	1	1	1
1	1	1	1	1	1	1	1	1
1	1	1	1	1	1	1	1	1
1	1	1	1	1	1	1	1	1
1	1	1	1	1	1	1	1	1
1	1	1	1	1	1	1	1	1
1	1	1	1	1	1	1	1	1
1	1	1	1	1	1	1	1	1
1	1	1	1	1	1	1	1	1
1	1	1	1	1	1	1	1	1
1	1	1	1	1	1	1	1	1
1	1	1	1	1	1	1	1	1
1	1	1	1	1	1	1	1	1
1	1	1	1	1	1	1	1	1
1	1	1	1	1	1	1	1	1
1	1	1	1	1	1	1	1	1
1	1	1	1	1	1	1	1	1
1	1	1	1	1	1	1	1	1
1	1	1	1	1	1	1	1	1
1	1	1	1	1	1	1	1	1
1	1	1	1	1	1	1	1	1
1	1	1	1	1	1	1	1	1
1	1	1	1	1	1	1	1	1
1	1	1	1	1	1	1	1	1
1	1	1	1	1	1	1	1	1
1	1	1	1	1	1	1	1	1
1	1	1	1	1	1	1	1	1
1	1	1	1	1	1	1	1	1
1	1	1	1	1	1	1	1	1
1	1	1	1	1	1	1	1	1
1	1	0	1	1	1	1	1	1
1	1	1	1	1	1	1	1	1

1	1	1	1	1	1	1	1	1
1	1	1	1	1	1	1	1	1
1	1	1	1	1	1	1	1	1
0	1	1	1	1	1	1	1	1
0	1	1	1	1	1	1	1	1
1	1	1	1	1	1	1	1	0
1	1	1	1	1	1	1	1	1
1	1	1	1	1	1	1	1	1
1	1	1	1	1	1	1	1	1
0	1	1	1	1	1	1	1	1
1	1	1	1	1	1	1	1	1
1	1	1	1	1	1	1	1	1
1	1	1	1	1	1	1	1	1
1	1	1	1	1	1	1	1	0
0	1	1	1	1	1	1	1	1
1	1	1	1	1	1	1	1	1
1	1	1	1	1	1	1	1	1
1	1	1	1	1	1	1	1	0
1	1	1	1	1	1	1	1	1
1	1	1	1	1	1	1	1	1
0	1	1	1	1	1	1	1	1
1	1	1	1	1	1	1	1	1
1	1	1	1	1	1	1	1	1
1	1	1	1	0	1	1	1	1
1	0	1	1	1	1	1	1	1
1	1	1	1	1	1	1	1	1
1	1	1	1	1	1	1	1	1
1	1	1	1	1	0	1	1	1
1	1	1	1	1	1	1	1	1
1	0	1	1	1	1	1	1	1
1	1	1	1	1	1	1	1	1
1	1	1	1	1	1	1	1	1
1	1	1	1	1	1	1	1	1
1	1	1	1	1	1	1	1	1
1	1	1	1	1	1	1	1	1
1	0	1	1	1	1	1	1	1
1	0	1	1	1	1	1	1	1
1	1	0	1	1	1	1	1	1
1	1	1	1	1	1	1	1	1
1	1	1	1	1	1	1	1	1
1	1	1	1	1	1	1	1	1
1	1	1	1	1	1	1	1	1
1	1	1	1	1	1	1	1	1
1	1	1	1	1	1	1	1	1
1	1	1	0	1	1	1	1	1
1	1	1	1	0	1	1	1	1
1	1	1	1	1	1	1	1	1
1	1	0	1	1	1	1	1	1
0	1	1	1	1	1	1	1	1
1	1	1	1	1	1	1	1	1

1	1	1	1	1	1	1	1	1
1	1	1	1	1	1	1	1	1
1	1	1	1	1	1	1	1	0
1	1	1	1	1	1	1	1	1
1	1	1	1	1	1	1	0	1
1	1	1	1	1	1	0	1	1
1	1	1	1	1	1	1	1	1
1	1	1	1	1	0	1	1	1
1	1	1	1	1	1	0	1	1
1	1	1	1	1	1	1	1	0
1	1	1	1	1	1	1	1	1
0	1	1	1	1	1	1	1	0
1	1	1	1	1	1	0	1	1
1	1	1	0	1	1	1	1	1
1	1	1	1	1	1	1	0	1
0	1	1	1	1	1	1	1	1
1	1	1	1	1	1	1	1	1
1	1	1	1	1	0	1	1	1
1	1	1	1	1	1	1	0	1
1	1	1	1	1	1	1	0	1
1	1	1	0	1	1	1	1	1
1	1	1	1	1	1	1	1	1
1	1	1	1	1	1	1	1	1
1	1	1	0	1	1	1	1	1
1	1	1	1	1	1	1	1	1
1	1	1	1	1	1	1	1	0
1	1	1	1	1	1	1	1	1
1	1	1	1	1	1	1	0	1
1	1	1	1	1	1	1	1	0
1	1	1	1	1	1	1	1	1
1	1	1	1	1	1	1	1	1
1	1	1	1	1	1	1	1	1
1	1	1	1	1	1	1	1	1
1	1	1	1	1	1	1	1	1
1	1	1	0	1	1	1	1	1
1	1	1	0	1	1	1	1	1
1	1	1	1	1	0	1	1	1
1	1	1	1	1	1	0	1	1
1	1	1	1	1	1	1	1	1
1	0	1	1	1	1	1	1	1
1	1	1	1	1	1	1	1	1
0	1	1	1	0	1	1	1	1
1	1	1	1	1	1	1	0	1
1	1	1	1	1	1	1	1	1
1	1	1	1	1	1	1	1	1
1	1	1	1	1	0	1	1	1
1	1	1	0	1	1	1	1	1
1	1	1	0	1	1	1	1	1
1	1	1	0	0	1	1	1	1
1	1	1	1	1	0	1	1	1

1	0	0	1	1	1	1	1	1
1	0	1	1	1	1	1	1	0
1	1	1	0	1	1	1	1	1
1	1	1	1	1	1	1	1	1
1	1	1	1	1	1	0	1	1
1	0	1	1	1	1	1	1	0
1	1	1	1	1	0	1	1	1
1	1	1	1	1	1	1	1	1
1	0	1	0	1	1	1	1	1
1	1	1	1	1	1	1	1	1
1	1	1	0	1	1	1	1	1
1	1	1	1	1	1	1	1	1
1	0	0	1	1	1	1	1	1
1	1	1	0	1	1	1	0	1
1	1	1	1	1	1	1	1	1
1	1	1	1	1	1	1	1	1
1	1	1	1	1	1	1	1	1
1	1	1	1	1	0	1	1	1
1	0	1	1	1	1	1	1	0
1	1	1	0	1	1	1	1	1
1	0	1	1	1	1	1	1	0
1	1	1	0	1	1	1	1	1
0	1	0	1	1	1	1	0	1
1	0	1	1	1	1	1	1	0
1	1	1	1	1	0	1	1	1
1	1	1	1	1	1	0	1	1
1	1	1	1	1	0	1	1	1
1	1	1	0	1	1	1	1	0
1	1	1	1	1	0	1	0	1
1	1	1	0	1	1	1	1	1
0	1	1	1	1	1	1	1	1
1	0	1	1	1	1	1	1	0
1	0	1	1	1	1	1	1	1
1	1	1	0	1	1	0	1	1
1	1	1	1	1	0	1	1	1
1	0	1	1	1	1	1	1	1
1	1	1	1	1	1	0	1	1
1	1	1	0	1	1	1	1	0
0	1	1	1	0	1	1	1	1
1	1	1	0	1	0	1	1	1
0	1	0	1	0	1	1	0	1
1	1	1	1	1	0	1	0	1
1	1	1	1	1	0	1	0	1
1	1	1	1	1	0	1	1	1
1	0	1	1	1	1	1	1	0
1	1	1	0	1	1	1	1	1
1	0	1	1	1	1	1	1	0
1	1	1	0	1	0	1	1	1
1	1	1	1	1	0	1	0	1
0	1	1	1	0	1	1	1	1

0	1	1	1	0	1	1	1	1
0	1	1	1	0	1	1	1	1
0	1	1	1	0	1	1	1	1
1	1	1	1	1	0	1	0	1
1	1	1	1	1	0	1	0	1
1	1	1	1	1	0	1	0	1
0	1	1	1	0	1	1	1	1
0	1	1	1	0	1	1	1	1
1	1	0	0	1	1	0	1	0
1	1	1	1	1	0	1	0	1
0	1	1	1	0	1	1	1	1
1	0	1	1	1	1	1	1	1
1	1	1	1	1	0	1	0	1
1	1	0	0	1	1	1	0	1
1	1	1	1	1	0	1	0	1
1	1	1	1	1	0	1	0	1
1	1	1	1	1	0	1	0	1
1	0	1	1	1	1	0	1	0
1	0	1	1	1	0	0	1	1
1	1	1	1	1	0	1	0	1
1	1	1	1	1	1	0	1	0
1	1	1	0	1	0	1	1	0
1	1	0	0	1	1	1	0	1
1	1	1	0	1	0	1	0	1
1	1	1	0	1	1	0	1	1
1	0	0	0	1	1	1	0	1
1	1	1	0	1	1	1	1	1
1	1	0	1	1	0	1	0	1
1	1	1	0	1	0	1	1	1
1	1	0	1	1	0	1	0	1
1	1	1	0	1	1	0	1	1
1	1	1	0	1	1	0	1	1
1	1	1	0	1	1	0	1	1
1	1	0	1	1	0	1	0	1
1	1	0	1	1	0	1	0	1
1	1	1	0	1	1	0	1	1
1	1	1	0	1	0	1	1	1
0	1	1	1	1	0	1	1	1
1	1	1	1	1	0	1	0	1
1	1	1	0	1	0	1	1	1
1	0	1	1	1	1	0	1	0
1	1	1	0	1	1	0	1	1
0	1	1	0	1	1	0	1	1
1	1	1	0	1	0	0	1	1
1	1	1	0	1	0	1	1	1
1	1	1	1	1	0	0	1	0
1	0	1	1	1	0	0	1	1
1	1	1	0	1	0	1	0	1
0	1	0	1	0	1	1	0	1
1	0	1	1	1	1	0	1	1

1	0	1	0	1	1	1	1	0
0	1	0	1	0	1	1	0	1
1	0	1	1	1	1	0	1	0
0	1	0	1	0	1	1	0	1
0	1	0	1	0	1	1	0	1
0	0	1	0	1	1	1	1	1
1	0	1	0	1	1	0	1	1
1	1	1	0	1	0	1	1	1
1	0	1	0	1	0	0	1	1
0	1	0	1	0	0	1	0	1
1	1	0	0	1	1	1	0	1
1	1	0	0	1	1	1	0	1
1	1	1	0	1	0	1	1	1
1	0	1	0	1	1	1	1	0
1	0	1	0	1	1	1	1	0
1	0	1	0	1	1	1	1	0
0	1	1	1	0	1	1	0	1
1	0	1	1	1	1	0	1	0
1	1	1	0	1	0	1	1	1
1	1	1	0	1	0	1	1	1
1	0	1	0	1	1	0	0	0
1	1	1	0	1	0	1	1	1
0	1	1	0	1	1	1	0	1
1	1	1	0	1	0	1	1	0
0	1	1	0	0	1	1	1	1
1	0	1	0	1	1	0	1	0
1	0	1	0	1	0	1	1	1
1	0	1	1	1	0	0	1	0
1	1	1	1	1	0	0	1	0
1	1	1	0	0	1	0	0	0
0	0	1	0	1	1	0	1	0
1	0	1	0	1	1	0	1	0
1	0	1	0	0	1	1	1	0
1	1	0	1	0	0	1	0	1
1	0	1	0	1	1	0	1	0
1	0	0	0	1	1	0	0	0
1	1	1	1	1	0	0	1	0
1	0	1	0	1	1	0	1	0
1	0	1	0	1	0	1	1	0
1	0	1	0	1	1	0	1	0
1	0	1	0	1	1	0	1	0
1	0	1	0	1	1	0	1	0
1	0	1	0	1	0	1	1	0
0	0	0	1	0	1	0	0	1
1	1	1	0	1	0	0	1	0
0	1	0	1	0	0	1	0	0
0	1	0	1	0	0	1	0	0
0	1	0	1	0	0	1	0	0
0	1	0	1	0	0	1	0	0
1	0	1	0	1	1	0	1	0

1	0	1	1	1	0	0	1	0
1	0	0	0	1	0	1	1	0
0	1	1	0	0	1	0	1	1
0	1	1	0	0	1	0	1	1
0	1	1	0	0	1	0	1	1
0	1	0	1	0	0	1	0	0
0	1	0	1	0	0	1	0	0
0	0	1	0	1	1	0	1	0
1	0	0	0	0	1	0	0	0
1	0	1	0	1	0	0	1	0
0	1	1	0	0	1	0	1	1
0	0	0	0	0	1	0	1	0
1	0	1	1	1	0	0	1	0
0	1	1	0	1	1	0	0	1
0	0	0	1	0	1	0	0	0
1	0	1	0	1	0	0	1	0
0	0	1	1	0	0	0	1	0
1	0	1	1	1	0	0	0	0
0	1	1	1	0	1	0	1	0
0	1	1	1	0	1	0	1	0
0	0	0	0	0	1	1	1	1
1	0	1	1	1	0	0	1	0
0	1	1	1	0	1	0	1	0
0	1	1	1	0	1	0	1	0
0	1	1	1	0	1	0	1	0
0	1	0	1	0	0	1	0	0
0	0	0	1	0	0	1	0	1
0	0	0	1	0	0	1	0	1
0	0	0	1	0	0	1	0	1
1	0	1	1	1	1	1	0	0
0	1	0	1	0	0	1	0	0
1	0	1	0	1	0	0	1	0
1	0	0	0	0	0	1	1	0
1	0	0	0	0	0	1	1	0
1	0	1	0	1	0	0	1	0
1	0	1	0	1	0	0	1	0
1	0	1	0	1	0	0	1	0
1	0	1	0	1	0	0	1	0
1	0	0	1	0	0	1	1	1
0	0	0	1	0	1	1	0	0
1	0	1	0	1	0	0	1	0
1	0	1	0	1	0	0	1	0
1	0	1	0	1	0	0	1	0
1	0	1	0	1	0	0	1	0
1	0	1	0	1	0	0	1	0
1	0	1	0	1	0	0	1	0
1	0	1	0	1	0	0	1	0
1	0	1	0	1	0	0	1	0
1	0	1	0	1	0	0	1	0
1	0	1	0	1	0	0	1	0

1	0	1	0	1	0	0	1	0
1	0	1	0	1	0	0	1	0
1	0	1	0	1	0	0	1	0
1	0	1	0	1	0	0	1	0
1	0	1	0	1	0	0	1	0
1	0	1	0	1	0	0	1	0
1	0	1	0	1	0	0	1	0
1	0	1	0	1	0	0	1	0
1	0	1	0	1	0	0	1	0
1	0	1	0	1	0	0	1	0
1	0	1	0	1	0	0	1	0
1	0	1	0	1	0	0	1	0
1	0	1	0	1	0	0	1	0
1	0	1	0	1	0	0	1	0
1	0	1	0	1	0	0	1	0
1	0	1	0	1	0	0	1	0
1	0	1	0	1	0	0	1	0
1	0	1	0	1	0	0	1	0
1	0	1	0	1	0	0	1	0
1	0	1	0	1	0	0	1	0
1	0	1	0	1	0	0	1	0
1	0	1	0	1	0	0	1	0
1	0	1	0	1	0	0	1	0
0	0	1	0	0	1	0	1	1
0	0	0	0	0	1	1	0	1
0	0	0	1	0	1	0	0	0
0	1	0	1	0	0	1	0	1
1	0	1	0	1	0	0	1	0
1	0	1	0	1	0	0	1	0
0	0	0	1	0	0	1	0	0
1	0	1	0	1	0	0	1	0
1	0	0	0	1	0	0	1	0
0	0	0	0	0	1	0	1	1
1	0	1	0	1	0	0	1	0
0	0	0	0	0	1	0	0	0
0	1	0	1	0	0	0	0	0
0	0	0	0	0	1	1	0	0
0	1	0	1	0	0	1	0	0
1	0	0	0	1	0	1	0	0
0	0	0	0	0	1	1	0	0
0	1	0	1	0	0	1	0	0
0	1	0	1	0	0	1	0	0
0	1	0	0	0	0	1	0	1
0	0	0	0	0	0	1	0	0
0	0	0	0	0	1	1	1	0
0	0	0	1	0	1	0	0	0
0	0	0	0	0	1	1	0	0
1	1	0	0	0	1	0	0	0
1	0	0	0	0	0	0	1	0
1	0	0	0	0	0	0	1	0

0	0	0	0	0	0	1	0	1
0	0	0	0	0	1	1	0	0
0	0	0	0	0	0	1	0	0
0	0	0	0	0	0	1	0	0
0	0	0	0	0	0	1	0	0
1	0	1	0	1	1	0	0	0
0	0	0	0	0	1	0	1	0
0	1	0	1	0	1	0	0	0
0	1	0	1	0	1	0	0	0
0	1	0	1	0	1	0	0	0
0	1	0	1	0	1	0	0	0
0	1	0	1	0	1	0	0	0
0	1	0	0	0	0	1	0	1
0	0	0	0	0	0	1	0	0
0	1	0	1	0	0	0	0	1
0	0	0	0	0	0	0	0	1
1	0	0	0	0	0	0	0	1
0	1	0	1	0	0	0	0	1
0	1	0	1	0	0	0	0	1
0	1	0	0	0	0	0	0	1
0	1	0	1	0	0	0	0	0
0	1	0	0	0	1	0	0	0
0	1	0	1	0	0	0	0	0
1	0	1	0	0	0	0	0	0
1	0	0	0	1	0	0	0	0
1	0	0	0	1	0	0	0	0
0	0	0	1	0	1	0	0	0
0	0	0	1	0	1	0	0	0
0	0	0	1	0	0	0	0	0
1	0	1	0	1	0	0	0	0
1	0	0	0	0	0	0	0	0
0	1	0	1	0	0	0	0	1
0	1	0	1	0	0	0	0	1
0	1	0	1	0	0	0	0	1
0	1	0	0	0	0	0	0	1
0	1	0	0	0	0	0	0	1
0	0	0	0	0	0	1	0	1
0	0	0	1	0	1	0	0	0
0	0	0	1	0	0	0	0	0
0	0	0	1	0	0	0	0	0
0	0	0	0	0	0	1	0	0
0	0	0	1	0	0	1	0	0
0	1	0	0	0	0	0	0	1
0	1	0	0	0	0	0	0	1
0	0	0	0	0	0	0	1	0
0	1	0	0	0	0	0	0	1
0	1	0	0	0	0	0	0	1
0	0	1	0	0	1	0	1	0
0	0	0	1	0	0	0	0	0
0	0	0	1	0	0	0	0	0

0	1	0	0	0	0	0	0	1
0	1	0	0	0	0	0	0	1
0	1	0	0	0	0	0	0	1
0	1	0	0	0	0	0	0	1
0	1	0	0	0	0	0	0	1
0	1	0	0	0	0	0	0	1
0	1	0	0	0	0	0	0	1
0	1	0	0	0	0	0	0	1
0	1	0	0	0	0	0	0	1
0	1	0	0	0	0	0	0	1
0	1	0	0	0	0	0	0	1
0	1	0	0	0	0	0	0	1
0	1	0	0	0	0	0	0	1
0	1	0	0	0	0	0	0	1
0	1	0	0	0	0	0	0	1
0	0	0	0	0	1	0	0	0
0	1	0	0	0	1	0	0	0
0	0	0	0	0	1	0	0	0
0	0	0	1	0	0	0	0	1
0	0	0	1	0	0	0	0	0
0	0	0	1	0	0	1	0	0
0	0	0	1	0	0	1	0	1
0	0	0	0	0	0	1	0	0
0	0	0	1	0	0	0	0	0
0	0	0	1	0	0	0	1	0
0	0	0	1	0	0	0	1	0
0	0	0	1	0	0	0	1	0
0	0	0	1	0	0	0	1	0
0	0	0	1	0	0	0	0	0
0	0	0	1	0	0	0	0	0
0	0	0	0	0	0	0	0	0
0	1	0	0	0	0	0	0	1
0	0	0	0	0	0	0	1	0
0	0	0	0	0	1	0	0	0
1	0	1	0	0	0	0	0	0
0	0	0	0	0	0	0	0	0
0	0	0	0	0	0	0	0	0
0	0	0	1	0	0	0	0	0
0	0	0	1	0	0	0	0	0
0	0	0	0	0	1	0	0	1
0	1	0	0	0	0	0	0	1
0	0	1	0	0	0	0	0	1
0	1	0	0	0	0	0	0	1
0	0	0	0	0	0	0	0	0
0	0	0	0	0	0	0	1	0
0	1	0	0	0	0	1	0	0
0	0	0	1	0	0	0	0	0
1	0	1	0	0	0	0	1	0
0	1	0	0	0	0	0	0	0
0	0	0	0	0	0	0	0	1

0	1	0	0	0	0	0	0	0
0	1	0	0	0	0	0	0	0
0	1	0	0	0	0	0	0	0
1	0	0	0	0	0	0	1	0
0	0	0	1	0	0	0	0	0
0	0	1	0	0	0	0	1	0
0	0	0	0	0	0	0	0	0
0	0	0	0	0	0	0	0	0
0	0	0	0	0	0	0	0	0
0	0	0	0	0	0	0	0	0
0	0	0	0	0	1	0	0	0
0	1	0	0	0	1	0	0	0
0	0	0	1	0	0	0	0	0
0	0	0	1	0	0	0	0	0
0	0	0	0	0	1	0	0	0
0	0	0	0	0	1	0	0	0
0	0	0	0	0	1	0	0	0
0	0	0	0	0	1	0	0	0
0	0	0	0	0	1	0	0	0
0	0	0	0	0	1	0	0	0
0	0	0	0	0	1	0	0	0
0	0	0	0	0	1	0	0	0
0	0	0	0	0	1	0	0	0
0	0	0	0	0	1	0	0	0
0	0	0	0	0	1	0	0	0
0	0	0	0	0	1	0	0	0
0	0	0	0	0	1	0	0	0
0	0	0	0	0	1	0	0	0
0	0	0	0	0	1	0	0	0
0	0	0	0	0	1	0	0	0
0	0	0	0	0	1	0	0	0
1	0	0	0	0	0	0	0	0
0	1	0	0	0	0	0	0	1
0	1	0	0	0	0	0	0	1
0	1	0	0	0	0	0	0	1
0	1	0	0	0	0	0	0	1
0	1	0	0	0	0	0	0	1
0	1	0	0	0	0	0	0	1
0	1	0	0	0	0	0	0	1
0	1	0	0	0	0	0	0	1
0	1	0	0	0	0	0	0	1
0	1	0	0	0	0	0	0	1
0	1	0	0	0	0	0	0	1
0	1	0	0	0	0	0	0	1
0	1	0	0	0	0	0	0	1
0	1	0	0	0	0	0	0	1
0	1	0	0	0	0	0	0	1
0	1	0	0	0	0	0	0	1
0	1	0	0	0	0	0	0	1
0	1	0	0	0	0	0	0	1

0	1	0	0	0	0	0	0	1
0	1	0	0	0	0	0	0	1
0	1	0	0	0	0	0	0	1
0	1	0	0	0	0	0	0	1
0	1	0	0	0	0	0	0	1
0	1	0	0	0	0	0	0	1
0	1	0	0	0	0	0	0	1
0	1	0	0	0	0	0	0	1
0	1	0	0	0	0	0	0	1
0	1	0	0	0	0	0	0	1
0	1	0	0	0	0	0	0	1
0	0	0	0	0	0	0	1	0
0	0	0	0	0	0	0	0	0
0	0	0	0	0	0	1	1	0
0	0	0	0	0	0	1	0	0
0	0	0	0	0	0	1	0	0
0	0	0	1	0	0	0	0	0
0	0	0	1	0	0	0	1	0
0	0	0	1	0	0	0	1	0
0	0	0	1	0	0	0	1	0
0	0	0	1	0	0	0	1	0
0	0	0	1	0	0	0	1	0
0	1	0	0	0	0	0	0	1
0	0	0	0	0	0	0	0	0
0	1	0	0	0	0	0	0	0
0	1	0	0	0	0	0	0	0
0	1	0	0	0	0	0	0	0
0	1	0	0	0	0	0	0	0
0	1	0	0	0	0	0	0	0
0	0	1	0	0	0	0	1	0
0	0	1	0	0	0	0	1	0
0	0	1	0	0	0	0	1	0
0	0	0	0	0	1	0	0	0
0	0	0	0	0	1	0	0	0
0	0	1	0	0	0	0	1	0
0	0	0	1	0	0	0	0	0
0	0	0	0	1	0	0	0	0
0	0	0	0	0	0	0	1	0
0	0	0	0	0	0	0	0	0
0	0	0	0	0	0	0	0	0
0	0	1	0	0	0	0	0	0
1	0	0	0	0	0	0	1	0
0	0	0	0	0	0	0	0	0
0	0	0	0	0	0	0	0	0
0	0	0	0	0	0	0	0	0
0	0	0	0	0	0	0	0	1
0	0	0	0	0	0	0	0	0
0	0	0	0	0	0	0	0	0
1	0	0	0	0	0	0	0	0
1	0	0	0	0	0	0	0	0

0	0	0	0	0	0	0	0	1
0	0	0	0	0	0	0	0	0
0	0	0	0	0	0	0	0	0
0	0	0	0	0	0	0	0	0
0	0	0	0	0	0	0	0	0
0	0	0	0	0	0	0	0	0
0	0	0	1	0	0	0	0	0
0	0	0	0	0	0	0	0	1
0	0	0	0	0	0	0	0	0
0	0	0	0	0	0	0	0	0
0	0	0	0	0	0	0	0	0
0	0	0	0	0	0	0	0	0
0	0	0	0	0	0	0	0	0
0	0	0	0	0	0	0	0	0
0	0	0	0	0	0	0	0	0
0	0	0	0	0	0	0	0	0
0	1	0	0	0	0	0	0	0
0	0	0	0	0	0	0	0	1
0	0	0	0	0	0	0	0	0
0	0	0	0	0	0	0	0	0
0	0	0	1	0	0	0	0	0
0	1	0	0	0	0	0	0	0
0	0	0	1	0	0	0	0	0
0	0	0	0	0	0	0	0	0
1	0	0	0	0	0	0	0	0
0	1	0	0	0	0	0	0	0
0	0	0	0	0	0	1	0	0
0	0	0	0	0	0	0	0	0
0	0	0	0	0	0	0	0	1
0	0	0	0	0	0	0	0	1
0	0	0	0	0	0	0	0	1
0	0	0	0	0	0	0	0	1
0	0	0	0	0	0	0	0	1
0	0	0	0	0	0	0	0	1
0	0	0	0	0	0	0	0	1
0	0	0	0	0	0	0	0	1
0	0	0	0	0	0	0	0	1
0	0	0	0	0	0	0	0	1
0	0	0	0	0	0	0	0	1
0	0	0	0	0	1	0	0	0
0	0	0	0	0	0	0	0	0
0	0	0	0	0	0	0	0	0
0	0	0	0	0	0	1	0	0
0	0	0	0	0	0	1	0	0
0	0	0	0	0	0	1	0	0
0	0	0	0	0	0	1	0	0
0	0	0	1	0	0	0	0	0
0	0	0	0	0	0	1	0	0
0	0	0	0	0	0	1	0	0
0	0	0	0	0	0	1	0	0

0	0	0	0	0	0	1	0	0
0	0	0	0	0	0	1	0	0
0	0	0	0	0	0	1	0	0
0	0	0	0	0	0	1	0	0
0	0	0	0	0	0	1	0	0
0	0	0	0	0	0	1	0	0
0	0	0	0	0	0	1	0	0
0	0	0	0	0	0	1	0	0
0	0	0	1	0	0	0	0	0
0	0	0	1	0	0	0	0	0
0	0	0	1	0	0	0	0	0
0	0	0	1	0	0	0	0	0
0	0	0	0	0	0	0	1	0
0	0	0	0	0	0	0	1	0
0	0	0	0	0	0	0	1	0
0	0	0	1	0	0	0	0	0
0	0	0	1	0	0	0	0	0
0	0	0	1	0	0	0	0	0
0	0	0	1	0	0	0	0	0
0	0	0	1	0	0	0	0	0
0	0	0	1	0	0	0	0	0
0	0	0	1	0	0	0	0	0
0	0	0	1	0	0	0	0	0
0	0	0	1	0	0	0	0	0
0	0	0	1	0	0	0	0	0
0	0	0	1	0	0	0	0	0
0	0	0	1	0	0	0	0	0
0	0	0	1	0	0	0	0	0
0	0	0	0	0	0	1	0	1
0	0	0	0	0	0	0	0	0
0	0	0	0	0	0	0	0	0
0	0	0	0	0	0	0	0	0
0	0	0	0	0	0	0	0	0
0	0	0	0	0	0	0	0	0
0	0	0	0	0	0	0	0	0
0	0	0	0	0	0	0	0	0
0	0	0	0	0	0	0	0	0
0	0	0	0	0	0	0	0	0
0	0	0	0	0	0	0	0	0
0	0	0	0	0	0	0	0	0
0	0	0	0	0	0	0	0	0
0	0	0	0	1	0	0	0	0
0	0	0	0	1	0	0	0	0
0	0	0	0	1	0	0	0	0
0	0	0	0	1	0	0	0	0
0	0	0	0	1	0	0	0	0
0	0	0	0	1	0	0	0	0
0	0	0	0	1	0	0	0	0
0	0	0	0	0	0	0	0	1
0	0	0	0	0	0	0	0	0

0	0	0	0	0	0	0	0	0
0	0	0	0	0	0	0	0	0
0	0	0	0	0	0	0	0	0
0	0	0	0	0	0	0	0	0
0	0	0	0	0	0	0	0	0
0	0	0	0	0	0	0	0	0
0	0	0	0	0	0	0	0	0
0	0	0	0	0	0	0	0	0
0	0	0	0	0	0	0	0	0
0	0	0	0	0	0	0	0	0
0	0	0	0	0	0	0	0	0
0	0	0	0	0	0	0	0	0
0	0	0	0	0	0	0	0	0
0	0	0	0	1	0	0	0	0
0	0	0	0	0	0	0	0	0
0	0	0	0	0	0	0	0	0
0	0	0	0	0	0	0	0	0
0	0	0	0	0	0	0	0	1
0	0	0	0	0	0	0	0	0
0	0	0	0	0	0	0	0	0
0	0	0	0	0	0	0	0	0
0	0	0	0	0	0	0	0	0
0	0	1	0	0	0	0	0	0
0	0	1	0	0	0	0	0	0
1	0	0	0	0	0	0	0	0
0	0	0	0	0	0	0	0	0
0	0	0	0	0	0	0	0	0
0	0	0	0	0	0	0	0	0
0	0	0	0	0	0	0	0	0
0	0	0	0	0	0	0	0	0
0	0	0	0	0	0	0	0	0
0	0	0	0	0	0	0	0	0
0	1	0	0	0	0	0	0	0
0	0	0	0	0	0	0	0	0
0	0	0	0	0	0	0	0	0
0	0	0	0	0	0	0	0	0
0	0	0	0	1	0	0	0	0
1	0	0	0	0	0	0	0	0
0	0	0	0	0	0	0	0	0
0	0	0	0	0	0	0	1	0
0	1	0	0	0	0	0	0	0
0	0	0	0	0	0	0	0	0
0	0	0	0	0	0	0	0	0
0	0	0	0	0	0	0	0	0
0	0	0	0	0	0	0	0	0
0	0	0	0	0	0	0	0	0
0	0	0	0	0	0	0	0	0
0	0	0	0	0	0	0	0	0
0	0	0	0	0	0	0	0	0
0	0	0	0	0	0	0	0	0

0	0	0	0	0	0	0	0	0
0	0	0	0	0	0	0	0	0
0	0	0	0	0	0	0	0	0
0	0	0	0	0	0	0	0	0
0	0	0	0	0	0	0	0	0
0	0	0	0	0	0	0	0	0
0	0	0	0	0	0	0	0	0
0	0	0	0	0	0	0	0	0
0	0	0	0	0	0	0	0	0
0	0	0	0	0	0	0	0	0
0	0	0	0	0	0	0	0	0
0	0	0	0	0	0	0	0	0
0	0	0	0	0	0	0	0	0
0	0	0	0	0	0	0	0	0
0	0	0	0	0	0	0	0	0
0	0	0	0	0	0	0	0	0
0	0	0	0	0	0	0	0	0
0	0	0	0	0	0	0	0	0
0	0	0	0	0	0	0	0	0
0	0	0	0	0	0	0	0	0
0	0	0	1	0	0	0	0	0
0	0	0	0	0	0	0	0	0
0	0	0	0	0	0	0	0	0
0	0	0	0	0	0	0	0	0
0	0	0	0	0	0	0	0	0
0	0	0	0	0	0	0	0	0
0	0	0	0	0	0	0	0	0
0	0	0	0	0	0	0	0	0
0	0	0	0	0	0	0	0	0
0	0	0	0	0	0	0	0	0
0	0	0	0	0	0	0	0	0
0	0	0	0	0	0	0	0	0
0	0	0	0	0	0	0	0	0
0	0	0	0	0	0	0	0	0
0	0	0	0	0	0	0	0	0
0	0	0	0	0	0	0	0	0
0	0	0	0	0	0	0	0	0
0	0	0	0	0	0	0	0	0
0	0	0	0	0	0	0	0	0
0	0	0	0	0	0	0	0	0
0	0	0	0	0	0	0	1	0
0	0	0	0	0	0	0	0	0
0	0	0	0	0	0	0	0	0
0	0	0	0	0	0	0	0	0
0	0	0	0	0	0	0	0	0
0	0	0	0	0	0	0	0	0
0	0	0	0	0	0	0	0	0
0	0	0	0	0	0	0	0	0
0	0	0	0	0	0	0	0	0
0	0	0	0	0	0	0	0	0

0	0	0	0	0	0	0	0	0
0	0	0	0	0	0	0	0	0
0	0	0	0	0	0	0	0	0
0	0	0	0	0	0	0	1	0
0	0	0	0	0	0	0	0	0
0	0	0	0	0	0	0	1	0
0	0	0	0	0	0	0	1	0
0	0	0	0	0	0	0	0	0
0	0	0	0	0	0	0	1	0
0	0	0	0	0	0	0	0	0
0	0	0	0	0	0	0	0	0
0	0	0	0	0	0	0	0	0
0	0	0	0	0	0	0	0	0
1	0	0	0	0	0	0	0	0
1	0	0	0	0	0	0	0	0
1	0	0	0	0	0	0	0	0
0	1	0	0	0	0	0	0	0
0	1	0	0	0	0	0	0	0
0	1	0	0	0	0	0	0	0
0	1	0	0	0	0	0	0	0
0	1	0	0	0	0	0	0	0
0	1	0	0	0	0	0	0	0
0	1	0	0	0	0	0	0	0
0	1	0	0	0	0	0	0	0
0	1	0	0	0	0	0	0	0
0	1	0	0	0	0	0	0	0
0	1	0	0	0	0	0	0	0
0	1	0	0	0	0	0	0	0
0	1	0	0	0	0	0	0	0
0	1	0	0	0	0	0	0	0
0	1	0	0	0	0	0	0	0
0	1	0	0	0	0	0	0	0
0	1	0	0	0	0	0	0	0
0	1	0	0	0	0	0	0	0
0	1	0	0	0	0	0	0	0
0	1	0	0	0	0	0	0	0
0	1	0	0	0	0	0	0	0
0	1	0	0	0	0	0	0	0
0	1	0	0	0	0	0	0	0
0	1	0	0	0	0	0	0	0
0	1	0	0	0	0	0	0	0
0	1	0	0	0	0	0	0	0
0	1	0	0	0	0	0	0	0
0	1	0	0	0	0	0	0	0
0	1	0	0	0	0	0	0	0
0	1	0	0	0	0	0	0	0
0	1	0	0	0	0	0	0	0
0	1	0	0	0	0	0	0	0
0	1	0	0	0	0	0	0	0
0	1	0	0	0	0	0	0	0

0	1	0	0	0	0	0	0	0
0	1	0	0	0	0	0	0	0
0	1	0	0	0	0	0	0	0
0	1	0	0	0	0	0	0	0
0	1	0	0	0	0	0	0	0
0	1	0	0	0	0	0	0	0
0	1	0	0	0	0	0	0	0
0	1	0	0	0	0	0	0	0
0	1	0	0	0	0	0	0	0
0	1	0	0	0	0	0	0	0
0	1	0	0	0	0	0	0	0
0	1	0	0	0	0	0	0	0
0	1	0	0	0	0	0	0	0
0	1	0	0	0	0	0	0	0
0	1	0	0	0	0	0	0	0
0	1	0	0	0	0	0	0	0
0	1	0	0	0	0	0	0	0
0	1	0	0	0	0	0	0	0
0	1	0	0	0	0	0	0	0
0	0	1	0	0	0	0	0	0
0	0	1	0	0	0	0	0	0
0	0	1	0	0	0	0	0	0
0	0	1	0	0	0	0	0	0
0	0	1	0	0	0	0	0	0
0	0	1	0	0	0	0	0	0
0	0	1	0	0	0	0	0	0
0	0	1	0	0	0	0	0	0
0	0	0	0	1	0	0	0	0
0	0	0	1	0	0	0	0	0
0	0	0	1	0	0	0	0	0
0	0	0	1	0	0	0	0	0
0	0	0	1	0	0	0	0	0
0	0	0	1	0	0	0	0	0
0	0	0	1	0	0	0	0	0
0	0	0	1	0	0	0	0	0
0	0	0	1	0	0	0	0	0
0	0	0	0	1	0	0	0	0
0	0	0	0	1	0	0	0	0
0	0	0	0	1	0	0	0	0
0	0	0	0	1	0	0	0	0
0	0	0	0	1	0	0	0	0
0	0	0	0	1	0	0	0	0
0	0	0	0	0	1	0	0	0
0	0	0	0	0	0	0	1	0
0	0	0	0	0	0	0	0	0
0	0	0	0	0	0	0	1	0
0	0	0	0	0	0	0	1	0
0	0	0	0	0	0	0	1	0
0	0	0	0	0	0	0	0	1
0	0	0	0	0	0	0	0	1

0	0	0	0	0	0	0	0	0
0	0	0	0	0	0	0	0	1
0	0	0	0	0	0	0	0	1
0	0	0	0	0	0	0	0	1
0	0	0	0	0	0	0	0	0
0	0	0	0	0	0	0	0	1
0	0	0	0	0	0	0	0	1
0	0	0	0	0	0	0	0	1
0	0	0	0	0	0	0	0	1
0	0	0	0	0	0	0	0	1
0	0	0	0	0	0	0	0	1
0	0	0	0	0	0	0	0	1
0	0	0	0	0	0	0	0	1
0	0	0	0	0	0	0	0	1
0	0	0	0	0	0	0	0	1
0	0	0	0	0	0	0	0	1
0	0	0	0	0	0	0	0	1
0	0	0	0	0	0	0	0	1
0	0	0	0	0	0	0	0	1
0	0	0	0	0	0	0	0	1
0	0	0	0	0	0	0	0	1
0	0	0	0	0	0	0	0	1
0	0	0	0	0	0	0	0	1
0	0	0	0	0	0	0	0	1
0	0	0	0	0	0	0	0	0
0	0	0	0	0	0	0	0	0
0	0	0	0	0	0	0	0	0
0	0	0	0	0	0	0	0	0
0	0	0	0	0	0	0	0	0
0	0	0	0	0	0	0	0	0
0	0	0	0	0	0	0	0	0
0	0	0	0	0	0	0	0	0
0	0	0	0	0	0	0	0	0
0	0	0	0	0	0	0	0	0
0	0	0	0	0	0	0	0	0
0	0	0	0	0	0	0	0	0
0	0	0	0	0	0	0	0	0
0	0	0	0	0	0	0	0	0
0	0	0	0	0	0	0	0	0
0	0	0	0	0	0	0	0	0
0	0	0	0	0	0	0	0	0
0	0	0	0	1	0	0	0	0
0	0	0	0	0	0	0	0	1
0	0	0	0	1	0	0	0	0
0	0	0	0	0	0	0	0	0
0	0	0	0	0	0	0	0	0
0	0	0	0	1	0	0	0	0
0	0	0	0	1	0	0	0	0

SMU	UC23022	XH1056	XH858
1	1	1	1
1	1	1	1
1	1	1	1
1	1	1	1
1	1	1	1
1	1	1	1
1	1	1	1
1	1	1	1
1	1	1	1
1	1	1	1
1	1	1	1
1	1	1	1
1	1	1	1
1	1	1	1
1	1	1	1
1	1	1	1
1	1	1	1
1	1	1	1
1	1	1	1
1	1	1	1
1	1	1	1
1	1	1	1
1	1	1	1
1	1	1	1
1	1	1	1
1	1	1	1
1	1	1	1
1	1	1	1
1	1	1	1
1	1	1	1
1	1	1	1
1	1	1	1
1	1	1	1
1	1	1	1
1	1	1	1
1	1	1	1
1	1	1	1
1	1	1	1
1	1	1	1
1	1	1	1
1	1	1	1
1	1	1	1
1	1	1	1
1	1	1	1
1	1	1	1
1	1	1	1
1	1	1	1
1	1	1	1

1	1	1	1
1	1	1	1
1	1	1	1
1	1	1	1
1	1	1	1
1	1	1	1
1	1	1	1
1	1	1	1
1	1	1	1
1	1	1	1
1	1	1	1
1	1	1	1
1	1	1	1
1	1	1	1
1	1	1	1
1	1	1	1
1	1	1	1
1	1	1	1
1	1	1	1
1	1	1	1
1	1	1	1
1	1	1	1
1	1	1	1
1	1	1	1
1	1	1	1
1	1	1	1
1	1	1	1
1	1	1	1
1	1	1	1
1	1	1	1
1	1	1	1
1	1	1	1
1	1	1	1
1	1	1	1
1	1	1	1
1	1	1	1
1	1	1	1
1	1	1	1
1	1	1	1
1	1	1	1
1	1	1	1
1	1	1	1
1	1	1	1
1	1	1	1
1	1	1	1
1	1	1	1
1	1	1	1
1	1	1	1
1	1	1	1
1	1	1	1

1	1	1	1
1	1	1	1
1	1	1	1
1	1	1	1
1	1	1	1
1	1	1	1
1	1	1	1
1	1	1	1
1	1	1	1
1	1	1	1
1	1	1	1
1	1	1	1
1	1	1	1
1	1	1	1
1	1	1	1
1	1	1	1
1	1	1	1
1	1	1	1
1	1	1	1
1	1	1	1
1	1	1	1
1	1	1	1
1	1	1	1
1	1	1	1
1	1	1	1
1	1	1	1
1	1	1	1
1	1	1	1
1	1	1	1
1	1	1	1
1	1	1	1
1	1	1	1
1	1	1	1
1	1	1	1
1	1	1	1
1	1	1	1
1	1	1	1
1	1	1	1
1	1	1	1
1	1	1	1
1	1	1	1
1	1	1	1
1	1	1	1
1	1	1	1
1	1	1	1
1	1	1	1
1	1	1	1
1	1	1	1
1	1	1	1
1	1	1	1

1	1	1	1
1	1	1	1
1	1	1	1
1	1	1	1
1	1	1	1
1	1	1	1
1	1	1	1
1	1	1	1
1	1	1	1
1	1	1	1
1	1	1	1
1	1	1	1
1	1	1	1
1	1	1	1
1	1	1	1
1	1	1	1
1	1	1	1
1	1	1	1
1	1	1	1
1	1	1	1
1	1	1	1
1	1	1	1
1	1	1	1
1	1	1	1
1	1	1	1
1	1	1	1
1	1	1	1
1	1	1	1
1	1	1	1
1	1	1	1
1	1	1	1
1	1	1	1
1	1	1	1
1	1	1	1
1	1	1	1
1	1	1	1
1	1	1	1
1	1	1	1
1	1	1	1
1	1	1	1
1	1	1	1
1	1	1	1
1	1	1	1
1	1	1	1
1	1	1	1
1	1	1	1
1	1	1	1
1	1	1	1
1	1	1	1
1	1	1	1

1	1	1	1
1	1	1	1
1	1	1	1
1	1	1	1
1	1	1	1
1	1	1	1
1	1	1	1
1	1	1	1
1	1	1	1
1	1	1	1
1	1	1	1
1	1	1	1
1	1	1	1
1	1	1	1
1	1	1	1
1	1	1	1
1	1	1	1
1	1	1	1
1	1	1	1
1	1	1	1
1	1	1	1
1	1	1	1
1	1	1	1
1	1	1	1
1	1	1	1
1	1	1	1
1	1	1	1
1	1	1	1
1	1	1	1
1	1	1	1
1	1	1	1
1	1	1	1
1	1	1	1
1	1	1	1
1	1	1	1
1	1	1	1
1	1	1	1
1	1	1	1
1	1	1	1
1	1	1	1
1	1	1	1
1	1	1	1
1	1	1	1
1	1	1	1
1	1	1	1
1	1	1	1
1	1	1	1
1	1	1	1
1	1	1	1
1	1	1	1

1	1	1	1
1	1	1	1
1	1	1	1
1	1	1	1
1	1	1	1
1	1	1	1
1	1	1	1
1	1	1	1
1	1	1	1
1	1	1	1
1	1	1	1
1	1	1	1
1	1	1	1
1	1	1	1
1	1	1	1
1	1	1	1
1	1	1	1
1	1	1	1
1	1	1	1
1	1	1	1
1	1	1	1
1	1	1	1
1	1	1	1
1	1	1	1
1	1	1	1
1	1	1	1
1	1	1	1
1	1	1	1
1	1	1	1
1	1	1	1
1	1	1	1
1	1	1	1
1	1	1	1
1	1	1	1
1	1	1	1
1	1	1	1
1	1	1	1
1	1	1	1
1	1	1	1
1	1	1	1
1	1	1	1
1	1	1	1
1	1	1	1
1	1	1	1
1	1	1	1
1	1	1	1
1	1	1	1
1	1	1	1
1	1	1	1
1	1	1	1

1	1	1	1
1	1	1	1
1	1	1	1
1	1	1	1
1	1	1	1
1	1	1	1
1	1	1	1
1	1	1	1
1	1	1	1
1	1	1	1
1	1	1	1
1	1	1	1
1	1	1	1
1	1	1	1
1	1	1	1
1	1	1	1
1	1	1	1
1	1	1	1
1	1	1	1
1	1	1	1
1	1	1	1
1	1	1	1
1	1	1	1
1	1	1	1
1	1	1	1
1	1	1	1
1	1	1	1
1	1	1	1
1	1	1	1
1	1	1	1
1	1	1	1
1	1	1	1
1	1	1	1
1	1	1	1
1	1	1	1
1	1	1	1
1	1	1	1
1	1	1	1
1	1	1	1
1	1	1	1
1	1	1	1
1	1	1	1
1	1	1	1
1	1	1	1
1	1	1	1
1	1	1	1
1	1	1	1
1	1	1	1
1	1	1	1
1	1	1	1

1	1	1	1
1	1	1	1
1	1	1	1
1	1	1	1
1	1	1	1
1	1	1	1
1	1	1	1
1	1	1	1
1	1	1	1
1	1	1	1
1	1	1	1
1	1	1	1
1	1	1	1
1	1	1	1
1	1	1	1
1	1	1	1
1	1	1	1
1	1	1	1
1	1	1	1
1	1	1	1
1	1	1	1
1	1	1	1
1	1	1	1
1	1	1	1
1	1	1	1
1	1	1	1
1	1	1	1
1	1	1	1
1	1	1	1
1	1	1	1
1	1	1	1
1	1	1	1
1	1	1	1
1	1	1	1
1	1	1	1
1	1	1	1
1	1	1	1
1	1	1	1
1	1	1	1
1	1	1	1
1	1	1	1
1	1	1	1
1	1	1	1
1	1	1	1
1	1	1	1
1	1	1	1
1	1	1	1
1	1	1	1
1	1	1	1
1	1	1	1

1	1	1	1
1	1	1	1
1	1	1	1
1	1	1	1
1	1	1	1
1	1	1	1
1	1	1	1
1	1	1	1
1	1	1	1
1	1	1	1
1	1	1	1
1	1	1	1
1	1	1	1
1	1	1	1
1	1	1	1
1	1	1	1
1	1	1	1
1	1	1	1
1	1	1	1
1	1	1	1
1	1	1	1
1	1	1	1
1	1	1	1
1	1	1	1
1	1	1	1
1	1	1	1
1	1	1	1
1	1	1	1
1	1	1	1
1	1	1	1
1	1	1	1
1	1	1	1
1	1	1	1
1	1	1	1
1	1	1	1
1	1	1	1
1	1	1	1
1	1	1	1
1	1	1	1
1	1	1	1
1	1	1	1
1	1	1	1
1	1	1	1
1	1	1	1
1	1	1	1
1	1	1	1
1	1	1	1
1	1	1	1
1	1	1	1
1	1	1	1

1	1	1	1
1	1	1	1
1	1	1	1
1	1	1	1
1	1	1	1
1	1	1	1
1	1	1	1
1	1	1	1
1	1	1	1
1	1	1	1
1	1	1	1
1	1	1	1
1	1	1	1
1	1	1	1
1	1	1	1
1	1	1	1
1	1	1	1
1	1	1	1
1	1	1	1
1	1	1	1
1	1	1	1
1	1	1	1
1	1	1	1
1	1	1	1
1	1	1	1
1	1	1	1
1	1	1	1
1	1	1	1
1	1	1	1
1	1	1	1
1	1	1	1
1	1	1	1
1	1	1	1
1	1	1	1
1	1	1	1
1	1	1	1
1	1	1	1
1	1	1	1
1	1	1	1
1	1	1	1
1	1	1	1
1	1	1	1
1	1	1	1
1	1	1	1
1	1	1	1
1	1	1	1
1	1	1	1
1	1	1	1
1	1	1	1
1	1	1	1

1	1	1	1
1	1	1	1
1	1	1	1
1	1	1	1
1	1	1	1
1	1	1	1
1	1	1	1
1	1	1	1
1	1	1	1
1	1	1	1
1	1	1	1
1	1	1	1
1	1	1	1
1	1	1	1
1	1	1	1
1	1	1	1
1	1	1	1
1	1	1	1
1	1	1	1
1	1	1	1
1	1	1	1
1	1	1	1
1	1	1	1
1	1	1	1
1	1	1	1
1	1	1	1
1	1	1	1
1	1	1	1
1	1	1	1
1	1	1	1
1	1	1	1
1	1	1	1
1	1	1	1
1	1	1	1
1	1	1	1
1	1	1	1
1	1	1	1
1	1	1	1
1	1	1	1
1	1	1	1
1	1	1	1
1	1	1	1
1	1	1	1
1	1	1	1
1	1	1	1
1	1	1	1
1	1	1	1
1	1	1	1
1	1	1	1
1	1	1	1

1	1	1	1
1	1	1	1
1	1	1	1
1	1	1	1
1	1	1	1
1	1	1	1
1	1	1	1
1	1	1	1
1	1	1	1
1	1	1	1
1	1	1	1
1	1	1	1
1	1	1	1
1	1	1	1
1	1	1	1
1	1	1	1
1	1	1	1
1	1	1	1
1	1	1	1
1	1	1	1
1	1	1	1
1	1	1	1
1	1	1	1
1	1	1	1
1	1	1	1
1	1	1	1
1	1	1	1
1	1	1	1
1	1	1	1
1	1	1	1
1	1	1	1
1	1	1	1
1	1	1	1
1	1	1	1
1	1	1	1
1	1	1	1
1	1	1	1
1	1	1	1
1	1	1	1
1	1	1	1
1	1	1	1
1	1	1	1
1	1	1	1
1	1	1	1
1	1	1	1
1	1	1	1
1	1	1	1
1	1	1	1
1	1	1	1
1	1	1	1

1	1	1	1
1	1	1	1
1	1	1	1
1	1	1	1
1	1	1	1
1	1	1	1
1	1	1	1
1	1	1	1
1	1	1	1
1	1	1	1
1	1	1	1
1	1	1	1
1	1	1	1
1	1	1	1
1	1	1	1
1	1	1	1
1	1	1	1
1	1	1	1
1	1	1	1
1	1	1	1
1	1	1	1
1	1	1	1
1	1	1	1
1	1	1	1
1	1	1	1
1	1	1	1
1	1	1	1
1	1	1	1
1	1	1	1
1	1	1	1
1	1	1	1
1	1	1	1
1	1	1	1
1	1	1	1
1	1	1	1
1	1	1	1
1	1	1	1
1	1	1	1
1	1	1	1
1	1	1	1
1	1	1	1
1	1	1	1
1	1	1	1
1	1	1	1
1	1	1	1
1	1	1	1
1	1	1	1
1	1	1	1
1	1	1	1
1	1	1	1

1	1	1	1
1	1	1	1
1	1	1	1
1	1	1	1
1	1	1	1
1	1	1	1
1	1	1	1
1	1	1	1
1	1	1	1
1	1	1	1
1	1	1	1
1	1	1	1
1	1	1	1
1	1	1	1
1	1	1	1
1	1	1	1
1	1	1	1
1	1	1	1
1	1	1	1
1	1	1	1
1	1	1	1
1	1	1	1
1	1	1	1
1	1	1	1
1	1	1	1
1	1	1	1
1	1	1	1
1	1	1	1
1	1	1	1
1	1	1	1
1	1	1	1
1	1	1	1
1	1	1	1
1	1	1	1
1	1	1	1
1	1	1	1
1	1	1	1
1	1	1	1
1	1	1	1
1	1	1	1
1	1	1	1
1	1	1	1
1	1	1	1
1	1	1	1
1	1	1	1
1	1	1	1
1	1	1	1
1	1	1	1
1	1	1	1
1	1	1	1

1	1	1	1
1	1	1	1
1	1	1	1
1	1	1	1
1	1	1	1
1	1	1	1
1	1	1	1
1	1	1	1
1	1	1	1
1	1	1	1
1	1	1	1
1	1	1	1
1	1	1	1
1	1	1	1
1	1	1	1
1	1	1	1
1	1	1	1
1	1	1	1
1	1	1	1
1	1	1	1
1	1	1	1
1	1	1	1
1	1	1	1
1	1	1	1
1	1	1	1
1	1	1	1
1	1	1	1
1	1	1	1
1	1	1	1
1	1	1	1
1	1	1	1
1	1	1	1
1	1	1	1
1	1	1	1
1	1	1	1
1	1	1	1
1	1	1	1
1	1	1	1
1	1	1	1
1	1	1	1
1	1	1	1
1	1	1	1
1	1	1	1
1	1	1	1
1	1	1	1
1	1	1	1
1	1	1	1
1	1	1	1
1	1	1	1
1	1	1	1

1	1	1	1
1	1	1	1
1	1	1	1
1	1	1	1
1	1	1	1
1	1	1	1
1	1	1	1
1	1	1	1
1	1	1	1
1	1	1	1
1	1	1	1
1	1	1	1
1	1	1	1
1	1	1	1
1	1	1	1
1	1	1	1
1	1	1	1
1	1	1	1
1	1	1	1
1	1	1	1
1	1	1	1
1	1	1	1
1	1	1	1
1	1	1	1
1	1	1	1
1	1	1	1
1	1	1	1
1	1	1	1
1	1	1	1
1	1	1	1
1	1	1	1
1	1	1	1
1	1	1	1
1	1	1	1
1	1	1	1
1	1	1	1
1	1	1	1
1	1	1	1
1	1	1	1
1	1	1	1
1	1	1	1
1	1	1	1
1	1	1	1
1	1	1	1
1	1	1	1
1	1	1	1
1	1	1	1
1	1	1	1
1	1	1	1
1	1	1	1

1	1	1	1
1	1	1	1
1	1	1	1
1	1	1	1
1	1	1	1
1	1	1	1
1	1	1	1
1	1	1	1
1	1	1	1
1	1	1	1
1	1	1	1
1	1	1	1
1	1	1	1
1	1	1	1
1	1	1	1
1	1	1	1
1	1	1	1
1	1	1	1
1	1	1	1
1	1	1	1
1	1	1	1
1	1	1	1
1	1	1	1
1	1	1	1
1	1	1	1
1	1	1	1
1	1	1	1
1	1	1	1
1	1	1	1
1	1	1	1
1	1	1	1
1	1	1	1
1	1	1	1
1	1	1	1
1	1	1	1
1	1	1	1
1	1	1	1
1	1	1	1
1	1	1	1
1	1	1	1
1	1	1	1
1	1	1	1
1	1	1	1
1	1	1	1
1	1	1	1
1	1	1	1
1	1	1	1
1	1	1	1
1	1	1	1
1	1	1	1

1	1	1	1
1	1	1	1
1	1	1	1
1	1	1	1
1	1	1	1
1	1	1	1
1	1	1	1
1	1	1	1
1	1	1	1
1	1	1	1
1	1	1	1
1	1	1	1
1	1	1	1
1	1	1	1
1	1	1	1
1	1	1	1
1	1	1	1
1	1	1	1
1	1	1	1
1	1	1	1
1	1	1	1
1	1	1	1
1	1	1	1
1	1	1	1
1	1	1	1
1	1	1	1
1	1	1	1
1	1	1	1
1	1	1	1
1	1	1	1
1	1	1	1
1	1	1	1
1	1	1	1
1	1	1	1
1	1	1	1
1	1	1	1
1	1	1	1
1	1	1	1
1	1	1	1
1	1	1	1
1	1	1	1
1	1	1	1
1	1	1	1
1	1	1	1
1	1	1	1
1	1	1	1
1	1	1	1
1	1	1	1
1	1	1	1
1	1	1	1

1	1	1	1
1	1	1	1
1	1	1	1
1	1	1	1
1	1	1	1
1	1	1	1
1	1	1	1
1	1	1	1
1	1	1	1
1	1	1	1
1	1	1	1
1	1	1	1
1	1	1	1
1	1	1	1
1	1	1	1
1	1	1	1
1	1	1	1
1	1	1	1
1	1	1	1
1	1	1	1
1	1	1	1
1	1	1	1
1	1	1	1
1	1	1	1
1	1	1	1
1	1	1	1
1	1	1	1
1	1	1	1
1	1	1	1
1	1	1	1
1	1	1	1
1	1	1	1
1	1	1	1
1	1	1	1
1	1	1	1
1	1	1	1
1	1	1	1
1	1	1	1
1	1	1	1
1	1	1	1
1	1	1	1
1	1	1	1
1	1	1	1
1	1	1	1
1	1	1	1
1	1	1	1
1	1	1	1
1	1	1	1
1	1	1	1
1	1	1	1

1	1	1	1
1	1	1	1
1	1	1	1
1	1	1	1
1	1	1	1
1	1	1	1
1	1	1	1
1	1	1	1
1	1	1	1
1	1	1	1
1	1	1	1
1	1	1	1
1	1	1	1
1	1	1	1
1	1	1	1
1	1	1	1
1	1	1	1
1	1	1	1
1	1	1	1
1	1	1	1
1	1	1	1
1	1	1	1
1	1	1	1
1	1	1	1
1	1	1	1
1	1	1	1
1	1	1	1
1	1	1	1
1	1	1	1
1	1	1	1
1	1	1	1
1	1	1	1
1	1	1	1
1	1	1	1
1	1	1	1
1	1	1	1
1	1	1	1
1	1	1	1
1	1	1	1
1	1	1	1
1	1	1	1
1	1	1	1
1	1	1	1
1	1	1	1
1	1	1	1
1	1	1	1
1	1	1	1
1	1	1	1
1	1	1	1
1	1	1	1

1	1	1	1
1	1	1	1
1	1	1	1
1	1	1	1
1	1	1	1
1	1	1	1
1	1	1	1
1	1	1	1
1	1	1	1
1	1	1	1
1	1	1	1
1	1	1	1
1	1	1	1
1	1	1	1
1	1	1	1
1	1	1	1
1	1	1	1
1	1	1	1
1	1	1	1
1	1	1	1
1	1	1	1
1	1	1	1
1	1	1	1
1	1	1	1
1	1	1	1
1	1	1	1
1	1	1	1
1	1	1	1
1	1	1	1
1	1	1	1
1	1	1	1
1	1	1	1
1	1	1	1
1	1	1	1
1	1	1	1
1	1	1	1
1	1	1	1
1	1	1	1
1	1	1	1
1	1	1	1
1	1	1	1
1	1	1	1
1	1	1	1
1	1	1	1
1	1	1	1
1	1	1	1
1	1	1	1
1	1	1	1
1	1	1	1
1	1	1	1

1	1	1	1
1	1	1	1
1	1	1	1
1	1	1	1
1	1	1	1
1	1	1	1
1	1	1	1
1	1	1	1
1	1	1	1
1	1	1	1
1	1	1	1
1	1	1	1
1	1	1	1
1	1	1	1
1	1	1	1
1	1	1	1
1	1	1	1
1	1	1	1
1	1	1	1
1	1	1	1
1	1	1	1
1	1	1	1
1	1	1	1
1	1	1	1
1	1	1	1
1	1	1	1
1	1	1	1
1	1	1	1
1	1	1	1
1	1	1	1
1	1	1	1
1	1	1	1
1	1	1	1
1	1	1	1
1	1	1	1
1	1	1	1
1	1	1	1
1	1	1	1
1	1	1	1
1	1	1	1
1	1	1	1
1	1	1	1
1	1	1	1
1	1	1	1
1	1	1	1
1	1	1	1
1	1	1	1
1	1	1	1
1	1	1	1
1	1	1	1

1	1	1	1
1	1	1	1
1	1	1	1
1	1	1	1
1	1	1	1
1	1	1	1
1	1	1	1
1	1	1	1
1	1	1	1
1	1	1	1
1	1	1	1
1	1	1	1
1	1	1	1
1	1	1	1
1	1	1	1
1	1	1	1
1	1	1	1
1	1	1	1
1	1	1	1
1	1	1	1
1	1	1	1
1	1	1	1
1	1	1	1
1	1	1	1
1	1	1	1
1	1	1	1
1	1	1	1
1	1	1	1
1	1	1	1
1	1	1	1
1	1	1	1
1	1	1	1
1	1	1	1
1	1	1	1
1	1	1	1
1	1	1	1
1	1	1	1
1	1	1	1
1	1	1	1
1	1	1	1
1	1	1	1
1	1	1	1
1	1	1	1
1	1	1	1
1	1	1	1
1	1	1	1
1	1	1	1
1	1	1	1
1	1	1	1
1	1	1	1

1	1	1	1
1	1	1	1
1	1	1	1
1	1	1	1
1	1	1	1
1	1	1	1
1	1	1	1
1	1	1	1
1	1	1	1
1	1	1	1
1	1	1	1
1	1	1	1
1	1	1	1
1	1	1	1
1	1	1	1
1	1	1	1
1	1	1	1
1	1	1	1
1	1	1	1
1	1	1	1
1	1	1	1
1	1	1	1
1	1	1	1
1	1	1	1
1	1	1	1
1	1	1	1
1	1	1	1
1	1	1	1
1	1	1	1
1	1	1	1
1	1	1	1
1	1	1	1
1	1	1	1
1	1	1	1
1	1	1	1
1	1	1	1
1	1	1	1
1	1	1	1
1	1	1	1
1	1	1	1
1	1	1	1
1	1	1	1
1	1	1	1
1	1	1	1
1	1	1	1
1	1	1	1
1	1	1	1
1	1	1	1
1	1	1	1
1	1	1	1

1	1	1	1
1	1	1	1
1	1	1	1
1	1	1	1
1	1	1	1
1	1	1	1
1	1	1	1
1	1	1	1
1	1	1	1
1	1	1	1
1	1	1	1
1	1	1	1
1	1	1	1
1	1	1	1
1	1	1	1
1	1	1	1
1	1	1	1
1	1	1	1
1	1	1	1
1	1	1	1
1	1	1	1
1	1	1	1
1	1	1	1
1	1	1	1
1	1	1	1
1	1	1	1
1	1	1	1
1	1	1	1
1	1	1	1
1	1	1	1
1	1	1	1
1	1	1	1
1	1	1	1
1	1	1	1
1	1	1	1
1	1	1	1
1	1	1	1
1	1	1	1
1	1	1	1
1	1	1	1
1	1	1	1
1	1	1	1
1	1	1	1
1	1	1	1
1	1	1	1
1	1	1	1
1	1	1	1
1	1	1	1
1	1	1	1
1	1	1	1

1	1	1	1
1	1	1	1
1	1	1	1
1	1	1	1
1	1	1	1
1	1	1	1
1	1	1	1
1	1	1	1
1	1	1	1
1	1	1	1
1	1	1	1
1	1	1	1
1	1	1	1
1	1	1	1
1	1	1	1
1	1	1	1
1	1	1	1
1	1	1	1
1	1	1	1
1	1	1	1
1	1	1	1
1	1	1	1
1	1	1	1
1	1	1	1
1	1	1	1
1	1	1	1
1	1	1	1
1	1	1	1
1	1	1	1
1	1	1	1
1	1	1	1
1	1	1	1
1	1	1	1
1	1	1	1
1	1	1	1
1	1	1	1
1	1	1	1
1	1	1	1
1	1	1	1
1	1	1	1
1	1	1	1
1	1	1	1
1	1	1	1
1	1	1	1
1	1	1	1
1	1	1	1
1	1	1	1
1	1	1	1
1	1	1	1
1	1	1	1

1	1	1	1
1	1	1	1
1	1	1	1
1	1	1	1
1	1	1	1
1	1	1	1
1	1	1	1
1	1	1	1
1	1	1	1
1	1	1	1
1	1	1	1
1	1	1	1
1	1	1	1
1	1	1	1
1	1	1	1
1	1	1	1
1	1	1	1
1	1	1	1
1	1	1	1
1	1	1	1
1	1	1	1
1	1	1	1
1	1	1	1
1	1	1	1
1	1	1	1
1	1	1	1
1	1	1	1
1	1	1	1
1	1	1	1
1	1	1	1
1	1	1	1
1	1	1	1
1	1	1	1
1	1	1	1
1	1	1	1
1	1	1	1
1	1	1	1
1	1	1	1
1	1	1	1
1	1	1	1
1	1	1	1
1	1	1	1
1	1	1	1
1	1	1	1
1	1	1	1
1	1	1	1
1	1	1	1
1	1	1	1
1	1	1	1
1	1	1	1

1	1	1	1
1	1	1	1
1	1	1	1
1	1	1	1
1	1	1	1
1	1	1	1
1	1	1	1
1	1	1	1
1	1	1	1
1	1	1	1
1	1	1	1
1	1	1	1
1	1	1	1
1	1	1	1
1	1	1	1
1	1	1	1
1	1	1	1
1	1	1	1
1	1	1	1
1	1	1	1
1	1	1	1
1	1	1	1
1	1	1	1
1	1	1	1
1	1	1	1
1	1	1	1
1	1	1	1
1	1	1	1
1	1	1	1
1	1	1	1
1	1	1	1
1	1	1	1
1	1	1	1
1	1	1	1
1	1	1	1
1	1	1	1
1	1	1	1
1	1	1	1
1	1	1	1
1	1	1	1
1	1	1	1
1	1	1	1
1	1	1	1
1	1	1	1
1	1	1	1
1	1	1	1
1	1	1	1
1	1	1	1
1	1	1	1
1	1	1	1

1	1	1	1
1	1	1	1
1	1	1	1
1	1	1	1
1	1	1	1
1	1	1	1
1	1	1	1
1	1	1	1
1	1	1	1
1	1	1	1
1	1	1	1
1	1	1	1
1	1	1	1
1	1	1	1
1	1	1	1
1	1	1	1
1	1	1	1
1	1	1	1
1	1	1	1
1	1	1	1
1	1	1	1
1	1	1	1
1	1	1	1
1	1	1	1
1	1	1	1
1	1	1	1
1	1	1	1
1	1	1	1
1	1	1	1
1	1	1	1
1	1	1	1
1	1	1	1
1	1	1	1
1	1	1	1
1	1	1	1
1	1	1	1
1	1	1	1
1	1	1	1
1	1	1	1
1	1	1	1
0	1	1	1
1	1	1	1
1	1	1	1
1	1	0	1
1	1	1	1
1	1	1	1
1	1	1	1
1	1	1	1
1	1	1	1
1	1	1	1

1	1	1	1
1	1	1	1
1	1	1	1
1	1	1	1
1	1	1	1
1	1	1	1
1	1	1	1
1	1	0	1
1	1	1	1
1	1	1	1
0	1	1	1
1	1	1	1
1	1	1	1
1	1	1	1
1	1	1	1
1	0	1	1
0	1	1	1
1	1	1	1
1	1	1	1
0	1	1	1
1	1	1	1
0	1	1	1
1	1	0	1
1	1	1	1
1	1	1	1
1	1	1	1
1	1	1	1
1	1	1	1
1	1	0	1
1	1	1	1
1	1	1	1
1	1	1	1
0	1	1	1
1	1	0	1
1	1	0	1
1	1	1	1
1	1	1	1
1	1	1	1
1	1	1	1
1	1	1	1
0	1	1	1
0	1	1	1
1	1	1	1
1	1	1	1
1	1	1	1
1	1	1	1
1	0	1	1
1	1	1	1
1	1	1	1
0	1	1	1

1	1	1	1
1	1	1	1
1	1	1	1
0	1	1	1
1	0	1	1
1	1	1	1
1	1	0	0
1	1	1	1
1	0	1	1
1	1	1	1
1	1	0	0
1	1	1	1
0	1	1	1
1	1	1	1
1	1	1	1
1	1	1	1
1	1	1	1
1	1	1	1
1	0	1	1
1	0	1	1
1	1	1	1
1	1	0	0
1	1	0	0
1	1	1	1
1	1	1	1
1	1	1	1
1	1	0	0
1	0	1	1
1	1	1	1
1	1	0	0
1	1	0	0
1	1	0	0
1	1	0	0
1	1	0	0
1	1	1	1
1	1	1	1
1	1	1	1
1	1	1	1
1	1	0	1
1	1	1	1
1	1	1	1
1	1	1	1
1	1	0	1
1	1	0	0
1	1	1	1
1	1	1	1
1	1	1	1
0	1	1	1
1	1	1	1
1	1	1	1

0	1	1	1
1	1	1	1
1	1	1	1
1	1	0	0
0	1	1	1
0	1	1	1
1	1	1	1
1	1	0	0
1	1	1	1
0	1	0	0
1	1	1	1
1	1	0	0
0	1	1	1
1	0	1	1
0	1	0	0
1	1	0	0
1	1	0	0
1	1	1	1
1	1	1	1
0	1	1	1
1	1	1	1
1	1	1	1
1	1	1	1
1	1	1	1
1	1	1	1
0	1	1	1
0	1	1	1
0	1	1	1
1	1	1	1
1	1	0	0
1	1	0	0
1	1	1	1
1	1	0	0
1	1	1	1
1	1	1	1
1	1	0	0
0	1	1	1
1	1	1	1
1	1	1	1
1	1	1	1
1	0	1	1
1	0	1	1
1	0	1	1
1	1	1	1
1	1	0	0
1	1	0	0
1	1	0	0
1	1	1	1
1	1	1	1
1	1	1	1

1	1	1	1
1	1	1	1
1	1	1	1
1	0	1	1
1	0	1	1
1	0	1	1
1	1	1	1
1	1	1	1
0	1	1	1
1	0	1	1
1	1	1	1
0	1	0	0
1	0	1	1
1	0	1	1
1	0	1	1
1	0	1	1
0	0	1	1
0	1	1	1
1	1	1	1
1	0	1	1
1	1	0	0
1	1	1	1
1	0	1	1
0	1	1	1
0	1	0	0
1	0	1	1
0	1	0	0
1	0	1	1
0	1	1	1
1	0	1	1
0	1	0	0
0	1	0	0
0	1	0	0
1	0	1	1
1	0	1	1
0	1	0	0
0	1	1	1
1	1	1	0
1	0	1	1
0	1	1	1
1	1	0	0
0	1	0	0
0	1	0	0
0	1	0	0
0	1	0	0
0	1	1	1
1	1	0	0
0	0	1	1
0	1	1	1
1	1	0	0

0	1	0	0
1	0	1	1
0	1	0	0
1	0	1	1
1	0	1	1
0	0	1	1
0	1	0	0
0	1	0	0
0	1	1	1
1	1	1	1
0	1	0	0
0	1	0	0
0	1	0	0
0	1	0	0
0	1	0	0
0	1	0	0
0	0	1	0
0	1	1	1
0	1	0	0
0	1	0	0
1	1	0	0
0	1	0	0
1	0	0	0
1	1	0	0
0	1	0	0
1	0	0	0
0	1	0	0
0	1	1	1
0	1	0	0
0	1	1	1
1	0	1	1
1	1	0	0
0	1	1	1
1	0	1	1
0	1	0	0
1	1	0	0
0	1	0	0
0	1	0	0
1	1	0	0
0	1	0	0
0	1	0	0
0	1	0	0
1	1	0	0
1	0	1	1
1	0	0	0
1	0	1	1
1	0	1	1
1	0	1	1
1	0	1	1
0	0	0	0

0	1	0	0
1	0	0	0
0	1	0	0
0	1	0	0
0	1	0	0
1	0	1	1
1	0	1	1
1	0	1	1
0	1	1	1
0	1	0	0
0	1	0	0
1	1	1	1
0	1	0	0
1	0	0	0
1	0	1	1
0	1	0	0
1	0	1	1
0	1	0	0
0	1	0	0
0	1	0	0
0	0	1	1
0	1	0	0
0	1	0	0
0	1	0	0
0	1	0	0
1	0	1	1
1	0	1	1
1	0	1	1
1	0	1	1
0	0	0	0
1	0	1	1
0	1	0	0
0	1	1	1
0	1	1	1
0	1	0	0
0	1	0	0
0	1	0	0
0	1	0	0
1	1	0	0
0	0	0	0
0	1	0	0
0	1	0	0
0	1	0	0
0	1	0	0
0	1	0	0
0	1	0	0
0	1	0	0
0	1	0	0
0	1	0	0
0	1	0	0

0	1	0	0
0	1	0	0
0	1	0	0
0	1	0	0
0	1	0	0
0	1	0	0
0	1	0	0
0	1	0	0
0	1	0	0
0	1	0	0
0	1	0	0
0	1	0	0
0	1	0	0
0	1	0	0
0	1	0	0
0	1	0	0
0	1	0	0
0	1	0	0
0	1	0	0
0	1	0	0
0	1	0	0
0	1	0	0
0	1	0	0
0	1	1	1
0	0	1	1
0	0	1	1
0	0	1	1
0	1	0	0
0	1	0	0
1	0	1	1
0	0	0	0
0	1	0	0
0	0	1	1
0	1	0	0
0	0	1	1
1	0	1	1
1	0	0	0
0	0	1	1
0	0	0	0
0	0	1	1
0	0	1	1
0	0	1	1
1	0	0	0
1	0	1	1
0	0	0	0
0	0	1	1
0	0	0	0
0	1	0	0
0	1	1	1
0	1	1	1

0	0	1	1
0	0	0	0
1	0	1	1
1	0	1	1
1	0	1	1
0	0	0	0
1	1	0	0
0	0	0	0
0	0	0	0
0	0	0	0
0	0	0	0
0	0	0	0
1	0	0	0
1	0	0	0
0	0	0	0
0	0	1	1
0	0	0	0
0	0	0	0
0	0	0	0
0	0	1	1
1	0	0	0
0	0	1	1
1	0	0	0
0	0	0	0
0	0	0	0
0	0	0	0
0	0	0	0
0	0	0	0
0	0	1	0
0	0	0	0
0	1	1	1
0	0	0	0
0	0	0	0
0	0	0	0
0	0	1	1
0	0	1	1
1	0	0	0
0	0	0	0
0	0	1	1
0	0	1	1
1	0	1	1
0	0	0	0
0	0	0	0
0	0	0	0
0	1	0	0
0	0	0	0
0	0	0	0
0	0	0	0
0	0	1	0
0	0	1	1

0	0	1	1
0	0	1	1
0	0	1	1
0	0	0	0
0	0	0	0
0	0	0	0
0	0	0	0
0	0	0	0
0	0	0	0
0	0	0	0
0	0	0	0
0	0	0	0
0	0	0	0
0	0	0	0
0	0	0	0
1	0	0	0
0	0	0	0
1	0	0	0
0	0	0	0
0	0	1	0
0	0	0	0
0	0	0	0
0	0	1	1
0	0	1	0
0	1	0	0
0	1	0	0
0	1	0	0
0	1	0	0
0	0	1	1
0	0	1	1
0	0	1	1
0	0	0	0
0	1	0	0
0	0	0	0
0	0	0	0
0	0	0	0
0	0	0	0
0	0	1	0
0	0	0	0
0	0	0	0
0	0	0	0
0	0	0	0
0	0	0	0
0	0	1	1
0	1	0	0
0	0	0	0
1	0	0	0
0	0	0	0
0	0	1	1
0	0	1	1

0	0	0	0
1	0	0	0
1	0	0	0
0	0	0	0
0	0	0	0
0	0	0	0
0	0	0	0
0	0	0	0
0	0	0	0
0	0	0	0
0	0	0	0
0	0	0	0
0	0	0	0
0	0	1	0
0	0	0	0
0	0	0	0
0	0	0	0
0	0	0	0
0	0	0	0
0	0	0	0
0	0	0	0
0	0	0	0
0	0	0	0
0	0	0	0
0	0	0	0
0	0	0	0
0	0	0	0
0	0	0	0
0	0	0	0
0	0	0	0
0	0	0	0
0	1	0	0
0	0	0	0
0	0	0	0
0	0	0	0
0	0	0	0
0	0	0	0
0	0	0	0
0	0	0	0
0	0	0	0
0	0	0	0
0	0	0	0
0	0	0	0
0	0	0	0
0	0	0	0
0	0	0	0
0	0	0	0
0	0	0	0
0	0	0	0
0	0	0	0

0	0	0	0
0	0	0	0
0	0	0	0
0	0	0	0
0	0	0	0
0	0	0	0
0	0	0	0
0	0	0	0
0	0	0	0
0	0	0	0
0	0	0	0
0	1	0	0
0	0	0	0
0	0	0	0
1	0	0	0
1	0	0	0
0	0	1	0
0	0	0	0
0	0	0	0
0	0	0	0
0	0	0	0
0	0	0	0
0	0	0	0
0	0	0	0
0	0	1	1
0	0	1	1
0	0	1	1
0	0	1	1
0	0	1	1
0	1	0	0
0	1	0	0
0	1	0	0
0	1	0	0
0	0	0	0
0	1	0	0
0	0	0	0
0	0	1	1
1	0	0	1
1	0	1	1
0	0	0	0
0	0	1	1
0	0	0	0
0	0	0	0
0	1	0	0
0	0	0	0
0	0	0	0
0	0	0	0
0	0	0	0
0	0	0	0
0	0	0	0

0	0	0	0
0	0	0	0
0	0	0	0
0	1	0	0
0	0	0	0
0	0	1	1
0	0	0	0
0	0	0	0
0	0	1	1
0	0	1	1
0	0	1	1
0	0	1	1
0	0	0	0
0	0	0	0
0	0	0	0
0	0	1	1
0	0	0	0
0	0	0	0
1	0	0	0
1	0	0	0
0	0	0	0
1	0	0	0
0	0	0	0
0	0	0	1
0	0	0	0
0	0	0	0
0	0	0	0
0	0	0	0
0	0	0	0
0	0	0	0
0	0	0	0
0	0	0	0
0	0	0	0
0	0	0	0
0	0	0	0
0	0	0	0
0	0	0	0
0	0	0	0
0	0	0	0
0	0	0	0
1	0	0	0
1	0	0	0
0	0	0	0
0	0	0	0
0	0	0	0
0	0	0	0
0	0	0	0
0	0	0	0
0	0	0	0
0	0	0	0

0	0	0	0
0	0	0	0
0	0	0	0
0	0	0	0
0	0	0	0
0	0	0	0
0	0	0	0
0	0	0	0
0	0	0	0
0	0	0	0
0	0	0	0
0	0	0	0
0	1	0	0
0	1	0	0
0	1	0	0
0	0	0	0
0	0	0	0
0	0	0	0
0	0	0	0
0	0	0	0
0	0	0	0
0	0	0	0
0	0	0	0
0	0	0	0
0	0	0	0
0	0	0	0
0	0	0	0
0	0	0	0
0	0	0	0
0	0	1	1
0	0	1	1
0	0	1	1
0	0	1	1
0	0	1	1
0	0	1	1
0	0	1	1
0	0	1	1
0	0	1	1
0	0	1	1
0	0	1	1
0	0	0	0
0	0	0	0
0	0	0	0
0	0	0	0
0	0	0	0
0	0	0	0
0	0	0	0
0	0	0	0
0	0	0	0
0	0	0	0

0	0	0	0
0	0	0	0
0	0	0	0
0	0	0	0
0	0	0	0
0	1	0	0
0	0	0	0
0	0	0	0
0	0	0	0
0	0	0	0
0	0	0	0
0	0	0	0
0	0	0	0
0	0	0	0
0	0	0	0
0	0	0	0
0	0	0	0
0	0	0	0
0	0	0	0
0	0	0	0
1	0	0	0
0	0	0	0
0	0	0	0
0	0	0	0
0	0	0	0
0	0	0	0
0	0	0	0
0	0	0	0
0	0	0	0
0	0	0	0
0	0	0	0
0	0	0	0
0	0	0	0
0	0	1	0
0	0	1	0
0	0	1	0
0	0	0	0
0	0	0	0
0	0	0	0
0	0	0	0
0	0	0	0
0	0	0	0
0	0	0	0
0	0	0	0
0	0	0	0
0	0	0	0
0	0	0	0
0	0	0	0
0	0	0	0
0	0	0	0

0	0	0	0
0	0	0	0
0	0	0	0
0	0	0	0
0	0	0	0
0	0	0	0
0	0	0	0
0	0	0	0
0	0	0	0
0	0	0	0
0	0	0	0
0	0	0	0
0	0	0	0
0	0	0	0
0	0	0	0
0	0	0	0
0	0	0	0
0	0	0	0
0	0	0	0
0	1	0	0
0	0	0	0
0	0	0	0
0	0	0	0
0	0	0	0
0	0	0	0
0	0	0	0
0	0	0	0
0	0	0	0
0	0	0	0
0	0	0	0
0	0	0	0
0	0	0	0
0	0	0	0
0	0	0	0
0	0	0	0
0	0	0	0
0	0	0	0
0	0	0	0
0	0	0	0
0	0	0	0
0	0	0	0
0	0	0	0
0	0	0	0
0	0	0	0
0	0	0	0
0	0	0	0
0	0	0	0
0	0	0	0
0	0	0	0
0	0	0	0

0	0	0	0
0	0	0	0
0	0	0	0
0	0	0	0
0	0	0	0
0	0	0	0
0	0	0	0
0	0	0	0
0	0	0	0
0	0	0	0
0	0	0	0
0	0	0	0
0	0	0	0
0	0	0	0
0	0	0	0
0	0	0	0
0	0	0	0
0	0	0	0
0	0	0	0
0	0	0	0
0	0	0	0
0	0	0	0
0	0	0	0
0	0	0	0
0	0	0	0
0	0	0	0
0	0	0	0
0	0	0	0
0	0	0	0
0	0	0	0
0	0	0	0
0	0	0	0
0	0	0	0
0	0	0	0
0	0	0	0
0	0	0	0
0	0	0	0
0	0	0	0
0	0	0	0
0	0	0	0
0	0	0	0
0	0	0	0
0	0	0	0
0	0	0	0
0	0	0	0
0	0	0	0
0	0	0	0
0	0	0	0
0	0	0	0
0	0	0	0

0	0	0	0
0	0	0	0
0	0	0	0
0	0	0	0
0	0	0	0
0	0	0	0
0	0	0	0
0	0	0	0
0	0	0	0
0	0	0	0
0	0	0	0
0	0	0	0
0	0	0	0
0	0	0	0
0	0	0	0
0	0	0	0
0	0	0	0
0	0	0	0
0	0	0	0
0	0	0	0
0	0	0	0
0	0	0	0
0	0	0	0
0	0	0	0
0	0	0	0
0	0	0	0
0	0	0	0
0	0	0	0
0	0	0	0
0	0	0	0
0	0	0	0
0	0	0	0
0	0	0	0
0	0	0	0
0	0	0	0
0	0	0	0
0	0	0	0
0	0	0	0
0	0	0	0
0	0	0	0
0	0	0	0
0	0	0	0
0	0	0	0
0	0	0	0
0	1	0	0
0	0	0	0
0	0	0	0
0	0	0	0
0	0	0	0
0	0	0	0

0	0	0	0
0	0	0	0
0	0	0	0
0	0	0	0
0	0	0	0
0	0	0	0
0	0	0	0
0	0	0	0
0	0	0	0
0	0	0	0
0	0	0	0
0	0	0	0
0	0	0	0
0	0	0	0
0	0	0	0
0	0	0	0
0	0	0	0
0	0	0	0
0	0	0	0
0	0	0	0
0	0	0	0
0	0	0	0
0	0	0	0
0	0	0	0
1	0	0	0
1	0	0	0
1	0	0	0
0	0	0	0
0	0	0	0
1	0	0	0
1	0	0	0
1	0	0	0
1	0	0	0
1	0	0	0
1	0	0	0
1	0	0	0
1	0	0	0
1	0	0	0
0	0	1	0
0	0	1	0
0	0	1	0
0	0	0	0
0	0	0	0
0	0	0	0
0	0	0	0
0	0	0	0
0	0	0	0
0	0	0	0
